# Exercise training modalities in prediabetes: a systematic review and network meta-analysis

**DOI:** 10.3389/fendo.2024.1308959

**Published:** 2024-02-19

**Authors:** Hang Zhang, Yuting Guo, Guangshun Hua, Chenyang Guo, Simiao Gong, Min Li, Yan Yang

**Affiliations:** ^1^ School of Health Preservation and Rehabilitation, Chengdu University of Traditional Chinese Medicine, Chengdu, China; ^2^ Department of Rehabilitation Medicine, The Third Affiliated Hospital of Sun Yat-sen University, Guangdong, China; ^3^ Medical Department of The Third Affiliated Hospital of Chengdu University of Traditional Chinese Medicine, Chengdu, China

**Keywords:** exercise, prediabetic state, glycemic control, weight loss, cardiovascular risk factors, network meta-analysis

## Abstract

**Background:**

Lifestyle modification based on exercise intervention is still the primary way to delay or reverse the development of diabetes in patients with prediabetes. However, there are still challenges in setting up a detailed exercise prescription for people with prediabetes. This study mainly ranks exercise prescriptions by comparing the improvement of glucose and lipid metabolism and the level of weight loss in patients.

**Method:**

All studies on exercise intervention in prediabetes were identified by searching five electronic databases. Risk assessment and meta-analysis were performed on eligible studies.

**Results:**

Twenty-four studies involving 1946 patients with prediabetes and seven exercise intervention models were included in the final analysis. The meta-analysis showed that exercise of any type was more effective for glycemic control in prediabetes than no exercise. However, the changes in blood glucose were moderate. In prediabetes, combining moderate-intensity aerobic exercise with low-to moderate-load resistance training showed the most significant improvements in glycosylated hemoglobin (HbA1c), body mass index (BMI), body weight (BW), total cholesterol (TC), and low-density lipoprotein cholesterol (LDL) (P-score=0.82; 0.70; 0.87; 1; 0.99), low-to moderate-load resistance training showed the most significant improvements in fasting blood glucose (FBG) (P-score=0.98), the vigorous-intensity aerobic exercise showed the most significant improvements in 2-hour post-meal blood glucose (2hPG) and systolic blood pressure (SBP) (P-score=0.79; 0.78), and moderate-intensity aerobic exercise showed the most significant improvements in diastolic blood pressure (DBP) (P-score=0.78).

**Conclusion:**

In summary, moderate-intensity aerobic exercise, low-to moderate-load resistance training and the combination of both have beneficial effects on glycemic control, weight loss, and cardiovascular health in patients with prediabetes. These findings provide valuable guidance for rehabilitation clinicians and patients alike to follow.

**Systematic review registration:**

https://www.crd.york.ac.uk/PROSPERO/, identifier CRD 42021284922.

## Graphical Abstract

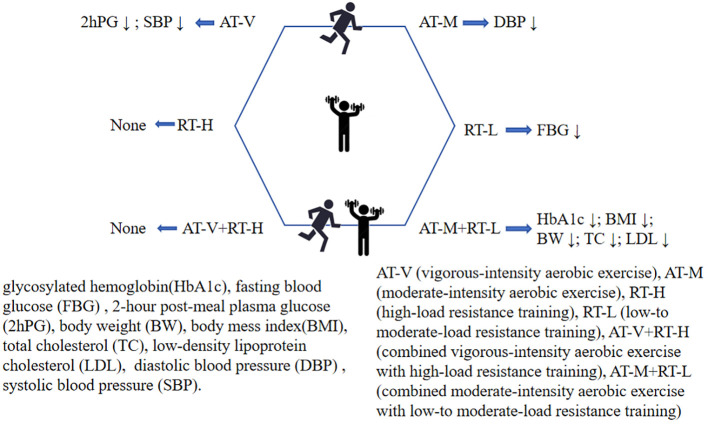

## Key points

In this network meta-analysis, we report that for prediabetes, moderate-intensity aerobic exercise combined with low-to moderate-load resistance training, moderate-intensity aerobic exercise, and low-to moderate-load resistance training should be considered the top three exercise interventions for improving glycemic control, weight control, and cardiovascular risk factors.

Specifically, moderate-intensity aerobic exercise combined with low-to moderate-load resistance training showed the best results in reducing glycosylated hemoglobin (HbA1c), body mass index (BMI), body weight (BW), total cholesterol (TC) and low-density lipoprotein cholesterol (LDL); and low- to moderate-load resistance training was more effective at improving fasting blood glucose (FBG) than moderate-intensity aerobic exercise combined with low-to moderate-load resistance training. In addition, vigorous-intensity aerobic exercise significantly reduced systolic blood pressure (SBP) and 2-hour post-meal blood glucose (2hPG).

In the subgroup analysis, we found that FBG reduction was more significant in patients older than 60. Moreover, improvements in HbA1c, TC and BMI increased with training time, but the optimal intensity and type of exercise remained moderate-intensity aerobic exercise combined with low-to moderate-intensity resistance training.

## 1 Background

Prediabetes is a high-risk state for diabetes, usually comprising impaired fasting glucose (IFG), impaired glucose tolerance (IGT), or both (CGI). It is also defined as a blood glucose indicator above normal but below the threshold for diabetes. Genetic factors, physical inactivity, and shifts in the structure of diets, including high in sugar, fat and low in dietary fiber, have increased the prevalence of prediabetes globally, and experts predict that by 2030, more than 470 million people will have prediabetes (1, 2).

It has been reported that 5%∼10% of prediabetes develops into diabetes each year and up to 70% of prediabetes eventually develops into diabetes (3, 4). In addition to the ultra-high rate of diabetes conversion, prediabetes is also associated with an increased risk of early and chronic kidney disease, autonomic neuropathy, and cardiovascular disease (5). Therefore, as the prime time to interrupt type 2 diabetes, prediabetes should be subject to early intervention to avoid the potential effects of prediabetes itself and to prevent it from developing into diabetes or to mitigate some of the potential consequences of developing diabetes.

Current studies have all demonstrated that lifestyle modification and pharmacological interventions play a significant role in delaying the progression of prediabetes to diabetes. In terms of the effectiveness of interventions for prediabetes, medications such as metformin and acarbose are less effective than lifestyle enhancement. Moreover, the choice of pharmacological interventions appears to put patients at higher risk and financial stress. Therefore, lifestyle is currently the intervention of choice for prediabetes (6, 7). As an essential component of lifestyle interventions, exercise interventions have been shown to aid the benign regression of prediabetes by improving insulin resistance, blood glucose levels, lipid metabolism, inflammatory response, and gut flora.

Current guidelines recommend that moderate intensity aerobic exercise is preferred in prediabetes, supplemented by resistance training, when possible, but do not recommend at an appropriate intensity. Aerobic, resistance, and combination training are now widely used in the prediabetic population. A comprehensive comparison of these intervention types has been made, recommending aerobic and combination exercise as the best type of exercise for the prediabetic population (8). However, exploring the benefits of exercise beyond exercise intensity has many limitations and risks, and vague recommendations for exercise prescription are often not understood by patients, which also makes exercise difficult to maximize treatment effects. Therefore, based on the complete concept of exercise prescription setting, this study comprehensively considered four aspects of exercise type, exercise frequency, exercise intensity, and exercise time, and conducted a network meta-analysis of exercise intervention modalities for prediabetes to (I) evaluate the comparative effects of different exercise prescriptions on weight loss, glycemic regulation and, cardiovascular fitness in prediabetes, (II)to contribute to the development of a hierarchy of exercise interventions for prediabetes.

## 2 Methods

This work was conducted by Preferred Reporting Items for Systematic Reviews and Meta-Analyses for Network Meta-Analyses (PRISMA-NMA) (9). In addition, this study has been registered with PROSPERO, under CRD42021284922. The PRISMA Checklist and Protocol were presented in **Additional File 1**: **Appendix 1**, **2**.

### 2.1 Search strategy

We searched PubMed, Embase, Web of Science, Cochrane Library, and Sport Discus for relevant studies from their inception dating to October 2022. The search strategy was constructed around the PICOS tool: (P) Participants: patients with prediabetes; (I) Intervention: Detailed exercise prescription interventions; (C) Comparisons: other exercise prescription or no exercise control; (O) Outcomes: fasting blood glucose (FBG), 2-hour post-meal blood glucose (2hPG), glycosylated hemoglobin (HbA1c), body weight (BW), etc.; (S) Study type: RCTs. A complete list of the search terms is shown in **Additional File 1**: **Appendix 3**. In addition, we scanned the references of the included articles to find those that met the inclusion criteria. HZ screened and identified the results of the research to exclude duplicate records. Yt G and HZ independently screened the titles and abstracts of the remaining studies against inclusion and exclusion criteria. HZ and Yt G independently screened the full-text articles with a “yes, unsure, or no” approach. A kappa statistic was used to calculate the level of agreement between Yt G and HZ for both abstracts and full-text screening (10). A Kappa value between 0.40 and 0.59 was considered a fair agreement, 0.60 to 0.74 as a good agreement, and greater than 0.75 as an excellent agreement (11). Any disagreements that arose during this process were negotiated by the broader team.

### 2.2 Inclusion and exclusion criteria

#### 2.2.1 The inclusion criteria were performed as follows

Type of participants: We included studies enrolling participants with prediabetes aged ≥18 years, excluding patients with other chronic diseases, children, adolescents, or pregnant women. Patients diagnosed with prediabetes according to the ADA (American Diabetes Association) and WHO (World Health Organization) criteria: FBG:100~125mg/dL or HbA1c 5.7% to 6.4%.

Currently, there has yet to be a consensus on the diagnostic criteria for prediabetes. According to the ADA practice standards, prediabetes is defined as FBG 100-125 mg/dL (5.6-6.9 mmol/L) or HbA1c 5.7-6.4% (39-46 mmol/mol), while the WHO defines it as an FBG of 110 to 125 mg/dL. Furthermore, the critical levels of HbA1c differed between the guidelines. The results of a large 10-year community-based prospective cohort study in the United States showed that HbA1c 5.7% to 6.4% had reasonable diagnostic specificity and had a substantial predictive value for the risk of cardiovascular events and mortality (12). Criteria for the diagnostic use of prediabetes in adults (**Table 1**).

**Table 1:** Criteria for the diagnostic use of prediabetes in adults.

	IFG	IGT	CGI
FBG (mmol/L)	6.1~7.0	<6.1	6.1~7.0
2h-PG (mmol/L)	<7.8	7.8~11.1	7.8~11.1
HbA1c (%)	5.7~6.4		

Type of interventions: We focused on the following 7 exercise training modalities, and each category was designed according to the principles of frequency, intensity, duration, and type of exercise prescription and American College of Sports Medicine (ACSM) estimates of cardio and resistance exercise intensity: AT-V (vigorous-intensity aerobic exercise), AT-M (moderate-intensity aerobic exercise), RT-H (high-load resistance training), RT-L (low-to moderate-load resistance training), AT-V+RT-H (combined vigorous-intensity aerobic exercise with high-load resistance training), AT-M+RT-L (combined moderate-intensity aerobic exercise with low-to moderate-load resistance training) and CON (no exercise). The definition of each intervention is shown in **Additional File 1**: **Appendix 4**.

Type of outcomes: Outcomes of interest included glycemic control [including glycosylated hemoglobin (HbA1c), fasting blood glucose (FBG) and 2-hour post-meal plasma glucose (2hPG)], weight loss [including body weight (BW) and body mass index (BMI)] and cardiovascular risk factors [including total cholesterol (TC), low-density lipoprotein cholesterol (LDL), high-density lipoprotein cholesterol (HDL), diastolic blood pressure (DBP) and systolic blood pressure (SBP)].

Type of design: Randomized controlled trials (RCTs).

#### 2.2.2 The exclusion criteria were performed as follows

(1) non-randomized design; (2) in regard to RCTs with repeated publications or apparent duplication of data, only one study with more complete data was kept; (3) means and standard deviations were unavailability in the results, and authors did not reply to our requests for data; (4) vague descriptions of exercise modalities; (5) full text of the study could not be available through relevant databases and other means.

### 2.3 Data extraction

Relevant publication information; (1) number; (2) author; (3) year of publication; (4) country; (5) sample size; (6) mean age; (7) the details of exercise prescription were recorded using the FITT principle (frequency, type, time and intensity); (8) outcomes (FBG, 2hPG, HbA1c, Weight, BMI, TC, LDL, HDL, SBP, DBP). If the original study reported a standard error in the experimental and control groups, the standard deviation was calculated by the formula: standard deviation (SD) = standard error (SE) × $\sqrt{\text{N}}$ . If both were missing, we would estimate SD based on the confidence interval, t-value, quartile, range, or p-values as described in section 7.7.3 of the Cochrane Handbook for Systematic Reviews.

### 2.4 Risk of bias assessment

HZ and Yt G independently assessed the risk of included studies based on the bias 2.0 Tool (37). Examined domains; (1) randomization process; (2) deviations from the intended interventions; (3) missing outcome data; (4) measurement of the outcome; (5) selection of the reported results.

### 2.5 Statistical analysis

The net Meta package of R3.6.3 software was applied to perform an NMA combining direct and indirect comparisons based on the Frequentist model (38, 39). Arm-level data was imported into the R software in CSV format. Allowing for the consistent rating scales or units of each outcome, the mean difference (MD) was chosen to measure the effect sizes. The effect sizes were then synthesized using a random-effects NMA model. We presented the summary MD, 95% credible intervals (CrIs) for all pairwise comparisons in the league table. Furthermore, we showed the results of comparison of each exercise modality and control group in the form of a forest plot. Besides, we used p-score to rank exercise modalities based on the improvement of glucose and lipid metabolism and the level of weight loss (40). A higher p-score indicates a greater degree of improvement. For different clinical trials, it is necessary to ensure the consistency of their baseline levels. Therefore, prior to analysis of the results, we evaluated the transitivity assumption by comparing the distribution of potential effect modalities (year of publication, sample size, mean age, percentage of male) (**Additional File 1**: **Appendix 5**) across the studies. Tau square (τ^2^) test and p-value were used to qualitatively analyze the statistical heterogeneity between the studies. Larger τ2 and smaller p-value indicate greater possibility of heterogeneity. Moreover, I^2^ is a parameter for quantitative analysis of the heterogeneity between all results. I^2^ < 25% means low heterogeneity; 25%-50% means moderate heterogeneity; I^2^ > 75% means high heterogeneity. We also used global and local methods to test the inconsistency of these results. Design-by-treatment test was used to evaluate inconsistency statistically for global inconsistency and separate indirect from direct evidence (SIDE test) for local inconsistency (41, 42). The potential sources of heterogeneity (publish year, mean age, percentage of males, sample size, exercise period, exercise frequency, the single session of exercise) were explored by network meta-regression through the R3.6.3 gemtc package. Subgroup analysis was performed by the forest spot package of R3.6.3 software. In addition, the adjusted funnel plot was compared to assess the risk of publication bias under specific circumstances. Egger’s test suggests publication bias when p< 0.05.

## 3 Results

**Figure 1:**
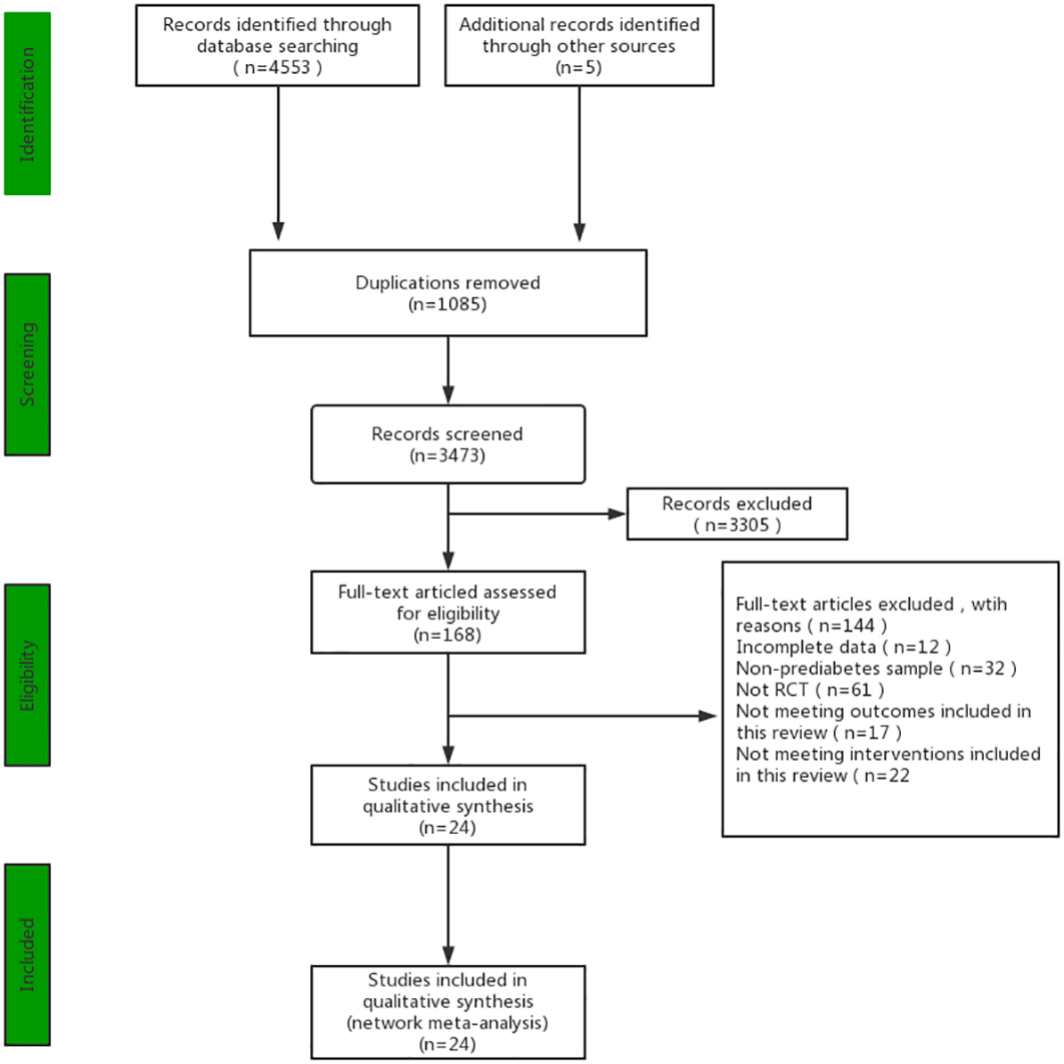
PRISMA Flow diagram of the search process for studies.

A total of 4553 studies were identified according to the search strategy. After initially identifying titles and abstracts, the remaining 168 studies were screened for full text. The inter-rater reliability between the two reviewers for both abstract screening (K=0.74) and full-text screening (K=0.71) was considered good. Finally, 24 studies with 1946 participants were included in this study (**Figure 1**). The studies were conducted in America (n=6), China (n=6), Iran (n=1), Finland (n=4), Sweden (n=2), Chile (n=1), Netherlands (n=1), Germany (n=1), Austria (n=1) and Canada (n=1). Furthermore, in 24 studies, 8 with 3 arms, 14 with 2 arms, and 2 with 4 arms. 2 of the studies looked only at women, 2 at men, and the remaining 22 included both men and women (**Table 2**).

**Table 2:** Characteristic of included studies.

Author Year	country	Sample Size (men)	Age (Mean ± SD)	Exercise Prescription	Results
Tahereh 2019 (13)	Iran	AT-M:136 (59) CON:136 (51)	AT-M:51.3 ± 11.2 CON:53.6 ± 9.4	AT-M:60%~70%VO_2max_,50min/day,3times/week,16weeks	a,d,e
Yan J 2019 (14)	China	AT-M:35 (10) RT-L:35 (15) CON:35 (15)	AT-M:64.23 ± 5.75 RT-L:62.06 ± 8.11 CON:60.31 ± 7.56	AT-M:60%~70%HR_max_, 50min/day, 3times/week,12weeks RT-L:60%1RM,50min/day, 3times/week,12weeks	a,b,c,e,f,g,h
Dai X 2019 (15)	China	AT-M:34 RT-L:31 AT-M+RT-L:37 CON:35	AT-M:51 ± 5 CON:58 ± 3	AT-M:60%~70%HRmax,60min/day, 3times/week,2years RT-L:60%~80%1RM,60min/day, 3times/week,2years AT-M+RT-L:combined AT-M with RT-L	a, b,c,d,f,g,h
Kramer 2018 (16)	USA	AT-M: 88 (30) CON: 46 (14)	AT: -M:62.8 ± 12.1 CON:61.9 ± 11.9	AT-M:60%~70%HRmax,50min/day,3times/week,18months	a,c,d,e,f,g,h,i,j
Slentz 2016 (17)	USA	AT-M: 40 (17) AT-V: 38 (15)	AT-M:61.4 ± 7.1 AT-V:60.4 ± 7.0	AT-M: 50%VO_2reserve_, 30 min/day, 3 times/week,6 months AT-V: 75% VO_2reserve_, 60 min/day, 3 times/week,6 months	a,b,d,f,g,h
Gidlund 2016 (18)	Finland	RT-L: 20 (20) AT-M: 18 (18) CON:17 (17)	RT-L:54 ± 6.2 AT-M:56 ± 5.6 CON:54 ± 6.9	RT-L:50%1RM,60min/day,3times/week,12weeks AT-M:55%~75%HRR,60min/day,3times/week,12weeks	b,c
Liao 2015 (19)	China	AT-M:60 (33) CON:60 (35)	AT-M:42.4 ± 5.8 CON:44.1 ± 6.6	AT-M:60%~70%HRmax,30min/day,5times/week,12weeks	a,c,e,f,g,h,i,j
Herrzig 2014 (20)	Finland	AT-M:33 (9) CON:35 (9)	AT-M:58.1 ± 9.9 CON:59.5 ± 10.8	AT-M:60%~70%HRmax,60min/day,3times/week,12weeks	a,b,d,e,f,g,h,i,j
Venojarvi 2013 (21)	Finland	AT-V:39 (39) RT-H:36 (36) CON:40 (40)	AT-V:55 ± 6.2 RT-H:54 ± 6.1 CON:54 ± 7.2	AT-V:65%~75%HRR,60min/day, 3times/week,12weeks RT-H:75%~85%1RM,60min/day, 3times/week,12weeks	a,b,c,d,f,g,h,i,j
Fritz 2013 (22)	Sweden	AT-M:14 (5) CON:21 (10)	AT-M:59.1 ± 6.2 CON:61.8 ± 3.4	AT-M:60%~70%HRmax,60min/day, 5times/week,16weeks	a,b,c,d,e,f,g,h,i,j
Hansen 2012 (23)	Sweden	RT-H:9 (2) RT-L:9 (2) CON:9 (2)	RT-H:59.1 ± 6.2 RT-L:61.8 ± 3.4 CON:56.1 ± 4.4	RT-H:85%1RM,60min/day, 3times/week,16weeks RT-L:65%1RM,60min/day, 3times/week,16weeks	a,b
Alvarez 2012 (24)	Chile	AT-V:12 (0) RT-H:8 (0) AT-V+RT-H:10 (0) Con:13 (0)	AT-V:39.2 ± 9.5 RT-H:33.9 ± 9.3 AT-V+RT-H:43.3 ±8.1 CON:40.1 ± 11.4	AT-V:75%VO2max,50min/day,3times/week,22weeks RT-H:75%~85%1RM,60min/day, 3times/week,22weeks AT-V+RT-H: combined AT-V with RT-H	a,d,e,i,j
Burtscher 2009 (25)	Austria	AT-M+RT-L: 18 (8) CON: 18 (8)	AT-M+RT-L:55.8 ± 5.5 CON:59.1 ± 7.8	AT-M+RT-L:70%HRmax+70%1RM,60min/day, 3times/week,12months	a,d,e,f,g,i,j
Desch 2010 (26)	Germany	AT-V:14 (11) CON: 12 (8)	AT-V:62.3 ± 6.2 CON:62.3 ± 6.5	AT-V:75%VO2max,90min/day,3times/week,6months	a,b,c,e
Eriksson 1998 (27)	Finland	AT-M:7(3) RT-L:7(4) CON:8(8)	AT-M:60 ± 5 RT-L:40 ± 3 CON:60 ± 5	AT-M:60%VO2max,60min/day,3times/week,10weeks RT-L:50%~60%1RM,60min/day,3times/week,10weeks	a,e,f,g,i,j
Malin 2012 (28)	USA	AT-M+RT-L: 8 (3) CON: 8 (2)	AT-M+RT-L:45.4 ± 8.0 CON:49.8 ± 10.9	AT-M+RT-L:70%HRmax+70%1RM,60~70min/day,3times/week,12weeks	a,d
Marcell 2005 (29)	USA	AT-V:20 AT-M:17 CON:14	AT-V:47.2 ± 9.2 AT-M:44.4 ± 6.5 CON:44.1 ± 9.5	AT-V:80%~90%VO_2_max,30min/day,5times/week,16weeks AT-M:3.5MET,30min/day,5times/week,16weeks	d
Marcus 2009 (30)	USA	RT-H:10 (0) CON:6 (0)	RT-H:56.3 ± 6.4 CON:53.2 ± 6.5	RT-H:85%1RM,30min/day,3times/week,12weeks	a
Roumen 2008 (31)	Dutch	AT-V:54 (30) CON: 52 (28)	AT-V:58.4 ± 6.8 CON:54.2 ± 5.8	AT-V:70%VO2max,30min/day,3times/week,3years	b,c,d,e,f,g,h,i,j
Rowan 2017 (32)	Canada	AT-M:10 AT-V:11	AT-M:47.7 ± 6.93 AT-V:53.6 ± 8.21	AT-M:60%~70%HRR,30min/day,3times/week,12weeks AT-V:90%HRR,30min/day,3times/week,12weeks	a,b,e
Yuan 2020 (33)	China	AT-M:83 (24) RT-L:82 (30) Con:83 (33)	AT-M:60.93 ± 5.71 RT-L:59.91 ± 5.92 CON:60.73 ± 5.83	AT-M:60%~70%HRmax,60min/day,3times/week,6months RT-L:50%~60~1RM,60min/day,3times/week,6months	a,b,c,d,e,f,g,i,j
sulin 2017 (34)	China	AT-V:29 (6) Con:29 (7)	AT-V:59 ± 4.4 CON:60 ± 3.4	AT-V:60%~75%VO2max,60min/day,3times/week,6months	a,b,c,d
Lin 2021 (35)	China	AT-M:43 (3) RT-L:42 (4) CON:43 (4)	AT-M:60.35 ± 4.29 RT-L:60.12 ± 3.97 CON:59.94 ± 4.40	AT-M:60%~70%HRmax,50min/day,3times/week,12months RT-L:60%~80%1RM,50min/day,3times/week,12months	a,b,c
Nicole 2019 (36)	USA	AT-V:17 AT-M:12	AT-V:45.7 ± 4.4 AT-M:50.8 ± 4.4	AT-V:79.8 ± 3.3%HRmax,60min/day,3times/week,16weeks AT-M:53.1 ± 2.3%HRR,60min/day,3times/week,16weeks	a,c,d,e,i,j

AT-M, Aerobic training of moderate intensity; AT-V, Aerobic training of vigorous intensity; RT-L, Resistance training of low to moderate load; RT-H, Resistance training of high load; AT-V+RT-H, Combined vigorous intensity aerobic exercise with high load resistance training; AT-M+RT-L, Combined moderate intensity aerobic exercise with low to moderate load resistance training; CON, Control; VO2max, Maximal Oxygen Consumption; HRmax, Maximal Heart Rate; HRR, Heart rate reserve; 1RM, one-repetition maximum; MET, Metabolic equivalent of energy; a, FBG; b, 2hPG; c, HbA1c; d, Weight; e, BMI; f, TC; g, SBP; h, DBP; i, HDL; j, LDL.

In terms of exercise categories, 234 participants (12.02%) were included in the AT-V category, 630 participants (32.37%) in the AT-M, 63 participants (3.24%) in RT-H, 226 participants (11.61%) in RT-L, 10 participants (0.51%) in the AT-V+RT-H and 63 participants (3.24%) in the AT-M+RT-L. The remaining 720 participants were controls (no exercise). Intensity, duration, and frequency of exercise interventions were reported in all studies, 23 of which lasted more than 12 weeks, 1 of which lasted 10 weeks, and all of which had a frequency of ≥ 3 times per week.

### 3.1 Risk of bias assessment

**Figure 2:**
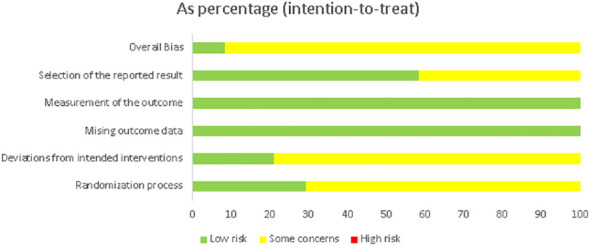
Summary of the risk of bias assessment in the individual domains of the included studies.

The summary data of ROBs assessment were presented in **Figure 2** and the ROBs assessments for each study were presented in **Additional File 1**: **Appendix 6**. Overall, there were no high-risk studies here.

### 3.2 Network meta-analysis

#### 3.2.1 Glycemic control

**Figure 3:**
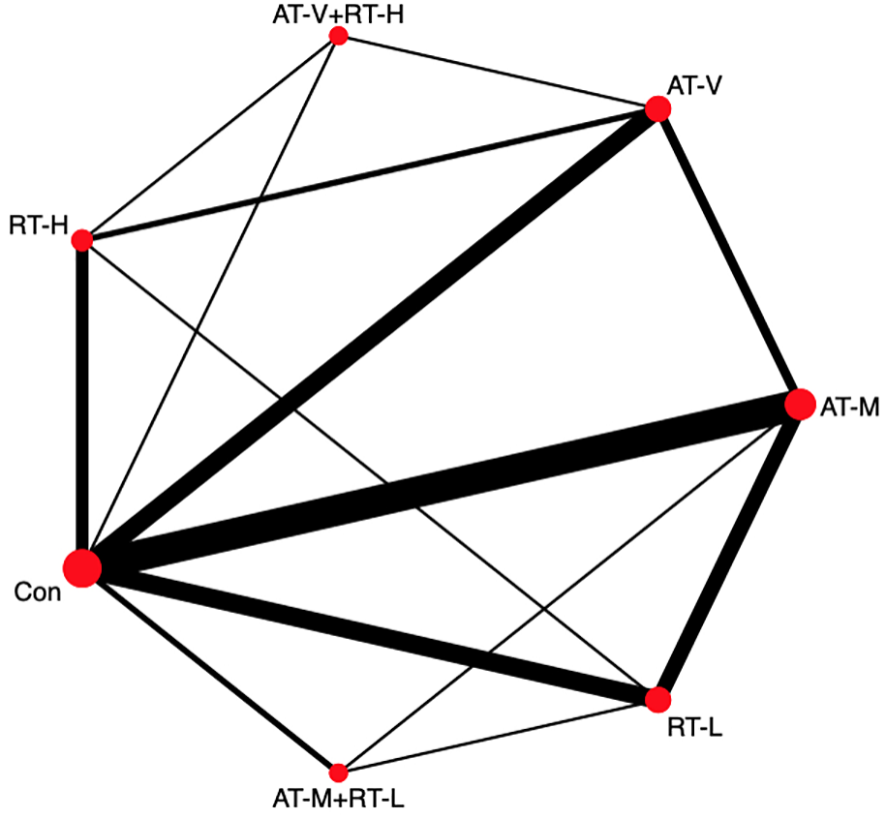
Network plot of FBG.

22 studies (91.6%) with 1761 patients (90.5%) assessed FBG and were eligible for NMA (**Figure 3**). Compared to no exercise, low-to moderate-load resistance training (-0.48mmol/L,95%CI: -0.65mmol/L~-0.32mmol/L), moderate-intensity aerobic exercise (-0.38mmol/L,95%CI: -0.51mmol/L~-0.25mmol/L), combined low-to moderate-load resistance training with moderate-intensity aerobic exercise (-0.44mmol/L,95%Cl:-0.67mmol/L~-0.21mmol/L) and vigorous-intensity aerobic exercise (-0.31mmol/L,95%Cl:-0.51mmol/L~-0.11mmol/L) showed significant reduction in FBG (**Additional File 1: Appendix 7**). Furthermore, low-to moderate-load resistance training showed the greatest potential as the best intervention to improve FBG. (P-sore=0.98, **Additional File 1: Appendix 7**). The result of Quantifying heterogeneity showed that I^2^ was 64.2% (moderate to high) (**Additional File 1: Appendix 8**. There was no significant inconsistency between direct and indirect evidence (P=0.32, **Additional File 1: Appendix 9**.

13 studies (54.1%) with 1247 patients (64.08%) assessed HbA1c and were eligible for NMA (**Additional File 1: Appendix 7**). Similar to the effects on FBG, compared to no exercise, combining low-to moderate-load resistance training with moderate-intensity aerobic exercise (-0.30, 95%Cl: -0.50~-0.10), low-to moderate-load resistance training (-0.24, 95%Cl: -0.33~-0.16), moderate-intensity aerobic exercise (-0.25, 95%Cl:-0.32~-0.17) and vigorous-intensity aerobic exercise (-0.18, 95%Cl:-0.29~-0.07) showed a significant reduction in HbA1c, and ranking probability showed that combining low-to moderate-load resistance training with moderate-intensity aerobic exercise had the most significant ability to reduce HbA1c (P-score=0.82, **Additional File 1: Appendix 7**). The result of Quantifying heterogeneity showed that I^2^ was 31.6% (moderate) (**Additional File 1: Appendix 8**).

Notably, most prediabetes shows an increase in blood glucose 2 hours after meals, which is also a marker of impaired glucose tolerance in prediabetes. 22 studies (91.6%) with 1761 patients (90.5%) assessed 2hPG and were eligible for NMA. Compared to no exercise, vigorous-intensity aerobic exercise (-0.78mmol/L, 95%Cl: -1.40mmol/L~-0.15mmol/L) and moderate-intensity aerobic exercise (-0.53mmol/L, 95%Cl: -0.88mmol/L~-0.18mmol/L) showed a significant reduction in 2hPG. Furthermore, vigorous-intensity aerobic exercise showed the greatest potential as the best intervention to improve 2hPG (P-score=0.71, **Additional File 1: Appendix 7**).

#### 3.2.2 Weight loss

15 studies (62.5%) with 1371 patients (70.45%) assessed weight loss and were eligible for NMA. Compared to no exercise, combining moderate-intensity aerobic exercise with low-to moderate-load resistance training (-3.72kg, 95%Cl: -6.34kg~-1.09kg), moderate-intensity aerobic exercise (-2.66kg, 95%Cl: -3.92kg~-1.40kg), and vigorous-intensity aerobic exercise (-2.23kg, 95%Cl: -3.62kg~-0.83kg) showed greater weight loss (**Additional File 1: Appendix 7**). In addition, combining moderate-intensity aerobic exercise with low-to moderate-load resistance training showed the most significant effectiveness in weight loss (P-score=0.87, **Additional File 1: Appendix 7**).

14 studies (58.3%) with 1214 patients (62.4%) assessed BMI and were eligible for NMA. Different from the weight loss, compared to no exercise, moderate-intensity aerobic exercise (-0.71, 95%Cl: -1.00~-0.42), and low-to moderate-load resistance training (-0.61,95%Cl: -0.97, -0.25) showed greater BMI changes (**Additional File 1: Appendix 7**).

#### 3.2.3 Cardiovascular risk factors

The Framingham Risk Score (FRS) assesses the risk of cardiovascular disease (CVD). The FRS method includes gender, age, TC, LDL, HDL, SBP and smoking status as risk factors. Therefore, TC, LDL, HDL and SBP were also selected as reference factors for the risk of developing cardiovascular disease. 12 studies with 1162 patients assessed TC, 10 with 1105 patients assessed LDL, and 12 with 1170 patients assessed SBP and DBP. And We did NMA of these except HDL due to an imbalance in the baseline.

Compared to no exercise, combining moderate-intensity aerobic exercise with low-to moderate-load resistance training (TC:-0.80mmol/L, 95%Cl: -1.13mmol/L~-0.46mmol/L; LDL: -0.62mmol/L, 95%Cl: -0.93mmol/L~-0.30mmol/L), moderate-intensity aerobic exercise (TC: -0.34mmol/L, 95%Cl: -0.49mmol/L~-0.19mmol/L; SBP: -5.18mmHg, 95%Cl: -8.05mmHg~-2.31mmHg; DBP: -3.43mmHg, 95%Cl: -5.39mmHg~-1.46mmHg), vigorous-intensity aerobic exercise (TC: -0.33mmol/L, 95%Cl: -0.56mmol/L~-0.11mmol/L; SBP: -7.54mmHg, 95%Cl: -11.61mmHg~-3.47mmHg), and low-to moderate-load resistance training (TC: -0.34mmol/L, 95%Cl: -0.54mmol/L~-0.15mmol/L; SBP: -5.39mmHg,95%Cl:-10.15mmHg~-0.64mmHg; LDL:-0.29mmol/L,95%Cl:-0.48mmol/L~-0.10mmol/L) showed better improvement in TC, LDL, SBP, and DBP. (**Additional File 1: Appendix 7**).

Combining moderate-intensity aerobic exercise with low-to moderate-load resistance training showed the most significant improvements in TC and LDL (P-score=1.00, 0.99 respectively, **Additional File 1: Appendix 7**), the vigorous-intensity aerobic exercise showed the most significant improvements in SBP (P-score=0.78, **Additional File 1: Appendix 7**), and moderate-intensity aerobic exercise showed the most significant improvements in DBP (P-score=0.74, **Additional File 1: Appendix 7**).

### 3.3 Meta-regression and subgroups analysis

Since our results had moderate to high heterogeneity, we performed a meta-regression of the sources of heterogeneity (year of publication, mean age, percentage of males, sample size, exercise period, exercise frequency, and the single session) on all outcomes. We found that mean age might significantly affect FBG, the sample size might affect 2hPG, and the exercise period might affect HbA1c, BMI, and TC (**Additional File 1: Appendix 10**), so we performed a subgroup analysis of the above. Results showed that FBG tends to be significantly reduced in elderly over 60 years, and the reduction of HbA1c, BMI, and TC tended to be greater in longer exercise period (**Additional File 1: Appendix 11**). In addition, we re-analyzed all results after adjusting all potential sources of heterogeneity to the median value, and the results after re-analysis did not conflict with our conclusions either (**Additional File 1: Appendix 10** Finally, our comparison-adjusted funnel plot had good symmetry for all outcomes, and the results of Egger’s test (FBG=0.775; 2hPG=0.440; HbA1c=0.218; BMI=0.974; Weight=0.966; TC=0.751; SBP=0.749; DBP=0.943; LDL=0.880) showed that no small study effect were found (**Additional File 1: Appendix 12**). Overall, the stability of our key findings was not a source of concern.

## 4 Discussion

### 4.1 Summary of evidence

This systematic review and meta-analysis revealed that combining moderate-intensity aerobic exercise with low-to moderate-load resistance training demonstrated the best effect in improving HbA1c, weight loss and cardiovascular risk factors; low-to moderate-load resistance training was more conducive to improving FBG. For 2hPG and blood pressure control, aerobic exercise was superior to other forms of exercise. Subgroup analysis demonstrated HbA1c, TC and BMI improved with increasing exercise duration. However, the optimal intensity and type of exercise remained moderate-intensity aerobic exercise combined with low-to moderate-intensity resistance training.

### 4.2 Comparisons with previous studies

The main pathological feature of prediabetes is impaired blood glucose regulation; both IFG and IGT have insulin resistance and abnormal insulin secretion (43, 44). IFG results from hepatic insulin resistance and impaired islet β -cell function, mainly manifested by impaired fasting blood glucose levels (44, 45). Conversely, IGT is caused by peripheral insulin resistance, notably in skeletal muscle, and is mainly characterized by impaired glucose tolerance and elevated blood glucose levels two hours after meals (44). Since IFG and IGT show distinct characteristics in pathophysiological mechanisms and clinical outcomes, therapies attempting to normalize hyperglycemia may differentially impact each phenotype.

The results of this review identified low-to moderate-load resistance training as the best intervention to improve FBG. Several clinical studies have demonstrated that resistance training reduces FBG levels in patients with diabetes as well as those with prediabetes (15, 33, 35). This is because resistance training can increase the activation of glycogen synthase (GS) through the inhibition of glycogen synthase kinase 3β (GSK3β) by AKT, which can lead to the eventual synthesis of glycogen (46–48). Second, the researchers observed that resistance training increases protein synthesis in muscle, either by activating the IGF-1/PI3K/AKT pathway or by reducing adenosine monophosphate-activated protein kinase (AMPK)-mediated mTOR inhibition (49). Increasing muscle mass can reduce muscle resistance to insulin to lower FBG levels. Finally, transiently activated AMPK may lead to the translocation of glucose transporter (GLUT-4) in skeletal muscle (49, 50), enhance fatty acid oxidation to increase glucose uptake, and ultimately improve insulin sensitivity, and lower FBG while increasing lipid clearance in the blood (47). However, there is also conflicting evidence. In Eikenberg et al’s study (51), patients with prediabetes were classified into subtypes for resistance training, and the results showed no improvement in FBG in patients with IFG. In terms of intensity, Tsai et al. (52) performed resistance training at different intensities in non-obese elderly patients with prediabetes and found that short-term high-intensity resistance training was more effective in normalizing glucose levels. It has been shown that high-intensity resistance training can increase muscle stimulation of glucose uptake and glycogen synthesis, and that an increase in GLUT-4 content may result from the greater degree of muscle fiber recruitment, leading to a consistent improvement in metabolic control and insulin sensitivity (53). However, some scholars have concluded that there is no correlation between elevated muscle mass or physical function and advancement in glycemic regulation. It is believed that this is due to the fact that better glycemic control does not rely on modifications in muscle size, but rather on changes occurring within the muscle (54). There is currently no consensus in the scientific literature regarding the aforementioned topic, which remains an area for future research. Because of the limited sample sizes of the studies that have been done, there is a lack of research on the effects of different types and intensities of exercise on different subgroups of patients with prediabetes. In addition, a number of uncontrollable factors, such as dietary habits, the timing of meals, frequency of exercise, and the design of the resistance training program, can significantly influence the results of the studies. For instance, one study found that patients with continuous glucose monitoring (CGM) decreased blood glucose levels during resistance training up to 24 hours after resistance training, but the effect may depend on meal time (55). Meanwhile, one study found that for the same intensity of resistance training, multijoint exercise recruited more adjacent muscle groups than monojoint exercise. This allows more muscle mass and fibers to be involved in the movement during multi-joint exercise, which may lead to a greater reduction in FBG (56, 57). In conclusion, further research needs to determine appropriate resistance training prescriptions for patients with different subtypes of prediabetes, particularly regarding intensity, the relationship between resistance training and meal timing, and exercise design.

For aerobic exercise, our results suggested that vigorous-intensity aerobic exercise is more effective in improving 2-hour postprandial glucose and systolic blood pressure, whereas moderate-intensity aerobic exercise improves diastolic blood pressure. To summarize, aerobic training can enhance both blood pressure control ability and insulin secretion two hours after consuming a meal. Several meta-analyses and RCTs have also confirmed that vigorous-intensity aerobic exercise significantly improves 2hPG levels, particularly in patients with IGT (35, 58, 59). Aerobic exercise increases peak oxygen consumption and can improve glucose tolerance, whole-body insulin sensitivity, and cardiovascular adaptation (32). In contrast to resistance training, aerobic exercise has no significant effect on muscle strength. Furthermore, aerobic exercise effectively induces GLUT4 enhancement factor, increasing GLUT4 expression and improved glycemic control (60, 61). Elevated 2hPG levels are typically attributed to diminished early insulin secretion, making enhancing islet β -cell function imperative for clinical significance. One study found that altered islet β -cell function was unrelated to VO_2_max (32). However, the STRRIDE study (62) showed that moderate and vigorous exercise improves β -cell function through different mechanisms in sedentary, overweight sedentary overweight adults. Vigorous intensity exercise was associated with an improvement in insulin sensitivity and a compensatory decrease in insulin secretion, whereas low and moderate intensity exercise was only associated with an improvement in insulin sensitivity. A meta-analysis demonstrated that aerobic exercise of moderate to high intensity for more than 150 minutes per week for at least 6 weeks was associated with lower SBP and DBP in patients with type 2 diabetes (63). This has been attributed to regular aerobic exercise increasing nitric oxide synthesis and action and improving endothelium-dependent vasodilation (64). However, aerobic exercise can be categorized into moderate continuous training (MCT) and high intensity interval training (HIIT). Both are associated with improvements in arterial structure and function (56, 65, 66). A recent review highlighted the potential of HIIT to improve glycemic control to a greater extent than MCT (67). Physiological studies have shown that continuous exercise can produce more reactive oxygen species, leading to increased oxidative stress, which may compromise nitric oxide bioavailability and attenuate the beneficial effects of exercise on the endothelium (68, 69). HIIT may limit these effects on nitric oxide bioavailability, as the exercise session is always followed by a recovery period (67, 70). It is also a reference point for the development of exercise prescriptions for our patients with prediabetes. In this study, the age of the population included was generally over 55 years. Therefore, we still consider moderate-intensity aerobic exercise to be the preferred type of exercise to reduce 2-hour postprandial blood glucose, and we can include short rest periods during exercise.

The efficacy of the combined aerobic and resistance training approach has been the subject of numerous studies (15, 28, 35, 71–73). This study differentiated intensity and showed that a combination of moderate-intensity aerobic exercise and low-to moderate-intensity resistance training can improve HbA1c, reduce weight, and reduce cardiovascular risk. The combination of aerobic and resistance training has been recommended by renowned institutions, including the American College of Sports Medicine, Belgian Physical Therapy Association, European Society of Cardiology and Exercise and Sports Science Australia (74–77). The results of our analyses were not only consistent with guideline-recommended interventions, but also confirmed that moderate-intensity combined exercise was the most effective modality. One study discovered that combined aerobic and resistance training had an additional effect without interference from simultaneous training (78). Combined exercise uses three methods to maximize glycemic control and improve body weight and cardiovascular outcomes. Firstly, exercise activates the insulin signaling pathway associated with AKT/PKB, increasing insulin receptor content and phosphorylation levels (61). Secondly, it can enhance the pathophysiological pathways associated with insulin resistance, upregulating the expression of GLUT 4 glucose transporters, increasing their translocation, promoting cellular glucose utilization and improving insulin resistance (61, 79, 80). These pathways comprise promoting mitochondrial biosynthesis and attenuating insulin resistance through activation of the AMPK/PGC-1α (Proliferator-activated receptor ɤ coactivator-α) pathway (81); inhibiting nuclear factor-κB (NF-κB) expression, reducing the levels of inflammatory factors such as tumor necrosis factor-alpha (TNF-α), exerting its anti-inflammatory effect (81, 82); stimulating the antioxidant mediator, nuclear erythroid 2 p45-related factor 2 (Nrf2), thereby enhancing the expression of glutathione to counteract oxidative stress caused by diabetes (83, 84); increasing the levels of galanin peptide and gene expression significantly to accelerate GLUT4 translocation and glucose uptake in myocytes and adipocytes (85). Finally, physical activity contracts skeletal muscle, enhances Ca^2+^ influx, increases osteocalcin, and boosts capillary flow to raise GLUT 4 translocation in the cell membrane, thereby promoting glucose uptake by muscle cells (72). During this process, regulation of adiponectin, visfatin, omentin-1 and leptin increases fatty acid release from adipocytes and fatty acid oxidation capacity, thereby increasing insulin sensitivity, minimizing lipid deposition in blood vessels, reducing visceral fat weight and reducing the risk of cardiovascular disease (86, 87). High-intensity resistance training combined with aerobic exercise may be difficult for elderly or obese patients with prediabetes to stick to, and they may also be less safe when exercising. Therefore, moderate-intensity aerobic exercise combined with low-to moderate-intensity resistance exercise is recommended.

### 4.3 Strengths and limitations

This study had several advantages and disadvantages. First, the review was systematic and exhaustive, with a considerable patients with prediabetes sample size (n=1946) being included, providing the ability to detect statistically significant mean differences. Second, only randomized controlled trials (RCT) were included, which is the gold standard for assessing the effectiveness of the intervention. Thirdly, the inclusion of general body morphology in the NMA outcome measures also optimized this study, as this has generally been ignored in meta-studies evaluating exercise interventions for prediabetes. However, it is essential as a predictor and associated factor in all-cause mortality.

Our review shared some limitations with the studies that it has incorporated. Although we sought to limit heterogeneity by using stringent inclusion and exclusion criteria, the study population varied in several ways (age, recruited countries, and the proportion of male and female participants). Although nearly all of the included studies were conducted nearly 3 times per week, with a 50-minute exercise intervention, the duration of the intervention varied greatly. However, when included in the analysis as covariates, the intervention duration (number of weeks) could not explain the differences in the effect size of all outcomes in the NMA. In addition, the patients in the trials we included were not followed up, so the duration of the exercise effect could not be determined. We plan to extend this investigation in future trials.

Furthermore, most included trials reported risk factor outcome measures associated with inflammation as baseline data (TC, SBP, DBP, HDL, and LDL). However, they did not report these data in their post-intervention results. Exercise is considered as a cornerstone of preventing and managing metabolic syndrome, so future studies need to design these metabolic markers as primary outcomes rather than secondary or tertiary outcomes. This NMA identified some missing evidence associated with the exercise category. Aerobic exercise remains the most commonly used intervention, with resistance training and combined training representing only 14.85% and 3.75%, respectively. Often, due to limited data on direct comparison of specific interventions, especially for resistance training versus joint training, readers should interpret these results with caution as the lack of direct evidence, which makes the analysis less reliable, suggesting the need for further studies of resistance training and joint exercise in patients with prediabetes, 16 of 24 RCT trials at moderate risk of ROB. Since participants and supervisors could not blind exercise training, the associated bias for experimenters and patients was high, and 11 studies still showed unclear randomized sequence generation bias for the gold standard of randomized controlled trials.

Overall, the quality of the studies we included in the NMA was moderate. Thus, the current NMA results should be interpreted in a conservative manner.

### 4.4 Implications and future research

The findings in our review provided strong evidence that moderate-intensity combination training is essential in improving glycemic regulation function and preventing conversion to type 2 diabetes of diabetes in patients with prediabetes, which provides new ideas for glycemic control. Furthermore, we advocate further researches to address several important issues. First, our results should be confirmed in different types of patients with prediabetes. In addition, future clinical studies should consider different types of precursor diabetes mellitus (impaired fasting blood glucose and impaired glucose tolerance) to explore and improve different exercise intervention prescriptions, and to provide theoretical support for a clinical exercise intervention in prediabetes.

## 5 Conclusions

Despite its limitations, our systematic review and meta-analysis have showcased the positive effects of moderate-intensity aerobic exercise, low-to moderate-load resistance training, and the combination of both on prediabetes. These findings can provide valuable guidance to clinicians when prescribing exercise to patients with prediabetes, and to patients when self-administering the intervention.

## Data availability statement

The datasets presented in this study can be found in online repositories. The names of the repository/repositories and accession number(s) can be found in the article/**Supplementary Material**.

## Author contributions

HZ: Writing – original draft, Writing – review & editing. YG: Writing – review & editing. GH: Writing – review & editing. CG: Writing – review & editing. SG: Writing – review & editing. ML: Writing – review & editing. YY: Writing – review & editing.
