# Supplementary material for: Exercise training modalities in prediabetes: a systematic review and network meta-analysis
Source: Front Endocrinol (Lausanne). 2024 Feb 19;15:1308959. doi: 10.3389/fendo.2024.1308959 (PMC10911289; doi:10.3389/fendo.2024.1308959)
Supplement: Supplementary file 1 [file DataSheet_1.pdf]

Article title:

Exercise training modalities in prediabetes: a systematic review and network meta-analysis

Journal name:

Frontiers in Endocrinology

Authors:

Hang Zhang[1]·Yuting Guo[1]·Guangshun Hua[1]·Chenyang Guo[1]·Simiao Gong[2]·Min Li[1]·Yan Yang[3]

Affiliation and email address of corresponding author:

The Third Affiliated Hospital of Chengdu University of Chinese Medicine, Chengdu, China. 306599761@qq.com

Supplementary

Appendix 1: PRISMA Checklist 1

Appendix 2: Protocol 8

Appendix 3: Search Strategies 22

3.1 Database: PubMed <inception to October 10 2022> 21

3.2 Database: Embase <inception to October 10 2022> 23

3.3 Cochrane 24

3.4 Database: Web of Science <inception to October 10 2022> 25

3.5 Database: Sport Discus <inception to October 10 2022> 28

Appendix 4: Definitions of physical activity types and non-exercise training control 31

Appendix 5: Assessment of the transitivity 33

5.1 Publish years 34

5.2 Mean age 35

5.3 Percentage male 36

5.4 Sample Size 37

Appendix 6: The risk of bias assessment for the individual included studies. 38

Appendix 7: Results of direct,indirect,network meta-analysis 44

7.1 Results of network mrta-analysis 44

7.2 Forest plots 63

Appendix8: Evaluation of heterogeneity 72

Appendix 9: Evaluation of inconsistency 73

Appendix10: Network Meta-Regression--changes in heterogeneity 74

10.1 Outcome of Network meta-Regression 74

10.2 FBG 75

10.3 2hPG 82

10.4 HbA1c 89

10.5 Weight 95

10.6 BMI 103

10.7 TC 110

10.8 TC 117

10.9 SBP 124

10.10 DBP 131

Appendix 11: Subgroup analysis **138**

11.1 FBG 138

11.2 2hPG 141

11.3 HbA1c 144

11.4 BMI 147

11.5 TC 150

11.6 DBP 155

Appendix 12: Publication bias **158**

12.1 FBG 158

12.2 2hPG 159

12.3 HbA1c 160

12.4 Weight 161

12.5 BMI 162

12.6 TC 163

12.7 SBP 164

12.8 DBP 165

12.9 HDL 166

12.10 LDL 167

Appendix 1: PRISMA Checklist

Section/Topic	**Item #**	Checklist Item	**Reported on Page #**
TITLE			
**Title**	1	Identify the report as a systematic review *incorporating a network meta-analysis (or related form of meta-analysis).*	1
ABSTRACT			
**Structured summary**	2	Provide a structured summary including, as applicable:<br>**Background:** main objectives<br>**Methods:** data sources; study eligibility criteria, participants, and interventions; study appraisal; and *synthesis methods, such as network meta-analysis.*<br>**Results:** number of studies and participants identified; summary estimates with corresponding confidence/credible intervals; *treatment rankings may also be discussed. Authors may choose to summarize pairwise comparisons against a chosen treatment included in their analyses for brevity.*<br>**Discussion/Conclusions:** limitations; conclusions and implications of findings.<br>**Other:** primary source of funding; systematic review<br>registration number with registry name.	1-2

INTRODUCTION			
**Rationale**	3	Describe the rationale for the review in the context of what is already known*, including mention of why a network meta-analysis has been conducted.*	3-4
**Objectives**	4	Provide an explicit statement of questions being addressed, with reference to participants, interventions, comparisons, outcomes, and study design (PICOS).	4-
METHODS			
**Protocol and registration**	5	Indicate whether a review protocol exists and if and where it can be accessed (e.g., Web address); and, if available, provide registration information, including registration number.	5 PROSPERO (CRD42021291003)
**Eligibility criteria**	6	Specify study characteristics (e.g., PICOS, length of follow-up) and report characteristics (e.g., years considered, language, publication status) used as criteria for eligibility, giving rationale. *Clearly describe eligible treatments included in the treatment network, and note whether any have been clustered or merged into the same node (with justification)*	5-6
**Information sources**	7	Describe all information sources (e.g., databases with dates of coverage, contact with study authors to identify additional studies) in the search and date last searched.	5
**Search**	8	Present full electronic search strategy for at least one database, including any limits used, such that it could be repeated.	Appendix 3

**Study selection**	9	State the process for selecting studies (i.e., screening, eligibility, included in systematic review, and, if applicable, included in the meta-analysis).	5<br>Appendix 3
**Data collection process**	10	Describe method of data extraction from reports (e.g., piloted forms, independently, in duplicate) and any processes for obtaining and confirming data from investigators.	6-7
**Data items**	11	List and define all variables for which data were sought (e.g., PICOS, funding sources) and any assumptions and simplifications made	Appendix 2
**Risk of bias within individual studies**	12	Describe methods used for assessing risk of bias of individual studies (including specification of whether this was done at the study or outcome level), and how this information is to be used in any data synthesis.	7
**Summary measures**	13	State the principal summary measures (e.g. difference in means). *Also describe the use of additional summary measures assessed, such as the assessment of Inconsistency*	7-8
**Planned methods of analysis**	14	Describe the methods of handling data and combining results of studies for each network meta-analysis. This should include, but not be limited to:<br>● *Handling of multi-arm trials;*<br>● *Selection of variance structure;*<br>● *Selection of prior distributions in Bayesian analyses;*<br>*and*	7-8

		● *Assessment of model fit.*	
**Risk of bias across studies**	15	Specify any assessment of risk of bias that may affect the cumulative evidence (e.g., publication bias, selective reporting within studies).	7-8
**Additional analyses**	16	Describe methods of additional analyses if done, indicating which were pre-specified. This may include, but not be limited to, the following:<br>● Subgroup analyses;<br>● Meta-regression analyses;<br>● Egger's test.	7-8
RESULTS†			
**Study selection**	17	Give numbers of studies screened, assessed for eligibility, and included in the review, with reasons for exclusions at each stage, ideally with a flow diagram.	9<br>Figure 1
**Presentation of network structure**	**S1**	Provide a network graph of the included studies to enable visualization of the geometry of the treatment network.	Figure 3
**Summary of network geometry**	**S2**	Provide a brief overview of characteristics of the treatment network. This may include commentary on the abundance of trials and randomized patients for the different interventions and pairwise comparisons in the network, gaps of evidence in the treatment network, and potential biases reflected by the network structure.	Table 2-5<br>Appendix 7
**Study characteristics**	18	For each study, present characteristics for which data were extracted (e.g., study size, PICOS, follow-up period) and provide the citations.	Table 1

**Risk of bias within studies**	19	Present data on risk of bias of each study and, if available, any outcome level assessment.	Figure 2<br>Appendix 6
**Results of individual studies**	20	For all outcomes considered (benefits or harms), present, for each study: 1) simple summary data for each intervention group, and 2) effect estimates and confidence intervals. *Modified approaches may be needed to deal with information from larger networks.*	Figure 2<br>Figure 3<br>Appendix 7
**Synthesis of results**	21	Present results of each meta-analysis done, including confidence/credible intervals. *In larger networks, authors may focus on comparisons versus a particular comparator (e.g. placebo or standard care), with full findings presented in an appendix. League tables and forest plots may be considered to summarize pairwise comparisons.* If additional summary measures were explored (such as treatment rankings), these should also be presented	9-11,<br>Table 2-5
**Exploration for inconsistency**	**S3**	Describe results from investigations of inconsistency. This may include such information as measures of model fit to compare consistency and inconsistency models, *P* values from statistical tests, or summary of inconsistency estimates from different parts of the treatment network.	Appendix 9
**Risk of bias across studies**	22	Present results of any assessment of risk of bias across studies for the evidence base being studied.	Figure 2<br>Appendix 6
**Results of additional**	23	Give results of additional analyses, if done (e.g., subgroup analyses, meta-regression analyses,	11<br>Table 7-8

**analyses**		Egger's test).	Appendix 10, 11
DISCUSSION			
**Summary of evidence**	24	Summarize the main findings, including the strength of evidence for each main outcome; consider their relevance to key groups (e.g., healthcare providers, users, and policy-makers).	13
**Limitations**	25	Discuss limitations at study and outcome level (e.g., risk of bias), and at review level (e.g., incomplete retrieval of identified research, reporting bias). *Comment on the validity of the assumptions, such as transitivity and consistency. Comment on any concerns regarding network geometry (e.g., avoidance of certain comparisons).*	15-16
**Conclusions**	26	Provide a general interpretation of the results in the context of other evidence, and implications for future research.	17
FUNDING			
**Funding**	27	Describe sources of funding for the systematic review and other support (e.g., supply of data); role of funders for the systematic review. This should also include information regarding whether funding has been received from manufacturers of treatments in the network and/or whether some of the authors are content experts with professional conflicts of interest that could affect use of treatments in the network.	19

PICOS = population, intervention, comparators, outcomes, study design.

Appendix 2: Protocol

Review title and timescale

1. Review title.

Give the title of the review in English

What exercise priscription is optimal to improve glycemic regulation and body composition in prediabetes: a network meta-analysis and review

2. Original language title.

For reviews in languages other than English, give the title in the original language. This will be displayed with the English language title.

3. Anticipated or actual start date.

Give the date the systematic review started or is expected to start.

14/10/2021

4. Anticipated completion date.

Give the date by which the review is expected to be completed.

10/04/2022

5. Stage of review at time of this submission.

This field uses answers to initial screening questions. It cannot be edited until after registration.

Tick the boxes to show which review tasks have been started and which have been completed.

Update this field each time any amendments are made to a published record.

The review has not yet started:

Review stage	Started	Completed
Preliminary searches	Yes	No
Piloting of the study selection process	Yes	No
Formal screening of search results against eligibility criteria	No	No
Data extraction	No	No
Risk of bias (quality) assessment	No	No
Data analysis	No	No

Provide any other relevant information about the stage of the review here

6. Named contact.

The named contact is the guarantor for the accuracy of the information in the register record. This may be any member of the review team.

Hang Zhang

Email salutation (e.g. "Dr Smith" or "Joanne") for correspondence:

Dr Yang

7. Named contact email.

Give the electronic email address of the named contact.

8. Named contact address

Give the full institutional/organisational postal address for the named contact.

9. Named contact phone number.

Give the telephone number for the named contact, including international dialling code.

10. Organisational affiliation of the review.

Full title of the organisational affiliations for this review and website address if available. This field may be completed as 'None' if the review is not affiliated to any organisation.

Organisation web address:

None

11. Review team members and their organisational affiliations.

Give the personal details and the organisational affiliations of each member of the review team. Affiliation refers to groups or organisations to which review team members belong. **NOTE: email and country now MUST be entered for each person, unless you are amending a published record.**

12. Funding sources/sponsors.

Details of the individuals, organizations, groups, companies or other legal entities who have funded or sponsored the review.

None

Grant number(s)

None

13. Conflicts of interest.

List actual or perceived conflicts of interest (financial or academic).

None

14. Collaborators.

Give the name and affiliation of any individuals or organisations who are working on the review but who are not listed as review team members. NOTE: email and country must be completed for each person, unless you are amending a published record.

15. Review question.

State the review question(s) clearly and precisely. It may be appropriate to break very broad questions down into a series of related more specific questions. Questions may be framed or refined using PI(E)COS or similar where relevant.

1)To assess the comparative efficacy of different exercise types (aerobic, resistance training, or combined) on prediabetes (FBG、2h-glucose、BMI in kg/m squared、 Weight), and other metabolic markers（TC、SBP、DBP.etc） in prediabetes.

2)To determine the optimal exercise prescription (frequency, intensity, time and type (mode)) to improve metabolic health in prediabetes.

3)To establish a hierarchy of exercise interventions (aerobic, resistance training or combined) for treating insulin resistance and improving metabolic health in prediabetes.

16. Searches.

State the sources that will be searched (e.g. Medline). Give the search dates, and any restrictions (e.g. language or publication date). Do NOT enter the full search strategy (it may be provided as a link or attachment below.)

The systematic review and meta-analysis were conducted in accordance with the Preferred Reporting Items for Systematic Reviews and Meta-Analyses (PRISMA) guidelines. We will search PubMed, EMBASE, PsycINFO, The Cochrane Library (Cochrane Database of Systematic Reviews, Cochrane Central Register of Controlled Trials (CENTRAL), Cochrane Methodology Register), and Web of Science.

There will be no language restrictions. Studies published between inception and the date the searches are run will be sought. The searches will be re-run just before the final analyses and further studies retrieved for inclusion. We will use a Boolean search strategy with the operators AND, OR, NOT, and the search strategy will include terms describing or relating to intervention, participants, and study design.

17. URL to search strategy.

Upload a file with your search strategy, or an example of a search strategy for a specific database, (including the keywords) in pdf or word format. In doing so you are consenting to the file being made publicly accessible. Or provide a URL or link to the strategy. Do NOT provide links to your search results.

I give permission for this file to be made publicly available

Yes

18. Condition or domain being studied.

Give a short description of the disease, condition or healthcare domain being studied in your systematic review.

Prediabetes and exercise

19. Participants/population.

Specify the participants or populations being studied in the review. The preferred format includes details of both inclusion and exclusion criteria.

We will include people aged ⩾18 years diagnosed with prediabetes or at high risk for diabetes (non-gestational diabetes)

20. Intervention(s), exposure(s).

Give full and clear descriptions or definitions of the interventions or the exposures to be reviewed. The preferred format includes details of both inclusion and exclusion criteria.

Exercise: aerobic, resistance or combined (aerobic + resistance) and its parameters: frequency, intensity,time and type (mode).

1)We will focus on the following six exercise training modes: moderate intensity AT, high intensity AT, low-moderate load RT, high load RT， combined moderate AT and low-moderate load RT， and vigorous AT combined with high load RT.AT for aerobic exercise, RT for resistance exercise.

21. Comparator(s)/control.

Where relevant, give details of the alternatives against which the intervention/exposure will be compared (e.g. another intervention or a non-exposed control group). The preferred format includes details of both inclusion and exclusion criteria.

One of the following seven exercise training patterns: moderate AT, vigorous AT, low-moderate load RT, High load RT, combined moderate AT with low-moderate load RT, combined vigorous AT and high load RT or no exercise. AT stands for aerobic training and RT stands for resistance exercise.

22. Types of study to be included.

Give details of the study designs (e.g. RCT) that are eligible for inclusion in the review. The preferred format includes both inclusion and exclusion criteria. If there are no restrictions on the types of study, this should be stated.

RCTs

23. Context.

Give summary details of the setting or other relevant characteristics, which help define the inclusion or exclusion criteria.

24. Main outcome(s).

Give the pre-specified main (most important) outcomes of the review, including details of how the outcome is defined and measured and when these measurements are made, if these are part of the review inclusion criteria.

glycated haemoglobin(HbA1c), fasting and after meals 2-hour glucose and insulin resistance.

Measures of effect

Please specify the effect measure(s) for your main outcome(s) e.g. relative risks, odds ratios, risk difference, and/or 'number needed to treat.

Change outcome difference in outcomes upon cessation of exercise lasting at least 6 weeks.

25. Additional outcome(s).

List the pre-specified additional outcomes of the review, with a similar level of detail to that required for main outcomes. Where there are no additional outcomes please state 'None' or 'Not applicable' as appropriate to the review.

1.- Metabolic measures: resting systolic and diastolic blood pressure, total cholesterol, high-density lipoprotein cholesterol, ratio of total cholesterol to high-density lipoprotein cholesterol, non-high density lipoprotein cholesterol, low-density lipoprotein cholesterol, triglycerides.

2.-Anthropometry measures: body weight, body mass index.

Measures of effect

Change outcome difference in outcomes upon cessation of exercise lasting at least 6 weeks.

26. Data extraction (selection and coding).

Describe how studies will be selected for inclusion. State what data will be extracted or obtained. State how this will be done and recorded.

Two authors will independently extract data. Any disagreement will be resolved by discussion until consensus is reached or by consulting a third author

27. Risk of bias (quality) assessment.

State which characteristics of the studies will be assessed and/or any formal risk of bias/quality assessment tools that will be used.

The risk of bias of all included RCTs will be assessed according to the Cochrane Handbook version 5.1.0 17 including method of adequate sequence generation (selection bias), allocation concealment (selection bias),blinding of participants and personnel (performance bias and detection bias), incomplete outcome data(attrition bias), selective reporting (reporting bias), and other bias. We will evaluate methodological quality as low, high, or unclear risk of bias. The risk of bias assessment will be completed by two reviewers, and conflicts will be resolved by a third reviewer.

28. Strategy for data synthesis.

Describe the methods you plan to use to synthesise data. This must not be generic text but should be specific to your review and describe how the proposed approach will be applied to your data. If meta-analysis is planned, describe the models to be used, methods to explore statistical heterogeneity, and software package to be used.

We will use STATA V.14.0 software (Stata Corporation, College Station, Texas, USA) to conduct pairwise meta-analysis. The pooled odds ratios (ORs) with 95% confidence interval (95%CI) will be used to present dichotomous outcomes, and weight mean differences (WMDs) or standard mean differences (SMDs) with 95% CI for continue outcomes. Heterogeneity of treatment effects across trials will be assessed by I^2 statistics and the χ^2 statistics. The Inverse Variance fixed effects model will be used for metaanalysis if the p value is =0.1, and I^2 =50%, which means there was no statistical heterogeneity, or the I-V heterogeneity random effects model will be used to perform meta-analysis. We will use package netmeta version 0.9-2 of R-3.4.0 software to perform a Frequentist network

metaanalysis. The function of decomp.design will be performed to assess the homogeneity in the whole network, the homogeneity within designs, and the homogeneity/consistency between designs. If a loop connecting three arms existed, node splitting method will be used to evaluate inconsistency between direct and indirect comparisons. Treatment ranking will be performed by P-scores, which are based solely on the point estimates and standard errors of the network estimates. They measure the extent of certainty that a treatment is better than another treatment, averaged over all competing treatments

29. Analysis of subgroups or subsets.

State any planned investigation of 'subgroups'. Be clear and specific about which type of study or participant will be included in each group or covariate investigated. State the planned analytic approach.

Given possible significant heterogeneity or inconsistencies, we will use a subgroup analysis to explore possible sources.Subgroup analysis was performed by age, sex, frequency and duration of exercise.We will also assess the sensitivity of the main results by analyzing studies that include patients without complications.

30. Type and method of review.

Select the type of review, review method and health area from the lists below.

Type of review

Network meta-analysis

31. Language.

Select each language individually to add it to the list below, use the bin icon to remove any added in error.

English

32. Country.

Select the country in which the review is being carried out. For multi-national collaborations select all the countries involved.

China

33. Other registration details.

Name any other organisation where the systematic review title or protocol is registered (e.g. Campbell, or The Joanna Briggs Institute) together with any unique identification number assigned by them. If extracted data will be stored and made available through a repository such as the Systematic Review Data Repository (SRDR), details and a link should be included here. If none, leave blank.

34. Reference and/or URL for published protocol.

If the protocol for this review is published provide details (authors, title and journal details, preferably in Vancouver format) Add web link to the published protocol. Or, upload your published protocol here in pdf format. Note that the upload will be publicly accessible.

No I do not make this file publicly available until the review is complete

Please note that the information required in the PROSPERO registration form must be completed in full even if access to a protocol is given.

35. Dissemination plans.

Do you intend to publish the review on completion?

Yes

36. Keywords.

Give words or phrases that best describe the review. Separate keywords with a semicolon or new line. Keywords help PROSPERO users find your review (keywords do not appear in the public record but are included in searches). Be as specific and precise as possible. Avoid acronyms and abbreviations unless these are in wide use.

37. Details of any existing review of the same topic by the same authors.

If you are registering an update of an existing review give details of the earlier versions and include a full bibliographic reference, if available.

38. Current review status.

Update review status when the review is completed and when it is published. New registrations must be ongoing so this field is not editable for initial submission. Please provide anticipated publication date

Review Ongoing

39. Any additional information.

Provide any other information relevant to the registration of this review.

This study is only part of a randomized controlled experiment. Through this study, we want to find the optimal exercise prescription to improve the ability of daily living of Prediabetes, and use this as the experimental group of our randomized controlled trial, and compare different exercise doses to Prediabetes the impact of daily living.

40. D./ .l etails of final report/publication(s) or preprints if available.

Leave empty until publication details are available OR you have a link to a preprint (NOTE: this field is not editable for initial submission).

List authors, title and journal details preferably in Vancouver format.

Give the link to the published review or preprint.

Appendix 3: Search Strategy

3.1 Database: PubMed <inception to October 10 2022>

Search Strategy:

#21	Search: ((Prediabetic State [MeSH Terms]) AND ((((((((((exercise* [MeSH Terms]) OR (resistance training [MeSH Terms])) OR (High-Intensity Interval Training [MeSH Terms])) OR (Circuit-Based Exercise [MeSH Terms])) OR (Tai ji [MeSH Terms])) OR ("aerobic exercise" or "aerobic training" or "Flexibility training" or "multicomponent exercise program" or "multidisciplinary exercise program" or "Nordic Walking" or "Physiotherapy" or "pilates" or "power training" or "treadmill training" or "walking" or "running"))) AND ((((((((randomized controlled trial [Publication Type]) OR (controlled clinical trial[Publication Type])) OR (randomized [Title/Abstract])) OR (placebo [Title/Abstract])) OR (randomly [Title/Abstract])) OR (trial [Title])) OR (clinical trials as topic [MeSH Terms])) NOT ((animals [MeSH Terms]) NOT (humans [MeSH Terms])))	1949
#20	Search: (((((((randomized controlled trial [Publication Type]) OR (controlled clinical trial [Publication Type])) OR (randomized [Title/Abstract])) OR (placebo [Title/Abstract])) OR (randomly [Title/Abstract])) OR (trial [Title])) OR (clinical trials as topic [MeSH Terms])) NOT ((animals [MeSH Terms]) NOT (humans [MeSH Terms]))	1,460,057

#19	Search: ((((((randomized controlled trial [Publication Type]) OR (controlled clinical trial [Publication Type])) OR (randomized [Title/Abstract])) OR (placebo [Title/Abstract])) OR (randomly [Title/Abstract])) OR (trial [Title])) OR (clinical trials as topic [MeSH Terms])	1,799,515
#18	Search: ((((((((((exercise*[MeSH Terms]) OR (resistance training [MeSH Terms])) OR (High-Intensity Interval Training [MeSH Terms])) OR (Circuit-Based Exercise [MeSH Terms])) OR (Tai ji [MeSH Terms])) OR ("aerobic exercise" or "aerobic training" or "Flexibility training" or "multicomponent exercise program" or "multidisciplinary exercise program" or "Nordic Walking" or "Physiotherapy" or "pilates" or "power training" or "treadmill training" or "walking" or "running")	827,768
#17	Search: (animals [MeSH Terms]) NOT (humans [MeSH Terms])	5,063,230
#16	Search: humans [MeSH Terms]	20,861,338
#15	Search: animals [MeSH Terms]	25,924,568
#14	Search: clinical trials as topic [MeSH Terms]	378,145
#13	Search: trial [Title]	273,588
#12	Search: randomly [Title/Abstract]	396,364
#11	Search: placebo [Title/Abstract]	239,927
#10	Search: randomized [Title/Abstract]	634,720
#9	Search: controlled clinical trial [Publication Type]	672,064
#8	Search: randomized controlled trial [Publication Type]	581,868
#7	Search: "aerobic exercise" or "aerobic training" or "Flexibility training" or	242,542

	"multicomponent exercise program" or "multidisciplinary exercise program" or "Nordic Walking" or "Physiotherapy" or "pilates" or "power training" or "treadmill training" or "walking" or "running"	
#6	Search: Tai ji [MeSH Terms]	1,394
#5	Search: Circuit-Based Exercise [MeSH Terms]	107
#4	Search: High-Intensity Interval Training [MeSH Terms]	1954
#3	Search: resistance training [MeSH Terms]	11,709
#2	Search: exercise* [MeSH Terms]	237,893
#1	Search: Prediabetic State [MeSH Terms]	8649

3.2 Database: Embase <inception to October 10 2022>

Search Strategy:

1 Prediabetic state.mp. (655110)

2 exp Prediabetic state / (596811)

3 (aerobic exercise or aerobic training or Flexibility training or exercise$ or resistance training or High-Intensity Interval Training or Circuit-Based Exercise or "multidisciplinary exercise program" or "Nordic Walking" or "Physiotherapy" or "pilates" or "power training" or "treadmill training" or "walking" or "running").mp. (693118)

4 exp resistance training/ (23552)

5 exp exercise$/ (403332)

6 exp High-Intensity Interval Training / (3578)

7 exp Circuit-Based Exercise / (309)

8 exp Tai ji / (234)

9 randomized.ab. (810118)

10 randomly.ab. (505005)

11 trial.ti. (363772)

12 exp clinical trial/ (1709346)

13 exp randomized controlled trials/ (225556)

14 exp cross-over studies/ (70232)

15 (clinic$ adj2 trial).mp. (2194442)

16 (random$ adj5 control$ adj5 trial$).mp. (1135615)

17 (crossover or cross-over).mp. (129200)

18 randomi$.mp. (1413386)

19 (random$ adj5 (assign$ or allocat$ or assort$ or reciev$)).mp. (285535)

20 1 or 2 (655110)

21 3 or 4 or 5 or 6 or 7 or 8 (454079)

22 9 or 10 or 11or 12 or 13 or 14 or or 16 or 17 or 18 or 19 (3076021)

23 20 and 21 and 22 (1323)

3.3 Cochrane

#1 MeSH descriptor: [Prediabetic State] explode all trees (10390)

#2 ("aerobic exercise" or "aerobic training" or "Flexibility training" or "multidisciplinary exercise program" or "Nordic Walking" or "Physiotherapy" or "pilates" or "power training" or "treadmill training" or "walking" or "running") in Trials (Word variations have been searched) (9291)

#3 MeSH descriptor: [resistance training] explode all trees (4108)

#4 MeSH descriptor: [exercise] explode all trees (28159)

#5 MeSH descriptor: [High-Intensity Interval Training] explode all trees (651)

#6 MeSH descriptor: [Circuit-Based Exercise] explode all trees (39)

#7 MeSH descriptor: [Tai ji] explode all trees (57)

#8 #2 or #3 or #4 or #5 or #6 or #7 (36821)

#9 #1 and #8 (353)

3.4 Database: Web of Science <inception to October 10 2022>

#10	885	#9 AND #8 AND #1
		Indexes=SCI-EXPANDED, SSCI, A&HCI, CPCI-S, CPCI-SSH, BKCI-S, BKCI-SSH, ESCI, CCR-EXPANDED, IC Timespan=All years
#9	923790	#7 OR #6 OR #5 OR #4 OR #3 OR #2
		Indexes=SCI-EXPANDED, SSCI, A&HCI, CPCI-S, CPCI-SSH, BKCI-S, BKCI-SSH, ESCI, CCR-EXPANDED, IC Timespan=All years
#8	14265459	TOPIC: (("randomized controlled trial*" or "controlled clinical trial" or "random*" or "clinical trial*" or "randomly" or "trial" or "clinical trial" or "randomized controlled trial*" or "cross-over studies" or "clinic*"))
		Indexes=SCI-EXPANDED, SSCI, A&HCI, CPCI-S, CPCI-SSH, BKCI-S, BKCI-SSH, ESCI, CCR-EXPANDED, IC Timespan=All years
#7	728	TOPIC: ("Circuit Based Exercise" or "Circuit-Based Exercises" or "Exercise,

		Circuit-Based" or "Exercises, Circuit-Based" or "Circuit Training" or "Training, Circuit")
		Indexes=SCI-EXPANDED, SSCI, A&HCI, CPCI-S, CPCI-SSH, BKCI-S, BKCI-SSH, ESCI, CCR-EXPANDED, IC Timespan=All years
#6	4993	TOPIC: ("High Intensity Interval Training" or "High-Intensity Interval Trainings" or "Interval Training, High-Intensity" or "Training, High-Intensity Interval" or "Trainings, High-Intensity Interval" or "High-Intensity Intermittent Exercise" or "Exercise, High-Intensity Intermittent" or "Exercises, High-Intensity Intermittent" or "High-Intensity Intermittent Exercises" or "Sprint Interval Training" or "Sprint Interval Trainings")
		Indexes=SCI-EXPANDED, SSCI, A&HCI, CPCI-S, CPCI-SSH, BKCI-S, BKCI-SSH, ESCI, CCR-EXPANDED, IC Timespan=All years
#5	917927	TOPIC: ("Exercise*" or "Exercise Program, Weight-Bearing" or "Exercise Programs, Weight-Bearing" or "Weight Bearing Exercise Program” or "Weight-Bearing Exercise Programs" or "Physical Activity" or "Activities, Physical" or "Activity, Physical" or "Physical Activities" or "Exercise, Physical" or "Exercises, Physical" or "Physical Exercise" or "Physical Exercises" or "Exercise, Isometric" or "Exercises, Isometric" or "Isometric Exercises" or "Isometric Exercise" or "Exercise, Aerobic" or "Aerobic Exercise" or "Aerobic Exercises" or "Exercises, Aerobic" or "Exercise Training" or "Exercise Trainings" or "Training, Exercise" or "Trainings, Exercise")

		Indexes=SCI-EXPANDED, SSCI, A&HCI, CPCI-S, CPCI-SSH, BKCI-S, BKCI-SSH, ESCI, CCR-EXPANDED, IC Timespan=All years
#4	27435	TOPIC: ("Resistance training" or "Training, Resistance" or "Strength Training" or "Training, Strength" or "Weight-Lifting Strengthening Program" or "Strengthening Program, Weight-Lifting" or "Strengthening Programs, Weight-Lifting" or "Weight Lifting Strengthening Program" or "Weight Lifting Strengthening Programs" or "Weight-Lifting Exercise Program" or "Exercise Program, Weight-Lifting" or "Exercise Programs, Weight-Lifting" or "Weight Lifting Exercise Program" or "Weight-Lifting Exercise Programs" or "Weight-Bearing Strengthening Program" or "Strengthening Program, Weight-Bearing" or "Strengthening Programs, Weight-Bearing" or "Weight Bearing Strengthening Program" or "Weight-Bearing Strengthening Programs" or "Weight-Bearing Exercise Program")
		Indexes=SCI-EXPANDED, SSCI, A&HCI, CPCI-S, CPCI-SSH, BKCI-S, BKCI-SSH, ESCI, CCR-EXPANDED, IC Timespan=All years
#3	11235	TOPIC: ("Tai ji" or "Tai Chi" or "Chi, Tai" or "Tai Ji Quan" or "Ji Quan, Tai" or "Quan, Tai Ji" or "Taiji" or "Taijiquan" or "T'ai Chi" or "Tai Chi Chuan")
		Indexes=SCI-EXPANDED, SSCI, A&HCI, CPCI-S, CPCI-SSH, BKCI-S, BKCI-SSH, ESCI, CCR-EXPANDED, IC Timespan=All years
#2	28292	TOPIC: ("aerobic exercise" or "aerobic training" or "Flexibility training" or

		"multidisciplinary exercise program" or "Nordic Walking" or "Physiotherapy" or "pilates" or "power training" or "treadmill training" or "walking" or "running")
		Indexes=SCI-EXPANDED, SSCI, A&HCI, CPCI-S, CPCI-SSH, BKCI-S, BKCI-SSH, ESCI, CCR-EXPANDED, IC Timespan=All years
#1	173722	TOPIC: ("Prediabetic state" or "Prediabetes" or "States, Prediabetic" or "State, Prediabetic")
		Indexes=SCI-EXPANDED, SSCI, A&HCI, CPCI-S, CPCI-SSH, BKCI-S, BKCI-SSH, ESCI, CCR-EXPANDED, IC Timespan=All years

3.5 Database: Sport Discus <inception to October 10 2022>

Search Strategy:

S1	TX "Prediabetic State"	Search modes- Boolean/Phrase	2761
S2	TX "exercise*"	Search modes- Boolean/Phrase	1469029
S3	TX "aerobic exercise" or "aerobic training" or "Flexibility training" or "multidisciplinary exercise program" or "Nordic Walking" or "Physiotherapy" or "pilates" or "power training" or "treadmill training" or "walking" or "running"	Search modes- Boolean/Phrase	73597
S4	TX "resistance training"	Search modes- Boolean/Phrase	48528
S5	TX "High-Intensity Interval Training"	Search modes- Boolean/Phrase	8957
S6	TX "Circuit-Based Exercise"	Search modes- Boolean/Phrase	83

S7	TX "physical active"	Search modes- Boolean/Phrase	27964
S8	TX "Tai ji"	Search modes- Boolean/Phrase	4366
S9	S2 OR S3 OR S4 OR S5 OR S6 OR S7 OR S8	Search modes- Boolean/Phrase	1490425
S10	AB "randomized"	Search modes- Boolean/Phrase	332636
S11	AB "randomly"	Search modes- Boolean/Phrase	263807
S12	TI "trial"	Search modes- Boolean/Phrase	235254
S13	AB "clinical trial"	Search modes- Boolean/Phrase	210532
S14	AB "randomized controlled trials"	Search modes- Boolean/Phrase	110186
S15	AB "cross-over studies"	Search modes- Boolean/Phrase	13464
S16	AB "randomi* "	Search modes- Boolean/Phrase	409640
S17	S10 OR S11 OR S12 OR S13 OR S14 OR S15 OR S16	Search modes- Boolean/Phrase	874166
S18	SU "animals "	Search modes- Boolean/Phrase	508431
S19	S1 AND S9	Search modes- Boolean/Phrase	460
S20	S17 AND S19	Search modes- Boolean/Phrase	48
S22	S20 NOT S18	Search modes- Boolean/Phrase	43

Appendix 4: Definitions of physical activity types and non-exercise training control

Type of exercise	Abbreviation	Definition
Aerobic:vigorous intensity	AT-V	**Frequency**:3~5 times/week,each session lasting 30~60min<br>**Intensity**:>65%VO2max or >65%HRR or >75% HRmax<br>**Time**:⩾8 weeks<br>**Type**:Any mode of aerobic only(eg:walking,running,cycling and swimming)
Aerobic:moderate intensity	AT-M	**Frequency**:3~5 times/week,each session lasting 30~60min<br>**Intensity**:45%~65%VO2max or 50%~65%HRR or 65%~75% HRmax<br>**Time**:⩾8 weeks<br>**Type**:Any mode of aerobic only(eg:walking,running,cycling and swimming)
Resistance:high intensity	RT-H	**Frequency**:3~5 times/week,each session lasting 30~60min<br>**Intensity**:Average maximum load>75%1RM<br>**Time**:⩾8 weeks<br>**Type**:Any mode of resistance training(eg:free weights,weights machines and resistance bands)

Resistance:low~moderate load	RT-L	**Frequency**:3~5 times/week,each session lasting 30~60min<br>**Intensity**:Average maximum load 50%~75%1RM<br>**Time**:⩾8 weeks<br>**Type**:Any mode of resistance training(eg:free weights,weights machines and resistance bands)
Combined:high intensity	AT-V+RT-H	Combined vigorous intensity aerobic exercise with high load resistance training
Combined:low~moderate intensity	AT-M+RT-L	Combined moderate intensity aerobic exercise with low~moderate load resistance training
Control	CON	No exercise

Reference

1. U.S. Department of Health and Human Services (2018) Physical Activity Guidelines for Americans, 2nd edition. https://health.gov/paguidelines/second-edition/pdf/Physical_Activity_Guidelines_2nd_edition.pdf
2. Gonçalves C, Raimundo A, Abreu A, Bravo J (2021) Exercise Intensity in Patients with Cardiovascular Diseases: Systematic Review with Meta-Analysis. Int J Environ Res Public Health 18(7):3574. https://doi.org/10.3390/ijerph18073574

3. Gary Liguori, American College of Sports Medicine (ACSM) (2020) ACSM's Guidelines for Exercise Testing and Prescription, 11th edition. Wolters Kluwer Health, Indiana

4. Mitchell BL, Lock MJ, Davison K, Parfitt G, Buckley JP, Eston RG (2019) What is the effect of aerobic exercise intensity on cardiorespiratory fitness in those undergoing cardiac rehabilitation? A systematic review with meta-analysis. Br J Sports Med 53(21):1341-1351. https://doi.org/10.1136/bjsports-2018-099153

5. Su L, Fu J, Sun S, Zhao G, Cheng W, Dou C, Quan M (2019) Effects of HIIT and MICT on cardiovascular risk factors in adults with overweight and/or obesity: A meta-analysis. PLoS One 14(1):e0210644. https://doi.org/10.1371/journal.pone.0210644

6. Elliott AD, Rajopadhyaya K, Bentley DJ, Beltrame JF, Aromataris EC (2015) Interval training versus continuous exercise in patients with coronary artery disease: a meta-analysis. Heart Lung Circ 24(2):149-57. https://doi.org/10.1016/j.hlc.2014.09.001

7. Daniels J, Scardina N (1984) Interval training and performance. Sports Med 1(4):327-34. https://doi.org/10.2165/00007256-198401040-00006

8. Powell KE, Paluch AE, Blair SN (2011) Physical activity for health: What kind? How much? How intense? On top of what? Annu Rev Public Health 32:349-65. https://doi.org/10.1146/annurev-publhealth-031210-101151

9. Laoutaris ID, Adamopoulos S, Manginas A, Panagiotakos DB, Kallistratos MS, Doulaptsis C, Kouloubinis A, Voudris V, Pavlides G, Cokkinos DV, Dritsas A (2013) Benefits of combined aerobic/resistance/inspiratory training in patients with chronic heart failure. A complete exercise model? A prospective randomised study. Int J Cardiol 167(5):1967-72. https://doi.org/10.1016/j.ijcard.2012.05.019

Appendix 5: Assessment of the transitivity

Different clinical trials need to ensure that their baseline levels are consistent. If the baseline levels are inconsistent, the results cannot be transitive. Therefore, the transitivity assumption was evaluated by comparing the distribution of potential effect modifiers (publication year, sample size, mean age, percentage male) across studies grouped before analyzing the results, and we use the SPSS to draw boxplots between the above potential influencing factors and various types of exercises.

5.1 Publish years

We checked the publication year distribution of the included studies. The range is from 1998 to 2021.

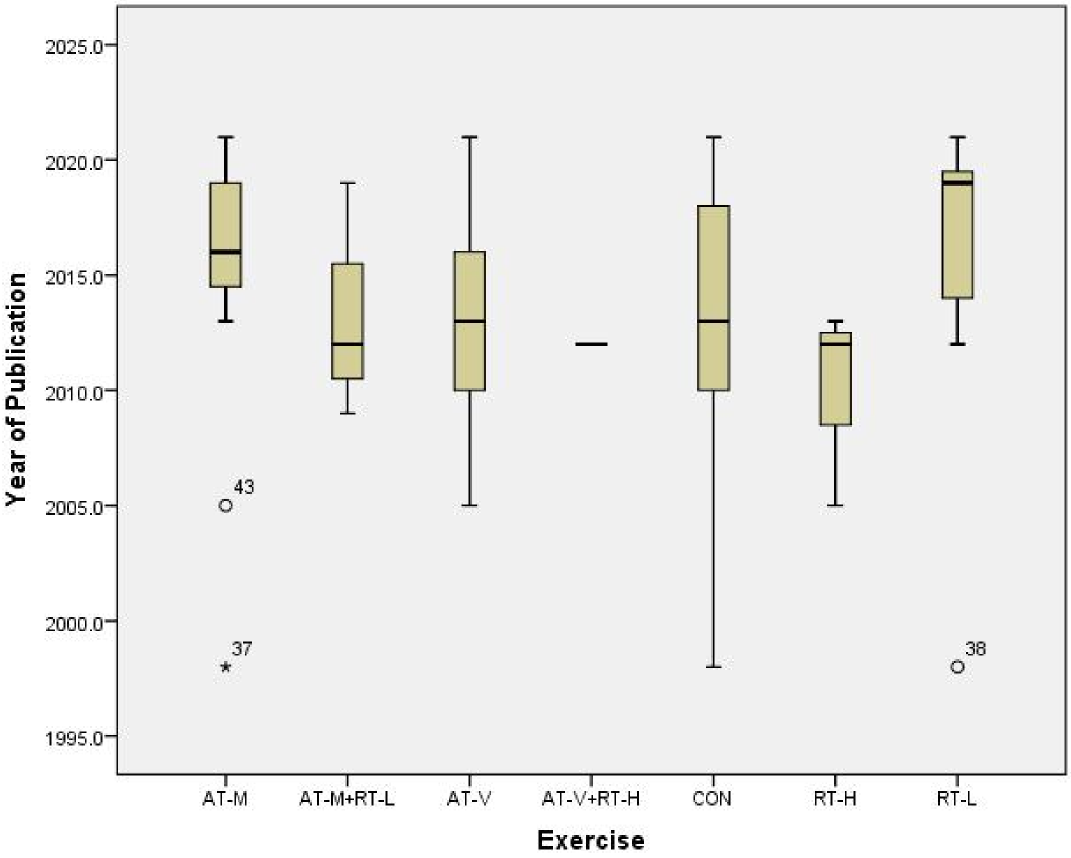

Figure 5.1: Boxplot for distribution of publication year. *RT-L* Resistance training of low-moderate load, *AT-M* Aerobic training of moderate intensity, *AT-M+RT-L* Combined moderate intensity aerobic exercise with low to moderate load resistance training, *AT-V* Aerobic training of vigorous intensity, *AT-V+RT-H* Combined vigorous intensity aerobic exercise with high load resistance training, *RT-H* Resistance training of high load.

5.2 Mean age

We checked the mean age distribution of the included study participants. The range is from 42.4 to 64.23.

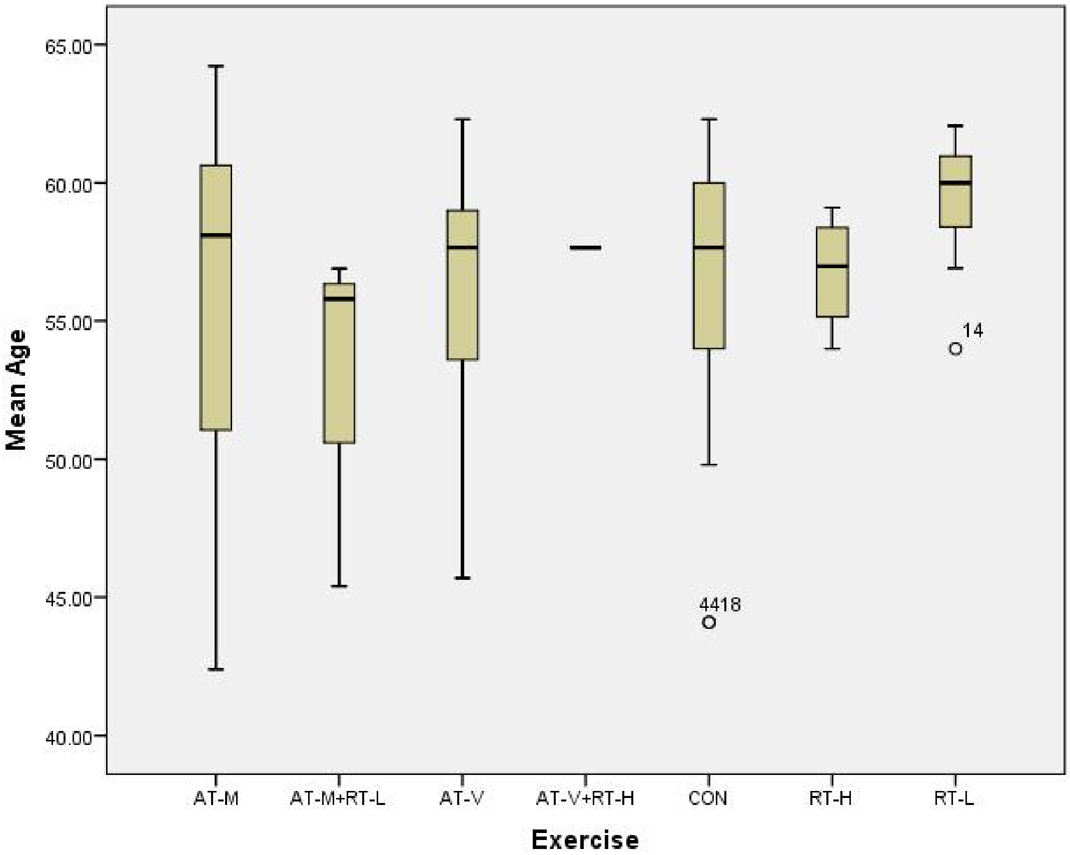

Figure 5.2: Boxplot for distribution of mean age. *RT-L* Resistance training of low-moderate load, *AT-M* Aerobic training of moderate intensity, *AT-M+RT-L* Combined moderate intensity aerobic exercise with low to moderate load resistance training, *AT-V* Aerobic training of vigorous intensity, *AT-V+RT-H* Combined vigorous intensity aerobic exercise with high load resistance training, *RT-H* Resistance training of high load.

5.3 Percentage male

We checked the percentage male distribution of the included study participants. The range is from 0% to 100%.

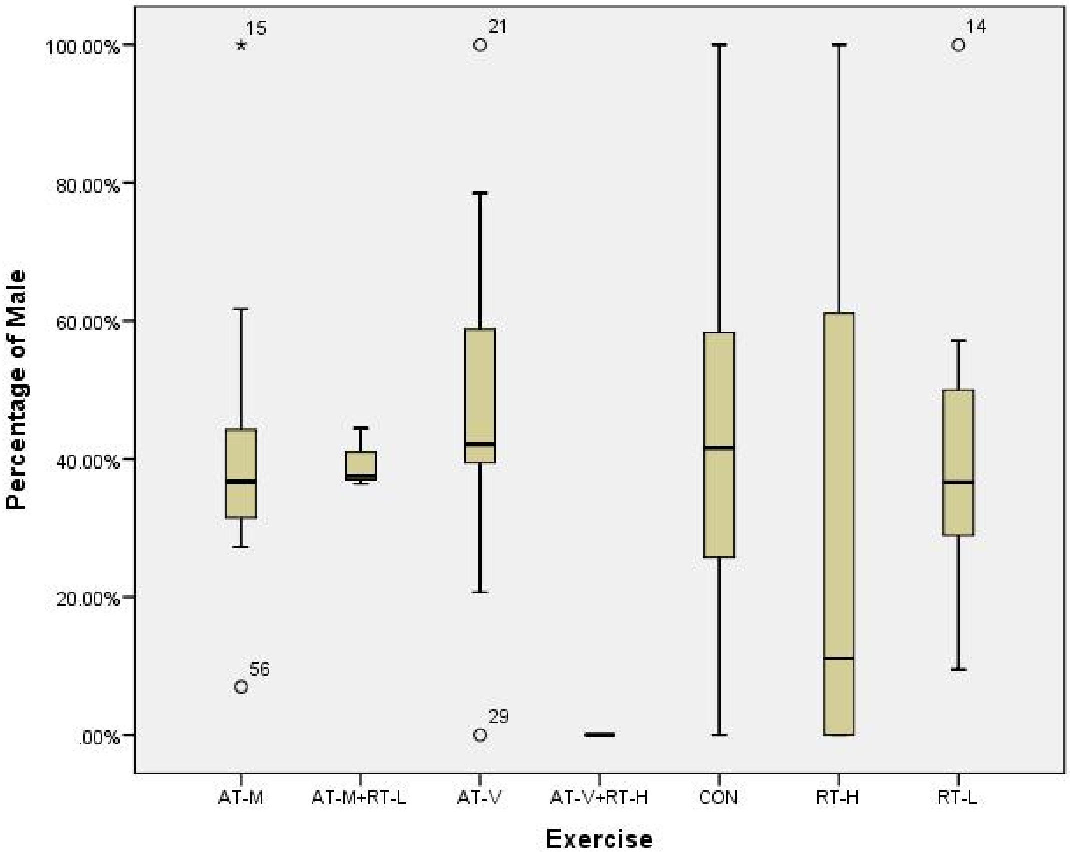

Figure 5.3: Boxplot for distribution of percentage male. *RT-L* Resistance training of low-moderate load, *AT-M* Aerobic training of moderate intensity, *AT-M+RT-L* Combined moderate intensity aerobic exercise with low to moderate load resistance training, *AT-V* Aerobic training of vigorous intensity, *AT-V+RT-H* Combined vigorous intensity aerobic exercise with high load resistance training, *RT-H* Resistance training of high load.

5.4.sample size

We checked the sample size distribution of the included studies. The range is from 6 to 136.

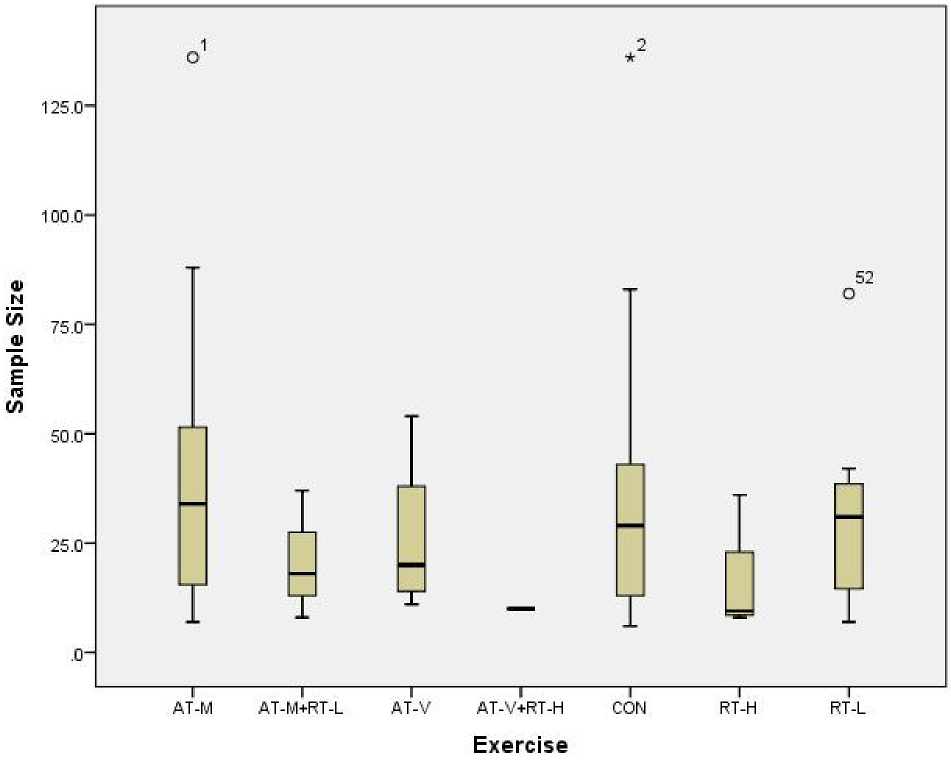

Figure 5.4: Boxplot for distribution of sample size. *RT-L* Resistance training of low-moderate load, *AT-M* Aerobic training of moderate intensity, *AT-M+RT-L* Combined moderate intensity aerobic exercise with low to moderate load resistance training, *AT-V* Aerobic training of vigorous intensity, *AT-V+RT-H* Combined vigorous intensity aerobic exercise with high load resistance training, *RT-H* Resistance training of high load.

Appendix 6: The risk of bias assessment for the individual included studies.

Author	Random sequence generation	Allocation concealment	Blinding of participants and personnel	Blinding of outcome	uncomplete outcome data	Selective reporting (reporting bias)	other bias
Tahereh 2019	?	+	-	+	+	?	+
Yan J 2019	+	?	-	+	+	+	+
Dai X 2019	+	+	-	+	+	?	+
Kramer 2018	+	+	-	+	+	+	+
Slentz 2016	+	+	-	+	+	?	+
Gidlund 2016	?	?	-	+	+	+	+
Liao 2015	?	?	-	+	+	+	+
Herrzig 2014	+	?	-	+	+	+	+
Venojarvi 2013	+	?	-	+	+	+	+
Fritz 2013	+	+	-	+	+	+	?
Hansen 2012	?	?	-	+	+	+	?

Alvarez 2012	+	?	-	+	+	+	?
Burtscher 2009	?	?	-	+	+	+	?
Desch 2010	?	?	-	+	+	+	?
Eriksson 1998	?	+	-	+	+	+	?
Malin 2012	?	+	-	+	+	+	?
Marcell 2005	?	?	-	+	+	+	?
Marcus 2009	?	?	-	+	+	+	?
Roumen 2008	+	?	-	+	+	+	+
Rowan 2017	+	?	-	+	+	+	+
Yuan 2020	+	?	-	+	+	+	+
sulin 2017	+	?	-	+	+	+	+
Lin 2021	+	?	-	+	+	+	+
Nicole 2019	?	?	-	+	+	+	+

Reference

1. Lan NSR, Lam K, Naylor LH, Green DJ, Minaee NS, Dias P, Maiorana AJ (2020) The Impact of Distinct Exercise Training Modalities on Echocardiographic Measurements in Patients with Heart Failure with Reduced Ejection Fraction. J Am Soc Echocardiogr 33(2):148-156.

2. Besnier F, Labrunée M, Richard L, Faggianelli F, Kerros H, Soukarié L, Bousquet M, Garcia JL, Pathak A, Gales C, Guiraud T, Sénard JM (2019) Short-term effects of a 3-week interval training program on heart rate variability in chronic heart failure A randomised controlled trial. Ann Phys Rehabil 62(5):321-328.

3. Adamopoulos S, Schmid JP, Dendale P, Poerschke D, Hansen D, Dritsas A, Kouloubinis A, Alders T, Gkouziouta A, Reyckers I, Vartela V, Plessas N, Doulaptsis C, Saner H, Laoutaris ID (2014) Combined aerobic/inspiratory muscle training vs. aerobic training in patients with chronic heart failure: The Vent-HeFT trial: a European prospective multicentre randomized trial. Eur J Heart Fail 16(5):574-82.

4. Laoutaris ID, Adamopoulos S, Manginas A, Panagiotakos DB, Kallistratos MS, Doulaptsis C, Kouloubinis A, Voudris V, Pavlides G, Cokkinos DV, Dritsas A (2013) Benefits of combined aerobic/resistance/inspiratory training in patients with chronic heart failure. A complete exercise model? A prospective randomised study. Int J Cardiol 167(5):1967-72.

5. Laoutaris ID, Piotrowicz E, Kallistratos MS et al (2021) Combined aerobic/resistance/inspiratory muscle training as the 'optimum' exercise programme for patients with chronic heart failure: ARISTOS-HF randomized clinical trial. Eur J Prev Cardiol 28(15):1626-1635.

6. Hambrecht R, Gielen S, Linke A, Fiehn E, Yu J, Walther C, Schoene N, Schuler G (2000) Effects of exercise training on left ventricular function and peripheral resistance in patients

with chronic heart failure: A randomized trial. JAMA 283(23):3095-101.

7. Malfatto G, Branzi G, Osculati G, Valli P, Cuoccio P, Ciambellotti F, Parati G, Facchini M (2009) Improvement in left ventricular diastolic stiffness induced by physical training in patients with dilated cardiomyopathy. J Card Fail 15(4):327-33.

8. Erbs S, Linke A, Gielen S, Fiehn E, Walther C, Yu J, Adams V, Schuler G, Hambrecht R (2003) rcise training in patients with severe chronic heart failure: impact on left ventricular performance and cardiac size. A retrospective analysis of the Leipzig Heart Failure Training Trial. Eur J Cardiovasc Prev Rehabil 10(5):336-44.

9. Beer M, Wagner D, Myers J, Sandstede J, Köstler H, Hahn D, Neubauer S, Dubach P (2008) Effects of exercise training on myocardial energy metabolism and ventricular function assessed by quantitative phosphorus-31 magnetic resonance spectroscopy and magnetic resonance imaging in dilated cardiomyopathy. J Am Coll Cardiol 51(19):1883-91. https://doi.org/10.1016/j.jacc.2007.09.075

10. Smart NA, Steele M (2012) A comparison of 16 weeks of continuous vs intermittent exercise training in chronic heart failure patients. Congest Heart Fail 18(4):205-11.

11. Klecha A, Kawecka-Jaszcz K, Bacior B, Kubinyi A, Pasowicz M, Klimeczek P, Banyś R (2007) Physical training in patients with chronic heart failure of ischemic origin: effect on exercise capacity and left ventricular remodeling. Eur J Cardiovasc Prev Rehabil 14(1):85-91.

12. Feiereisen P, Delagardelle C, Vaillant M, Lasar Y, Beissel J (2007) Is strength training the more efficient training modality in chronic heart failure? Med Sci Sports Exerc 39(11):1910-7.

13. Belardinelli R, Georgiou D, Cianci G, Purcaro A (1996) Effects of exercise training on left ventricular filling at rest and during exercise in patients with ischemic cardiomyopathy and

severe left ventricular systolic dysfunction. Am Heart J 132(1 Pt 1):61-70.

14. Kiilavuori K, Sovijärvi A, Näveri H, Ikonen T, Leinonen H (1996) Effect of physical training on exercise capacity and gas exchange in patients with chronic heart failure. Chest 110(4):985-91.

15. Belardinelli R, Georgiou D, Cianci G, Purcaro A (1999) Randomized, controlled trial of long-term moderate exercise training in chronic heart failure: effects on functional capacity, quality of life, and clinical outcome. Circulation 99(9):1173-82.

16. Giannuzzi P, Temporelli PL, Corrà U, Tavazzi L; ELVD-CHF Study Group (2003) Antiremodeling effect of long-term exercise training in patients with stable chronic heart failure: results of the Exercise in Left Ventricular Dysfunction and Chronic Heart Failure (ELVD-CHF) Trial. Circulation 108(5):554-9.

17. Roveda F, Middlekauff HR, Rondon MU, Reis SF, Souza M, Nastari L, Barretto AC, Krieger EM, Negrão CE (2003) The effects of exercise training on sympathetic neural activation in advanced heart failure: a randomized controlled trial. J Am Coll Cardiol 42(5):854-60.

18. Sabelis LW, Senden PJ, Fijnheer R, de Groot PG, Huisveld IA, Mosterd WL, Zonderland ML (2004) Endothelial markers in chronic heart failure: training normalizes exercise-induced vWF release. Eur J Clin Invest 34(9):583-9.

19. Klocek M, Kubinyi A, Bacior B, Kawecka-Jaszcz K (2005) Effect of physical training on quality of life and oxygen consumption in patients with congestive heart failure. Int J Cardiol 103(3):323-9.

20. Passino C, Severino S, Poletti R, Piepoli MF, Mammini C, Clerico A, Gabutti A, Nassi G, Emdin M (2006) Aerobic training decreases B-type natriuretic peptide expression and

adrenergic activation in patients with heart failure. J Am Coll Cardiol 47(9):1835-9.

21. Acanfora D, Scicchitano P, Casucci G, Lanzillo B, Capuano N, Furgi G, Acanfora C, Longobardi M, Incalzi RA, Piscosquito G, Ciccone MM (2016) Exercise training effects on elderly and middle-age patients with chronic heart failure after acute decompensation: A randomized, controlled trial. Int J Cardiol 225:313-323.

22. Ricca-Mallada R, Migliaro ER, Silvera G, Chiappella L, Frattini R, Ferrando-Castagnetto F (2017) Functional outcome in chronic heart failure after exercise training: Possible predictive value of heart rate variability. Ann Phys Rehabil Med 60(2):87-94.

23. Ellingsen Ø, Halle M, Conraads V et al (2017) SMARTEX Heart Failure Study (Study of Myocardial Recovery After Exercise Training in Heart Failure) Group. High-Intensity Interval Training in Patients With Heart Failure With Reduced Ejection Fraction. Circulation 135(9):839-849.

24. Höllriegel R, Winzer EB, Linke A, Adams V, Mangner N, Sandri M, Bowen TS, Hambrecht R, Schuler G, Erbs S (2016) Long-Term Exercise Training in Patients With Advanced Chronic Heart Failure: SUSTAINED BENEFITS ON LEFT VENTRICULAR PERFORMANCE AND EXERCISE CAPACITY. J Cardiopulm Rehabil Prev 36(2):117-24.

25. Wisløff U, Støylen A, Loennechen JP, Bruvold M, Rognmo Ø, Haram PM, Tjønna AE, Helgerud J, Slørdahl SA, Lee SJ, Videm V, Bye A, Smith GL, Najjar SM, Ellingsen Ø, Skjaerpe T (2007) Superior cardiovascular effect of aerobic interval training versus moderate continuous training in heart failure patients: a randomized study. Circulation 115(24):3086-94.

Appendix 7: Results of direct,indirect,network meta-analysis.

7.1 Results of network meta-analysis

7.1.1.1 Network plot of FBG

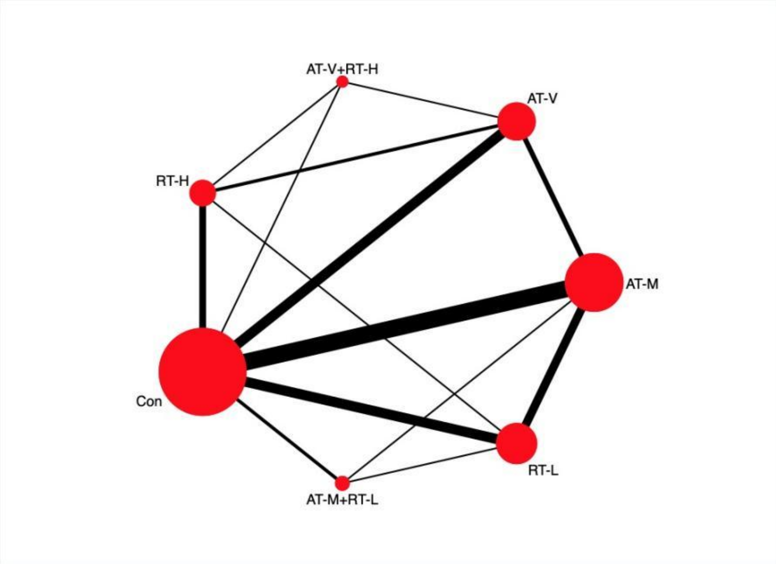

Figure7.1.1 Network plot of FBG.The size of the modes corresponds to the number of participants randomized to each physical activity type.Physical activity type with direct comparisons are linked with a line; Its thickness corresponds to the number of trials evaluating the comparison.RT-L: Resistance training of low-moderate load, AT-M: Aerobic training of moderate intensity, AT-M+RT-L: Combined moderate intensity aerobic exercise with low to moderate load resistance training, AT-V: Aerobic training of vigorous intensity, AT-V+RT-H: Combined vigorous intensity aerobic exercise with high load resistance training, RT-H: Resistance training of high load, Con: No exercise.

7.1.1.2 The league table of FBG

FBG	Comparison of treatments: Mean different (95% confidence intervals) / Effect of intervention in each row compared with intervention in each column					
RT-L	-0.05 (-0.56; 0.46)	-0.08 (-0.27; 0.11)	0.10 (-0.64; 0.84)	NA	NA	-0.52 (-0.71; -0.34)*
-0.04 (-0.31; 0.22)	AT-M+RT-L	-0.01 (-0.50; 0.48)	NA	0.20 (-0.54; 0.94)	NA	-0.50 (-0.79; -0.22)*
-0.10 (-0.27; 0.07)	-0.06 (-0.31; 0.19)	AT-M	NA	-0.11 (-0.43; 0.21)	NA	-0.37 (-0.51; -0.22)*
-0.17 (-0.52; 0.18)	-0.13 (-0.52; 0.26)	-0.07 (-0.41; 0.27)	RT-H	-0.03 (-0.74; 0.68)	-0.16 (-0.79; 0.47)	-0.23 (-0.60; 0.15)
-0.17 (-0.42; 0.07)	-0.13 (-0.42; 0.16)	-0.07 (-0.28; 0.14)	-0.00 (-0.36; 0.36)	AT-V	-0.13 (-0.71; 0.45)	-0.29 (-0.57; -0.01)*
-0.25 (-0.66; 0.15)	-0.21 (-0.65; 0.22)	-0.15 (-0.54; 0.24)	-0.08 (-0.52; 0.35)	-0.08 (-0.47; 0.31)	AT-V+RT-H	-0.30 (-0.83; 0.23)
-0.48 (-0.65; -0.32)*	-0.44 (-0.67; -0.21)*	-0.38 (-0.51; -0.25)*	-0.31 (-0.63; 0.01)	-0.31 (-0.51; -0.11)*	-0.23 (-0.60; 0.14)	CON

Table 7.1.1.2: League Table of FBG.

7.1.2 2hPG

7.1.2.1 Network plot of 2hPG

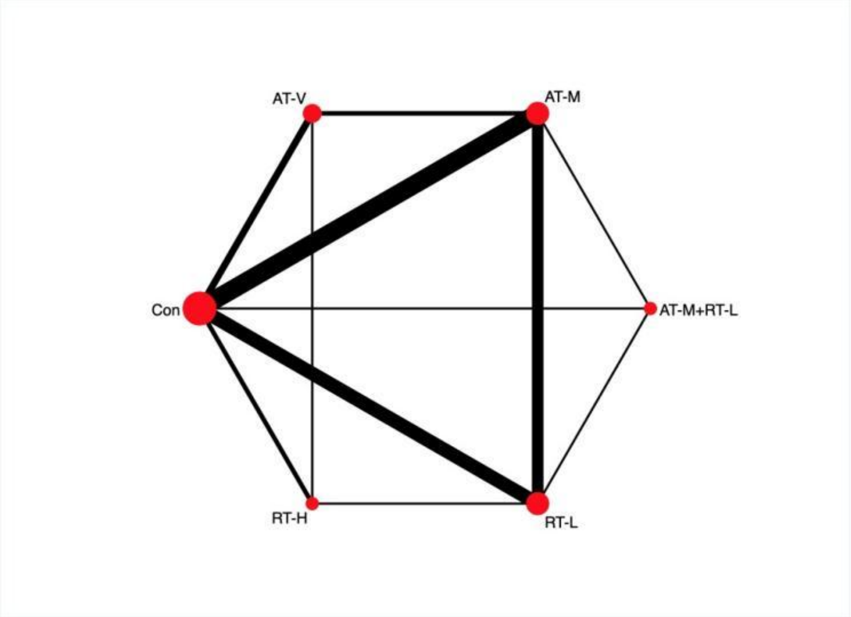

Figure7.1.2.1 Network plot of 2hPG.The size of the modes corresponds to the number of participants randomized to each physical activity type.Physical activity type with direct comparisons are linked with a line; Its thickness corresponds to the number of trials evaluating the comparison.RT-L: Resistance training of low-moderate load, AT-M: Aerobic training of moderate intensity, AT-M+RT-L: Combined moderate intensity aerobic exercise with low to moderate load resistance training, AT-V: Aerobic training of vigorous intensity, RT-H: Resistance training of high load, Con: No exercise.

7.1.2.2 The league table of 2hPG

2hPG	Comparison of treatments: Mean different (95% confidence intervals) / Effect of intervention in each row compared with intervention in each column				
AT-V	0.80 (-1.24; 2.84)	NA	-0.25 (-1.43; 0.93)	NA	-0.91 (-1.68; -0.14)*
-0.14 (-1.01; 0.74)	AT-M+RT-L	NA	-0.39 (-1.50; 0.72)	-0.48 (-1.58; 0.62)	-0.15 (-1.21; 0.91)
0.03 (-1.96; 2.03)	0.17 (-1.84; 2.18)	RT-H	NA	-0.27 (-2.32; 1.78)	-1.14 (-3.25; 0.97)
-0.24 (-0.91; 0.42)	-0.11 (-0.82; 0.61)	-0.28 (-2.19; 1.64)	AT-M	-0.33 (-0.73; 0.08)	-0.60 (-0.98; -0.21)*
-0.52 (-1.21; 0.18)	-0.38 (-1.10; 0.34)	-0.55 (-2.44; 1.35)	-0.27 (-0.65; 0.10)	RT-L	-0.34 (-0.74; 0.05)
-0.78 (-1.40; -0.15)*	-0.64 (-1.33; 0.06)	-0.81 (-2.71; 1.09)	-0.53 (-0.88; -0.18)*	-0.26 (-0.62; 0.10)	CON

Table 7.1.2.2: League Table of 2hPG.

7.1.3 HbA1c

7.1.3.1 Network plot of HbA1c

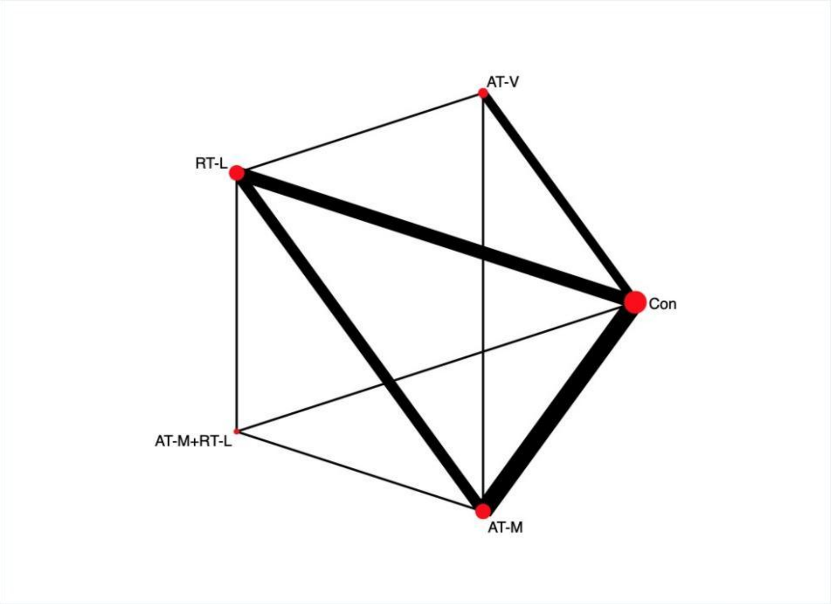

Figure7.1.3.1 Network plot of HbA1c.The size of the modes corresponds to the number of participants randomized to each physical activity type.Physical activity type with direct comparisons are linked with a line; Its thickness corresponds to the number of trials evaluating the comparison.RT-L: Resistance training of low-moderate load, AT-M: Aerobic training of moderate intensity, AT-M+RT-L: Combined moderate intensity aerobic exercise with low to moderate load resistance training, AT-V: Aerobic training of vigorous intensity, Con: No exercise.

7.1.3.2 The league table of HbA1c

HbA1c	Comparison of treatments: Mean different (95% confidence intervals) / Effect of intervention in each row compared with intervention in each column			
AT-M+RT-L	-0.01 (-0.28; 0.26)	0.20 (-0.09; 0.49)	NA	-0.54 (-0.87; -0.21)*
-0.05 (-0.25; 0.15)	AT-M	-0.02 (-0.13; 0.08)	0.09 (-0.13; 0.31)	-0.27 (-0.35; -0.18)*
-0.05 (-0.26; 0.15)	-0.00 (-0.09; 0.09)	RT-L	0.10 (-0.11; 0.31)	-0.25 (-0.35; -0.16)*
-0.12 (-0.34; 0.10)	-0.07 (-0.19; 0.05)	-0.07 (-0.19; 0.06)	AT-V	-0.09 (-0.21; 0.04)
-0.30 (-0.50; -0.10)*	-0.25 (-0.32; -0.17)*	-0.24 (-0.33; -0.16)*	-0.18 (-0.29; -0.07)*	CON

Table 7.1.3.2: League Table of HbA1c.

7.1.4 BMI

7.1.4.1 Network plot of BMI

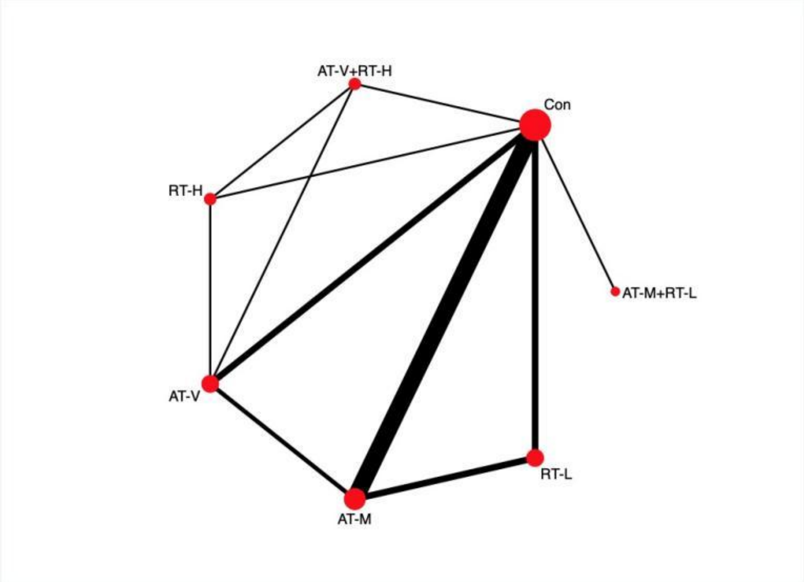

Figure7.1.4.1 Network plot of BMI.The size of the modes corresponds to the number of participants randomized to each physical activity type.Physical activity type with direct comparisons are linked with a line; Its thickness corresponds to the number of trials evaluating the comparison.RT-L: Resistance training of low-moderate load, AT-M: Aerobic training of moderate intensity, AT-M+RT-L: Combined moderate intensity aerobic exercise with low to moderate load resistance training, AT-V: Aerobic training of vigorous intensity, AT-V+RT-H: Combined vigorous intensity aerobic exercise with high load resistance training, RT-H: Resistance training of high load, Con: No exercise.

7.1.4.2 The league table of BMI

BMI	Comparison of treatments: Mean different (95% confidence intervals) / Effect of intervention in each row compared with intervention in each column					
AT-M+RT-L	NA	NA	NA	NA	NA	-1.20 (-3.76; 1.36)
-0.49 (-3.07; 2.09)	AT-M	0.02 (-0.36; 0.39)	NA	-0.35 (-1.11; 0.40)	NA	-0.71 (-1.01; -0.40)*
-0.59 (-3.18; 2.00)	-0.10 (-0.46; 0.25)	RT-L	NA	NA	NA	-0.48 (-0.86; -0.10)*
-0.70 (-3.86; 2.46)	-0.21 (-2.07; 1.65)	-0.11 (-1.99; 1.77)	AT-V+RT-H	-0.10 (-2.71; 2.51)	-0.30 (-4.42; 3.82)	-0.50 (-3.14; 2.14)
-0.79 (-3.42; 1.83)	-0.31 (-0.85; 0.24)	-0.21 (-0.82; 0.41)	-0.10 (-1.95; 1.76)	AT-V	-0.20 (-4.34; 3.94)	-0.45 (-1.18; 0.28)
-1.00 (-4.87; 2.87)	-0.51 (-3.42; 2.40)	-0.41 (-3.33; 2.51)	-0.30 (-3.45; 2.85)	-0.20 (-3.12; 2.71)	RT-H	-0.20 (-4.36; 3.96)
-1.20 (-3.76; 1.36)	-0.71 (-1.00; -0.42)*	-0.61 (-0.97; -0.25)*	-0.50 (-2.35; 1.35)	-0.41 (-0.94; 0.13)	-0.20 (-3.10; 2.70)	CON

Table 7.1.4.2: League Table of BMI.

7.1.5 Weight

7.1.5.1 Network plot of Weight

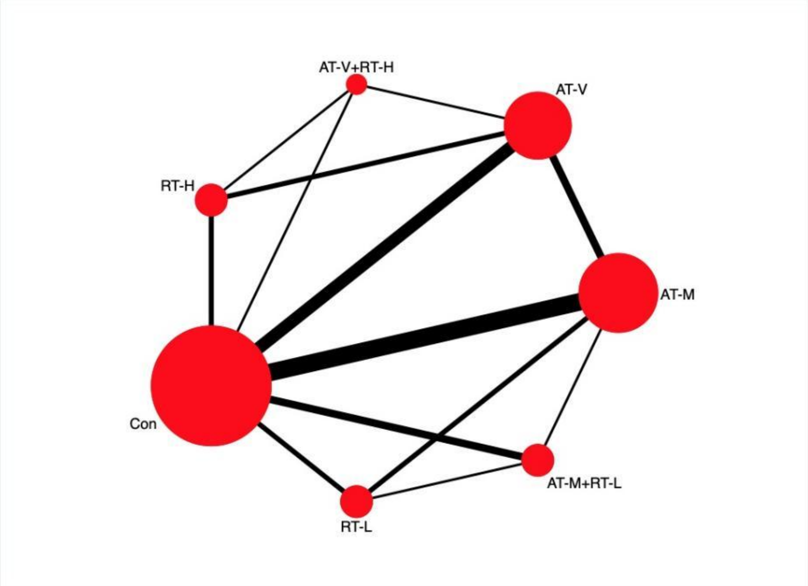

Figure7.1.5.1 Network plot of Weight.The size of the modes corresponds to the number of participants randomized to each physical activity type.Physical activity type with direct comparisons are linked with a line; Its thickness corresponds to the number of trials evaluating the comparison.RT-L: Resistance training of low-moderate load, AT-M: Aerobic training of moderate intensity, AT-M+RT-L: Combined moderate intensity aerobic exercise with low to moderate load resistance training, AT-V: Aerobic training of vigorous intensity, AT-V+RT-H: Combined vigorous intensity aerobic exercise with high load resistance training, RT-H: Resistance training of high load, Con: No exercise.

7.1.5.2 The league table of Weight

Weight	Comparison of treatments: Mean different (95% confidence intervals) / Effect of intervention in each row compared with intervention in each column					
AT-M+RT-L	-1.64 (-6.25; 2.97)	NA	NA	-3.19 (-8.04; 1.66)	NA	-3.14 (-6.50; 0.23)
-1.06 (-3.79; 1.68)	AT-M	-0.21 (-2.12; 1.71)	NA	-0.51 (-3.11; 2.09)	NA	-2.83 (-4.26; -1.40)
-1.49 (-4.40; 1.42)	-0.44 (-1.94; 1.07)	AT-V	0.00 (-6.52; 6.52)	NA	-1.45 (-4.42; 1.51)	-2.47 (-4.09; -0.85)*
-2.05 (-7.72; 3.63)	-0.99 (-6.13; 4.15)	-0.55 (-5.59; 4.48)	AT-V+RT-H	NA	-0.60 (-13.81; 12.61)	-1.00 (-8.88; 6.88)
-2.11 (-5.19; 0.96)	-1.06 (-3.26; 1.14)	-0.62 (-3.08; 1.83)	-0.07 (-5.54; 5.40)	RT-L	NA	-1.40 (-3.99; 1.19)
-3.04 (-6.72; 0.65)	-1.98 (-4.78; 0.82)	-1.54 (-4.17; 1.08)	-0.99 (-6.51; 4.53)	-0.92 (-4.27; 2.43)	RT-H	-0.59 (-3.54; 2.35)
-3.72 (-6.34; -1.09)	-2.66 (-3.92; -1.40)*	-2.23 (-3.62; -0.83)*	-1.67 (-6.73; 3.38)	-1.60 (-3.76; 0.55)	-0.68 (-3.30; 1.93)	CON

Table 7.1.5.2: League Table of Weight.

7.1.6 TC

7.1.6.1 Network plot of TC

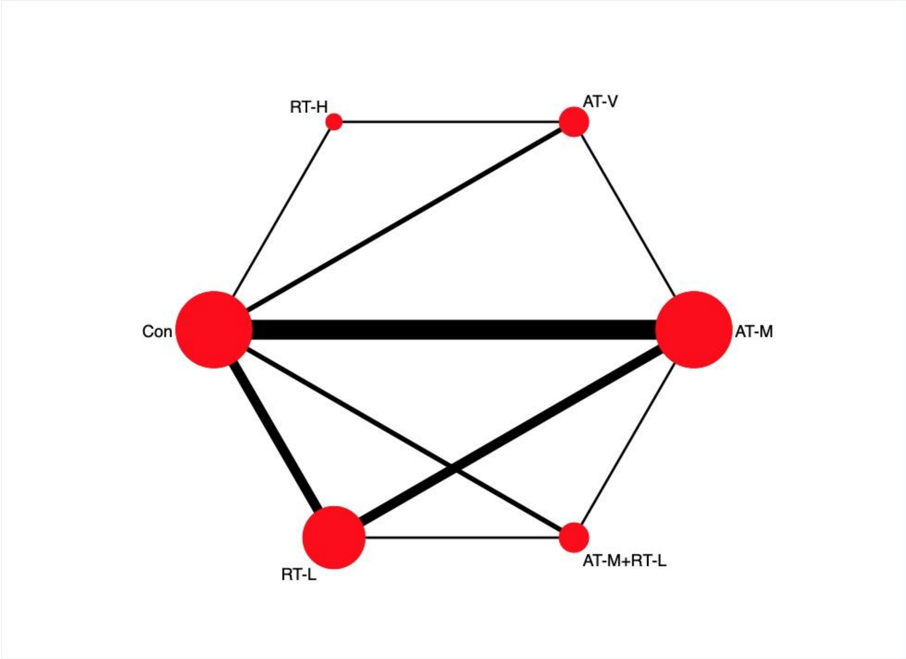

Figure7.1.6.1 Network plot of TC.The size of the modes corresponds to the number of participants randomized to each physical activity type.Physical activity type with direct comparisons are linked with a line; Its thickness corresponds to the number of trials evaluating the comparison.RT-L: Resistance training of low-moderate load, AT-M: Aerobic training of moderate intensity, AT-M+RT-L: Combined moderate intensity aerobic exercise with low to moderate load resistance training, AT-V: Aerobic training of vigorous intensity, RT-H: Resistance training of high load, Con: No exercise.

7.1.6.2 The league table of TC

TC	Comparison of treatments: Mean different (95% confidence intervals) / Effect of intervention in each row compared with intervention in each column				
AT-M+RT-L	-0.18 (-0.72; 0.36)	-0.13 (-0.66; 0.40)	NA	NA	-1.15 (-1.63; -0.67)*
-0.45 (-0.80; -0.11)*	AT-M	-0.04 (-0.27; 0.18)	0.02 (-0.38; 0.41)	NA	-0.29 (-0.45; -0.13)*
-0.45 (-0.81; -0.10)*	-0.00 (-0.20; 0.20)	RT-L	NA	NA	-0.26 (-0.48; -0.04)*
-0.46 (-0.86; -0.06)*	-0.01 (-0.25; 0.23)	-0.01 (-0.30; 0.28)	AT-V	-0.30 (-0.64; 0.04)	-0.32 (-0.59; -0.05)*
-0.73 (-1.19; -0.27)*	-0.28 (-0.62; 0.07)	-0.28 (-0.65; 0.09)	-0.27 (-0.58; 0.05)	RT-H	-0.10 (-0.44; 0.24)
-0.80 (-1.13; -0.46)*	-0.34 (-0.49; -0.19)*	-0.34 (-0.54; -0.15)*	-0.33 (-0.56; -0.11)*	-0.07 (-0.38; 0.25)	CON

Table 7.1.6.2: League Table of TC.

7.1.7 SBP

7.1.7.1 Network plot of SBP

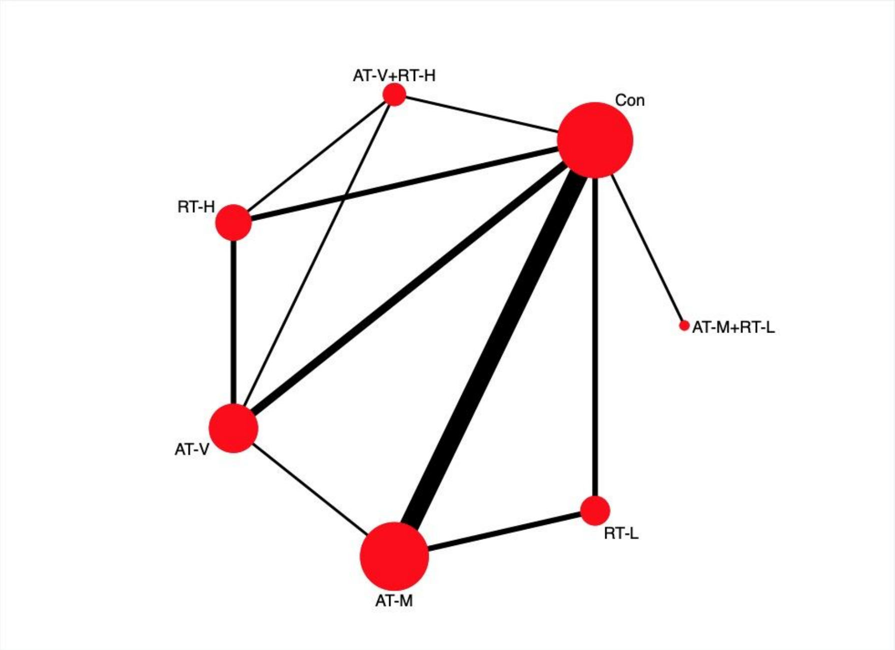

Figure7.1.7.1 Network plot of SBP.The size of the modes corresponds to the number of participants randomized to each physical activity type.Physical activity type with direct comparisons are linked with a line; Its thickness corresponds to the number of trials evaluating the comparison.RT-L: Resistance training of low-moderate load, AT-M: Aerobic training of moderate intensity, AT-M+RT-L: Combined moderate intensity aerobic exercise with low to moderate load resistance training, AT-V: Aerobic training of vigorous intensity, AT-V+RT-H: Combined vigorous intensity aerobic exercise with high load resistance training, RT-H: Resistance training of high load, Con: No exercise.

7.1.7.2 The league table of SBP

SBP	Comparison of treatments: Mean different (95% confidence intervals) / Effect of intervention in each row compared with intervention in each column					
AT-V	**NA**	NA	-5.90(-12.83; 1.03)	-3.31(-10.49; 3.87)	-9.00 (-21.75; 3.75)	-4.57 (-9.73; 0.58)
3.46 (-15.78; 22.71)	AT-M+RT-L	NA	NA	NA	NA	-11.00 (-29.81; 7.81)
-2.14 (-8.19; 3.91)	-5.61 (-25.01; 13.80)	RT-L	1.90 (-3.38; 7.19)	NA	NA	-7.37 (-12.57; -2.18)*
-2.36 (-6.76; 2.05)	-5.82 (-24.85; 13.21)	-0.22 (-5.01; 4.57)	AT-M	NA	NA	-5.84 (-8.89; -2.79)*
-3.00 (-9.17; 3.16)	-6.47 (-26.21; 13.28)	-0.86 (-8.45; 6.72)	-0.65(-7.12; 5.82)	RT-H	-3.60 (-17.10; 9.90)	-3.56 (-10.58; 3.45)
-5.14 (-13.33; 3.05)	-8.60 (-29.01; 11.80)	-3.00 (-12.17; 6.17)	-2.78(-11.07; 5.51)	-2.14 (-11.01; 6.74)	AT-V+RT-H	-5.00 (-15.66; 5.66)
-7.54 (-11.61; -3.47)*	-11.00(-29.81; 7.81)	-5.39 (-10.15; -0.64)*	-5.18 (-8.05; -2.31)*	-4.53 (-10.53; 1.47)*	-0.31 (-0.94; 0.32)	CON

Table 7.1.7.2: League Table of SBP.

7.1.8 DBP

7.1.8.1 Network plot of DBP

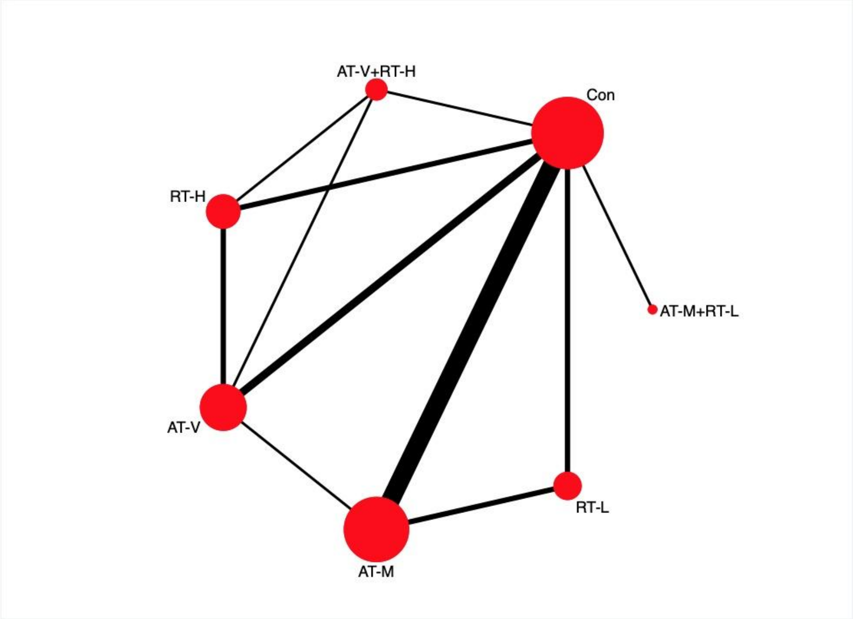

Figure7.1.8.1 Network plot of DBP.The size of the modes corresponds to the number of participants randomized to each physical activity type.Physical activity type with direct comparisons are linked with a line; Its thickness corresponds to the number of trials evaluating the comparison.RT-L: Resistance training of low-moderate load, AT-M: Aerobic training of moderate intensity, AT-M+RT-L: Combined moderate intensity aerobic exercise with low to moderate load resistance training, AT-V: Aerobic training of vigorous intensity, AT-V+RT-H: Combined vigorous intensity aerobic exercise with high load resistance training, RT-H: Resistance training of high load, Con: No exercise.

7.1.8.2 The league table of DBP

DBP	Comparison of treatments: Mean different (95% confidence intervals) / Effect of intervention in each row compared with intervention in each column					
AT-M	-0.36 (-4.38; 3.65)	-1.80 (-6.65; 3.05)	NA	NA	NA	-3.24 (-5.33; -1.15)
-0.27 (-3.94; 3.41)	RT-L	NA	NA	NA	NA	-3.64 (-7.64; 0.35)
-0.81 (-3.84; 2.22)	-0.54(-5.01; 3.93)	AT-V	-0.76 (-5.61; 4.09)	NA	-3.20 (-13.31; 6.91)	-2.74 (-6.14; 0.66)
-1.54 (-6.17; 3.08)	-1.28(-6.90; 4.34)	-0.73 (-5.05; 3.58)	RT-H	NA	-3.80 (-17.33; 9.73)	-1.18 (-6.17; 3.80)
-2.43 (-11.53; 6.67)	-2.16(-11.77; 7.45)	-1.62 (-10.93; 7.69)	-0.88(-10.77; 9.00)	AT-M+RT-L	NA	-1.00 (-9.89; 7.89)
-2.42 (-9.71; 4.87)	-2.15(-10.11; 5.81)	-1.61 (-8.75; 5.54)	-0.87(-8.70; 6.95)	0.01 (-11.36; 11.38)	AT-V+RT-H	-3.10 (-13.17; 6.97)
-3.43 (-5.39; -1.46)*	-3.16(-6.83; 0.51)	-2.62 (-5.40; 0.16)	-1.88(-6.21; 2.44)	-1.00 (-9.89; 7.89)	-1.01 (-8.11; 6.09)	CON

Table 7.1.8.2: League Table of DBP.

7.1.9 LDL

7.1.9.1 Network plot of LDL

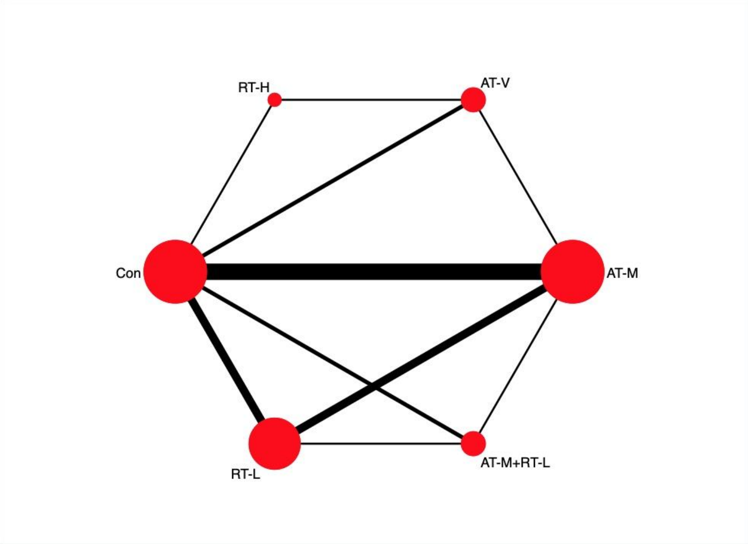

Figure7.1.9.1 Network plot of LDL.The size of the modes corresponds to the number of participants randomized to each physical activity type.Physical activity type with direct comparisons are linked with a line; Its thickness corresponds to the number of trials evaluating the comparison.RT-L: Resistance training of low-moderate load, AT-M: Aerobic training of moderate intensity, AT-M+RT-L: Combined moderate intensity aerobic exercise with low to moderate load resistance training, AT-V: Aerobic training of vigorous intensity, RT-H: Resistance training of high load, Con: No exercise.

7.1.9.2 The league table of LDL

LDL	Comparison of treatments: Mean different (95% confidence intervals) / Effect of intervention in each row compared with intervention in each column				
AT-M+RT-L	-0.26 (-0.71; 0.19)	-0.57 (-1.03; -0.11)*	NA	NA	-0.61 (-1.09; -0.13)*
-0.32 (-0.65; 0.00)	RT-L	-0.10 (-0.32; 0.11)	NA	NA	-0.34 (-0.56; -0.12)*
-0.49 (-0.81; -0.18)*	-0.17 (-0.36; 0.02)	AT-M	-0.09 (-0.45; 0.27)	NA	-0.16 (-0.31; -0.01)*
-0.77 (-1.14; -0.40)*	-0.44 (-0.71; -0.18)*	-0.28 (-0.50; -0.06)*	AT-V	-0.10 (-0.39; 0.19)	0.23 (-0.01; 0.47)
-0.94 (-1.35; -0.53)*	-0.62 (-0.94; -0.29)*	-0.45 (-0.74; -0.16)*	-0.17 (-0.44; 0.10)	RT-H	0.40 (0.11; 0.69)
-0.62 (-0.93; -0.30)*	-0.29 (-0.48; -0.10)*	-0.12 (-0.26; 0.01)	0.15 (-0.05; 0.35)	0.33 (0.06; 0.60)	CON

Table 7.1.9.2: League Table of LDL.

NOTE: All results are presented in the form of MD (95% CrI). Physical activity types are ranked according to the surface under the curve cumulative for balance starting with the best from left to right. The results of the network meta-analysis are showed in the lower left part, and results from pairwise comparisons in the upper right half (if available). *: Significant influence factors; NA: not available; MD: Mean Difference; CrI: Credible Interval; AT-M: Aerobic training of moderate intensity; AT-V: Aerobic training of vigorous intensity;RT-L: Resistance training of low-moderate load;RT-H: Resistance training of high load; AT-M+RT-L: Combined moderate intensity aerobic exercise with low~moderate load resistance training; AT-V+RT-H: Combined vigorous intensity aerobic exercise with high load resistance training； CON: Control.

7.2 Forest plots

7.2.1 Forest plot of outcome FBG

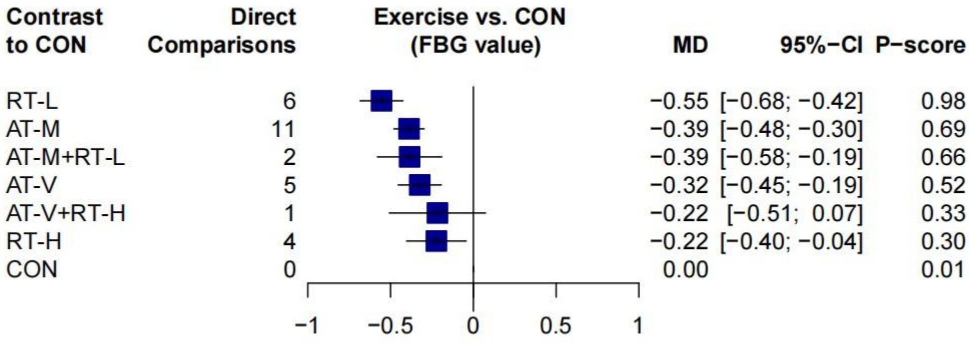

Figure 7.2.1: Exercise training type are ranked according to MD compared to CON. Treatments crossing the y-axis are not significantly different from CON. *MD* Mean Difference, *RT-L* Resistance training of low-moderate load, *AT-M* Aerobic training of moderate intensity, *AT-M+RT-L* Combined moderate intensity aerobic exercise with low to moderate load resistance training, *AT-V* Aerobic training of vigorous intensity, *AT-V+RT-H* Combined vigorous intensity aerobic exercise with high load resistance training, *RT-H* Resistance training of high load.

7.2.2 Forest plot of outcome 2hPG

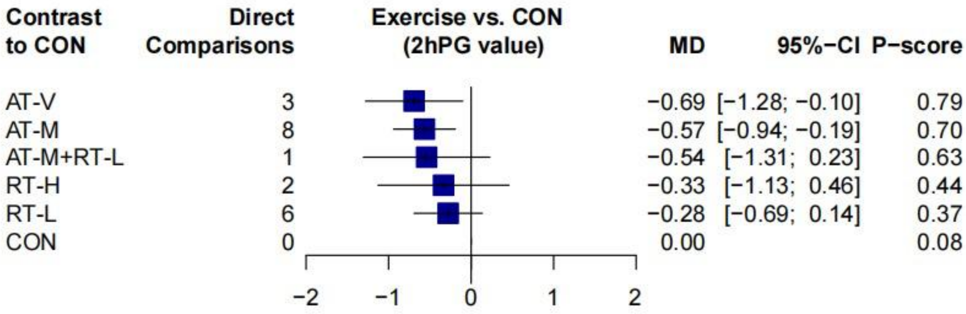

Figure 7.2.2: Exercise training type are ranked according to MD compared to CON. Treatments crossing the y-axis are not significantly different from CON. *MD* Mean Difference, *AT-V* Aerobic training of vigorous intensity, *AT-M* Aerobic training of moderate intensity, *AT-M+RT-L* Combined moderate intensity aerobic exercise with low to moderate load resistance training, *RT-H* Resistance training of high load, *RT-L* Resistance training of low-moderate load.

7.2.3 Forest plot of outcome HbA1c

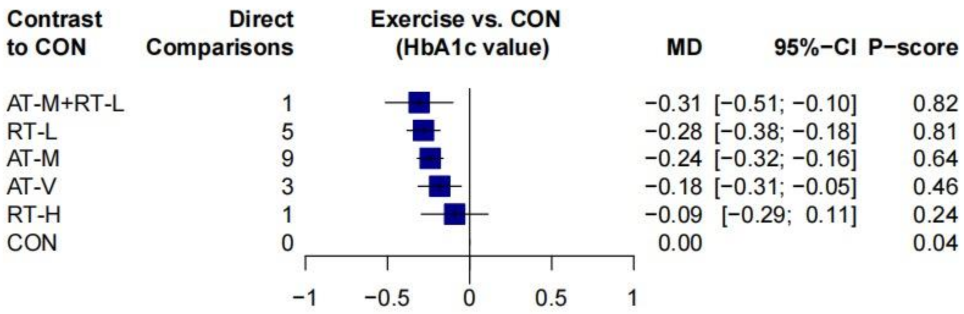

Figure 7.2.3: Exercise training type are ranked according to MD compared to CON. Treatments crossing the y-axis are not significantly different from CON. *MD* Mean Difference, *AT-M+RT-L* Combined moderate intensity aerobic exercise with low to moderate load resistance training, *RT-L* Resistance training of low-moderate load, *AT-M* Aerobic training of moderate intensity, *AT-V* Aerobic training of vigorous intensity, *RT-H* Resistance training of high load.

7.2.4 Forest plot of outcome Weight

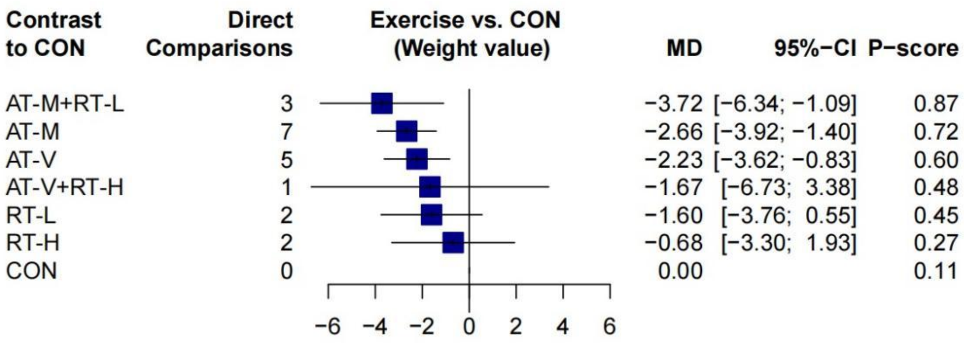

Figure 7.2.4: Exercise training type are ranked according to MD compared to CON. Treatments crossing the y-axis are not significantly different from CON. *MD* Mean Difference, *RT-L* Resistance training of low-moderate load, *AT-M* Aerobic training of moderate intensity, *AT-M+RT-L* Combined moderate intensity aerobic exercise with low to moderate load resistance training, *AT-V* Aerobic training of vigorous intensity, *AT-V+RT-H* Combined vigorous intensity aerobic exercise with high load resistance training, *RT-H* Resistance training of high load.

7.2.5 Forest plot of outcome BMI

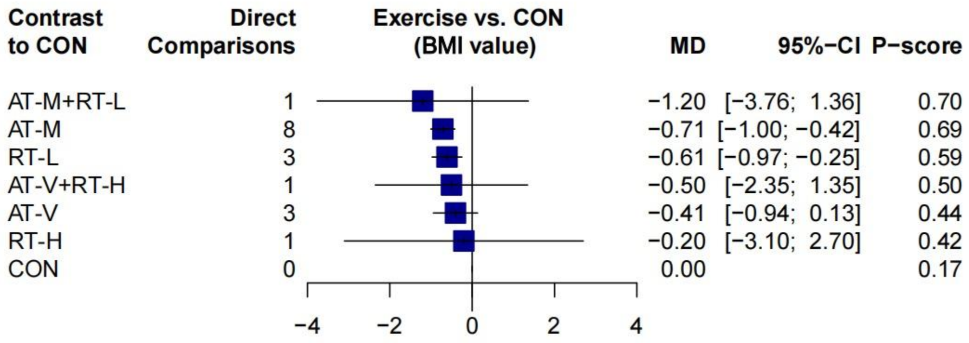

Figure 7.2.5: Exercise training type are ranked according to MD compared to CON. Treatments crossing the y-axis are not significantly different from CON. MD Mean Difference, *RT-L* Resistance training of low-moderate load, *AT-M* Aerobic training of moderate intensity, *AT-M+RT-L* Combined moderate intensity aerobic exercise with low to moderate load resistance training, *AT-V* Aerobic training of vigorous intensity, *AT-V+RT-H* Combined vigorous intensity aerobic exercise with high load resistance training, *RT-H* Resistance training of high load.

7.2.6 Forest plot of outcome TC

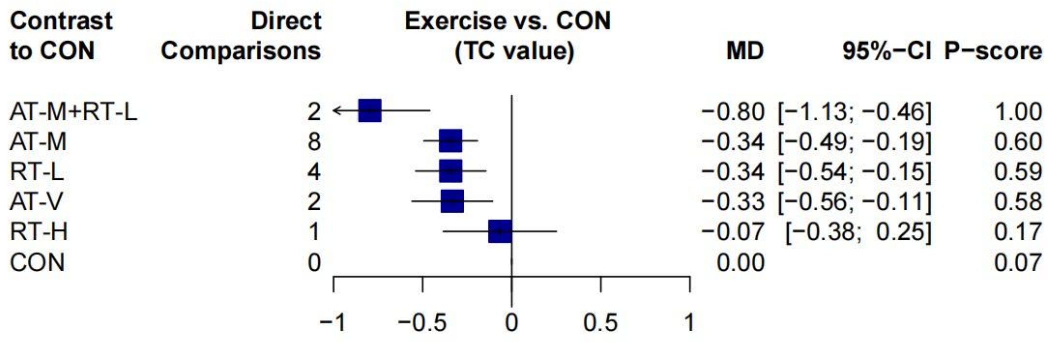

Figure 7.2.6: Exercise training type are ranked according to MD compared to CON. Treatments crossing the y-axis are not significantly different from CON. *MD* Mean Difference, *AT-M+RT-L* Combined moderate intensity aerobic exercise with low to moderate load resistance training, *AT-M* Aerobic training of moderate intensity, *RT-L* Resistance training of low-moderate load, *AT-V* Aerobic training of vigorous intensity, *RT-H* Resistance training of high load.

7.2.7 Forest plot of outcome SBP

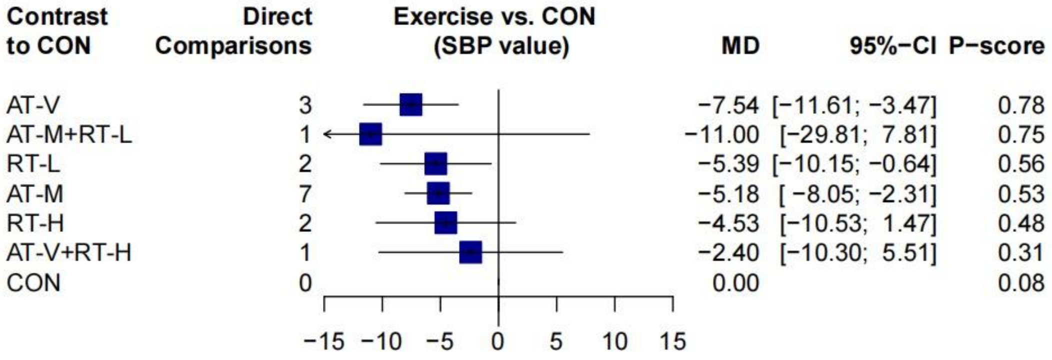

Figure 7.2.7: Exercise training type are ranked according to MD compared to CON. Treatments crossing the y-axis are not significantly different from CON. *MD* Mean Difference, *AT-M+RT-L* Combined moderate intensity aerobic exercise with low to moderate load resistance training, *AT-M* Aerobic training of moderate intensity, *RT-L* Resistance training of low-moderate load, *AT-V* Aerobic training of vigorous intensity, *RT-H* Resistance training of high load, *AT-V+RT-H* Combined vigorous intensity aerobic exercise with high load resistance training

7.2.8 Forest plot of outcome DBP

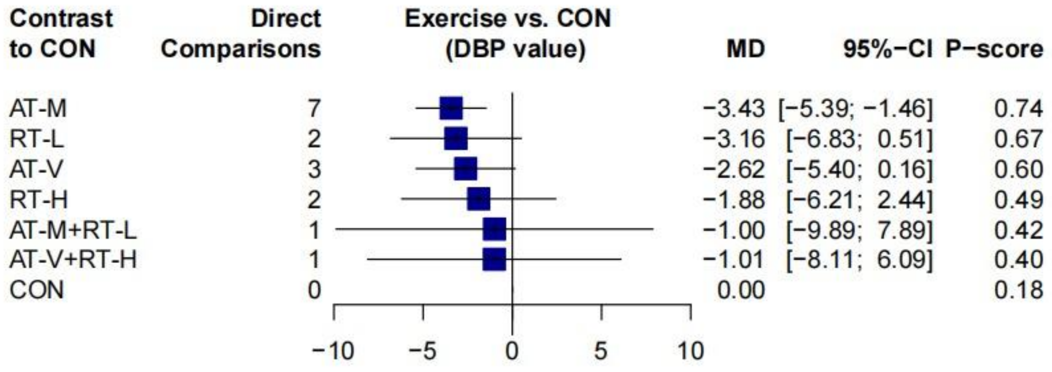

Figure 7.2.8: Exercise training type are ranked according to MD compared to CON. Treatments crossing the y-axis are not significantly different from CON. MD Mean Difference, *RT-L* Resistance training of low-moderate load, *AT-M* Aerobic training of moderate intensity, *AT-M+RT-L* Combined moderate intensity aerobic exercise with low to moderate load resistance training, *AT-V* Aerobic training of vigorous intensity, *AT-V+RT-H* Combined vigorous intensity aerobic exercise with high load resistance training, *RT-H* Resistance training of high load.

7.2.9 Forest plot of outcome LDL

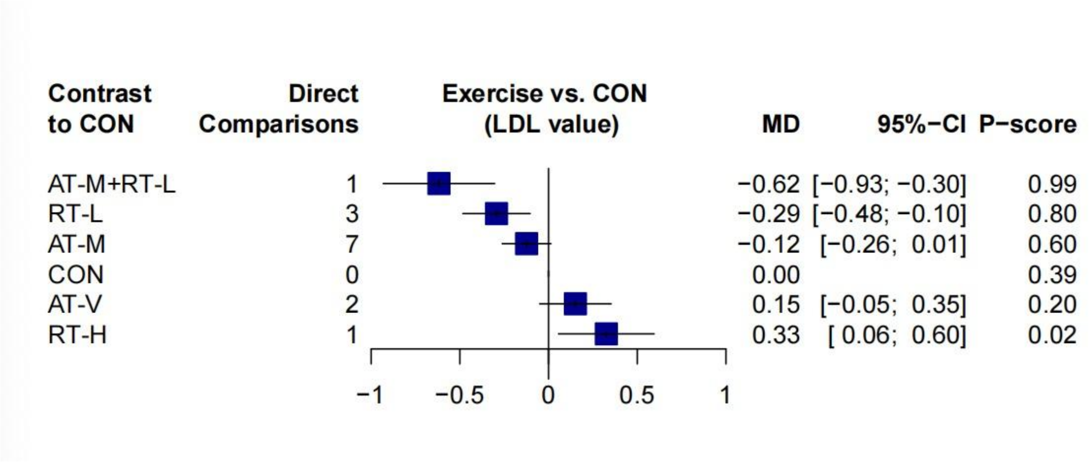

Figure 7.2.9: Exercise training type are ranked according to MD compared to CON. Treatments crossing the y-axis are not significantly different from CON. *MD* Mean Difference, *RT-L* Resistance training of low-moderate load, *AT-M* Aerobic training of moderate intensity, *AT-M+RT-L* Combined moderate intensity aerobic exercise with low to moderate load resistance training, *AT-V* Aerobic training of vigorous intensity, *AT-V+RT-H* Combined vigorous intensity aerobic exercise with high load resistance training, *RT-H* Resistance training of high load.

Appendix8: Evaluation of heterogeneity

We use the tau square (τ2) test and p-value to qualitatively analyze the statistical heterogeneity between the studies. The larger the τ2 and the smaller the p-value, the greater the possibility of heterogeneity; on the contrary, the smaller the existence heterogeneity. In addition, I2 is a parameter for quantitative analysis of the heterogeneity between the results of each study. It' s value is distributed from 0-100%. When I^2 is less than 25%, it means that the heterogeneity is low; 25%-50% means that the heterogeneity is moderate; I^2 > 75% means high heterogeneity. In summary, when I2 > 50%, it means that there is substantial heterogeneity.

Primary outcomes	τ2	Q	df	P	I^2	Heterogeneity assessment
FBG	0.0174	83.72	30	0.0001	64.2%	Moderate to high
2hPG	4.9848	33.66	11	0.0004	67.3%	Moderate to high
HbA1c	0.0123	98.32	17	0.0001	82.7%	High
BMI	0.0572	21.92	15	0.1098	31.6%	Moderate
Weight	2.1237	72.89	20	0.0001	72.6%	Moderate to high
TC	0.0301	46.9	15	0.0001	68%	Moderate to high
LDL	0.0212	32.57	12	0.0011	63.2%	Moderate to high
SBP	9.2324	30.43	13	0.0041	57.3%	Moderate to high
DBP	5.1510	40.13	13	0.0001	67.6%	Moderate to high

Appendix 9: Evaluation of inconsistency

Outcomes	Number of studies	SIDE splitting		the Design-by-Treatment test			
		Number of inconsistent comparisons out of total	Percentage of inconsistent comparisons out of total	Q	df	τ2	p-value
FBG	44	6	13.63%	12.60	11	0.0168	0.3200
2hPG	31	0	0%	17.47	8	0.1028	0.0256
HbA1c	28	2	7.14%	31.31	7	0.0064	0.0001
BMI	25	6	24%	11.25	4	0	0.0239
Weight	31	0	0%	1.62	11	7.7422	0.9994
TC	25	2	8%	15.53	6	0.0260	0.0165
LDL	21	0	0%	26.61	6	0	0.0002
SBP	23	0	0%	16.26	6	0.9719	0.0124
DBP	23	0	0%	2.87	6	12.8886	0.8253

Appendix10: Network Meta-Regression--changes in heterogeneity

10.1 Outcome of Network Meta-Regression

Covariate	Shared beta (median and 95%Crl)				
	FBG	2hPG	HbA1c	BMI	Weight
Publish Year	-0.059(-0.236,0.117)	-0.313(-1.056,0.545)	-0.189(-0.491,0.087)	-0.189(-0.491,0.087)	-0.164(-2.758,2.149)
Sampe Size	-0.018(-0.202,0.151)	**-0.508(-0.991,-0.079)***	0.081(-0.129,0.304)	0.081(-0.129,0.304)	-0.168(-2.449,2.165)
Percentage of Male	0.138(-0.020,0.316)	0.235(-0.391,0.866)	0.176(-0.046,0.421)	0.176(-0.046,0.421)	-0.674(-3.043,2.186)
Mean Age	**-0.180(-0.366,-0.006)***	0.142(-0.740,0.992)	-0.181(-0.449,0.129)	-0.181(-0.449,0.129)	0.076(-2.914,2.707)
Exercise Frequency	0.129(-0.047,0.298)	0.246(-0.578,1.039)	0.058(-0.179,0.315)	0.058(-0.179,0.315)	-0.209(-2.236,1.667)
Exercise Period	-0.086(-0.287,0.108)	0.368(-0.355,1.089)	**-0.312(-0.570,-0.082)***	**-0.312(-0.570,-0.082)***	-1.986(-4.907,0.883)
Time of Single Session	-0.125(-0.355,0.116)	0.201(-0.954,1.150)	-0.025(-0.264,0.250)	-0.025(-0.264,0.250)	1.303(-1.098,3.818)
	TC	SBP	DBP	LDL	
Publish Year	**-0.285(-0.585,-0.002)***	0.547(-4.319,6.175)	**3.373(0.568,6.512)***	-0.141(-0.441,0.154)	
Sampe Size	0.064(-0.266,0.416)	0.037(-4.612,6.337)	1.521(-1.654,6.372)	-0.039(-0.256,0.217)	
Percentage of Male	0.274(-0.221,0.771)	3.852(-1.352,9.083)	0.377(-3.637,4.522)	0.208(-0.133,0.435)	
Mean Age	-0.227(-0.644,0.121)	-3.266(-9.151,2.410)	-1.118(-5.779,2.987)	-0.059(-0.329,0.248)	
Exercise Frequency	-0.024(-0.426,0.401)	1.344(-4.249,6.785)	1.532(-2.125,5.214)	0.106(-0.149,0.371)	
Exercise Period	**-0.561(-0.879,-0.228)***	2.156(-2.168,6.632)	0.928(-2.233,4.496)	-0.095(-0.389,0.254)	
Time of Single Session	-0.148(-0.568,0.249)	-2.985(-7.689,2.482)	0.465(-3.522,3.898)	0.005(-0.284,0.283)	

Crl:credible interval;*:significant influence factors, 95% Crl does not contain zero

10.2 FBG

10.2.1 Publication year

When the model was adjusted for centering value of publish year 2014, the hierarchy from the unadjusted model retained.

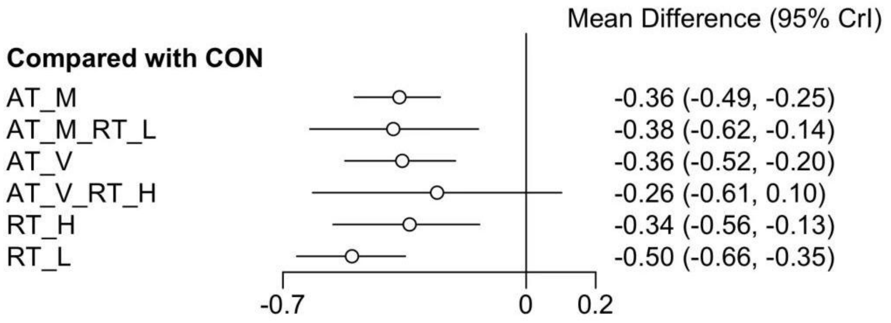

Figure 10.2.1: Forest plot overall change in general symptoms adjusted for publish year 2009. Exercise type are ranked according to MD compared to CON. Treatments crossing the y-axis are not significantly different from CON. *MD* Mean Difference, *RT-L* Resistance training of low-moderate load, *AT-M* Aerobic training of moderate intensity, *AT-M+RT-L* Combined moderate intensity aerobic exercise with low to moderate load resistance training, *AT-V* Aerobic training of vigorous intensity, *AT-V+RT-H* Combined vigorous intensity aerobic exercise with high load resistance training, *RT-H* Resistance training of high load.

10.2.2 Sample size

When the model was adjusted for centering value of sample size 74, the hierarchy from the unadjusted model retained.

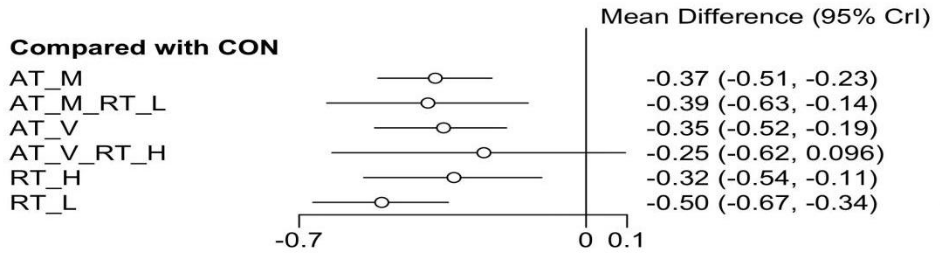

Figure 10.2.2: Forest plot overall change in general symptoms adjusted for sample size 74. Exercise type are ranked according to MD compared to CON. Treatments crossing the y-axis are not significantly different from CON. *MD* Mean Difference, *RT-L* Resistance training of low-moderate load, *AT-M* Aerobic training of moderate intensity, *AT-M+RT-L* Combined moderate intensity aerobic exercise with low to moderate load resistance training, *AT-V* Aerobic training of vigorous intensity, *AT-V+RT-H* Combined vigorous intensity aerobic exercise with high load resistance training, *RT-H* Resistance training of high load.

10.2.3 Percentage of Male

When the model was adjusted for centering value of male's percentage 31%, hierarchy from the unadjusted model retained.

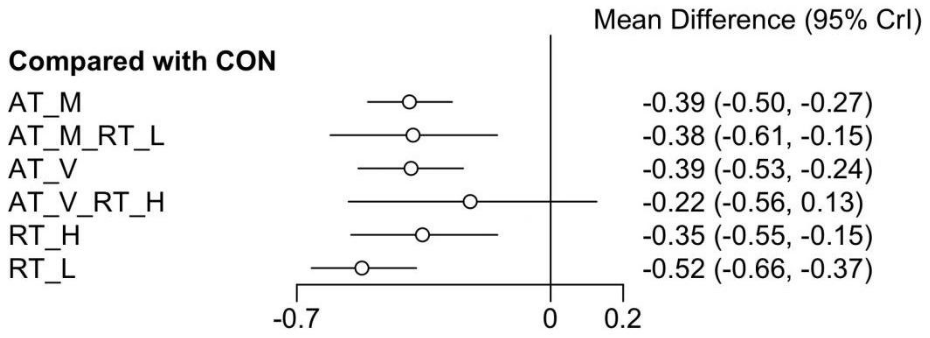

Figure 10.2.3: Forest plot overall change in general symptoms adjusted for male's percentage 31%. Exercise type are ranked according to MD compared to CON. Treatments crossing the y-axis are not significantly different from CON. *MD* Mean Difference, *RT-L* Resistance training of low-moderate load, *AT-M* Aerobic training of moderate intensity, *AT-M+RT-L* Combined moderate intensity aerobic exercise with low to moderate load resistance training, *AT-V* Aerobic training of vigorous intensity, *AT-V+RT-H* Combined vigorous intensity aerobic exercise with high load resistance training, *RT-H* Resistance training of high load.

10.2.4 Mean age

When the model was adjusted for centering value of mean age 59, the hierarchy from the unadjusted model retained.

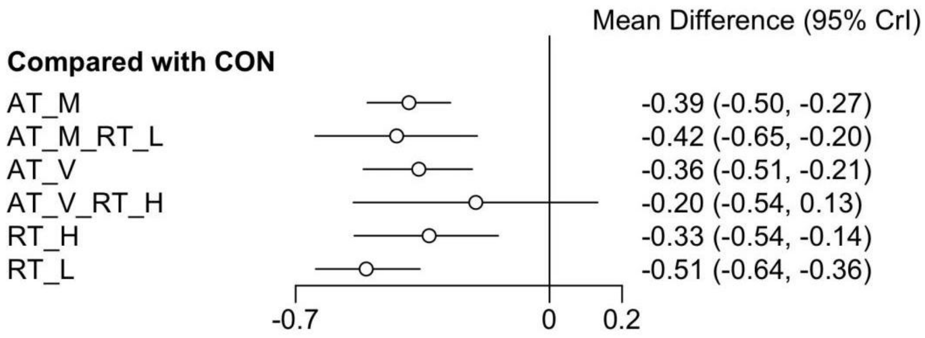

Figure 10.2.4: Forest plot overall change in general symptoms adjusted for mean age 59. Exercise type are ranked according to MD compared to CON. Treatments crossing the y-axis are not significantly different from CON. *MD* Mean Difference, *RT-L* Resistance training of low-moderate load, *AT-M* Aerobic training of moderate intensity, *AT-M+RT-L* Combined moderate intensity aerobic exercise with low to moderate load resistance training, *AT-V* Aerobic training of vigorous intensity, *AT-V+RT-H* Combined vigorous intensity aerobic exercise with high load resistance training, *RT-H* Resistance training of high load.

10.2.5 Exercise frequency

When the model was adjusted for centering value of exercise frequency 3(times/week), the hierarchy from the unadjusted model retained.

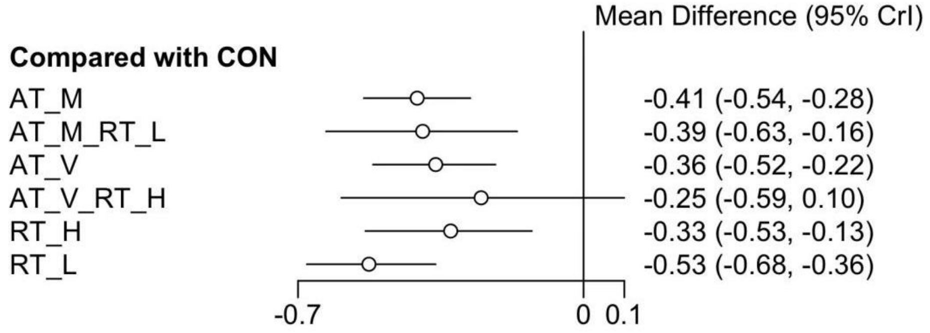

Figure 10.2.5: Forest plot overall change in general symptoms adjusted for exercise frequency 3(times/week). Exercise type are ranked according to MD compared to CON. Treatments crossing the y-axis are not significantly different from CON. *MD* Mean Difference, *RT-L* Resistance training of low-moderate load, *AT-M* Aerobic training of moderate intensity, *AT-M+RT-L* Combined moderate intensity aerobic exercise with low to moderate load resistance training, *AT-V* Aerobic training of vigorous intensity, *AT-V+RT-H* Combined vigorous intensity aerobic exercise with high load resistance training, *RT-H* Resistance training of high load.

10.2.6 Exercise period

When the model was adjusted for exercise period 32(weeks), the hierarchy from the unadjusted model retained.

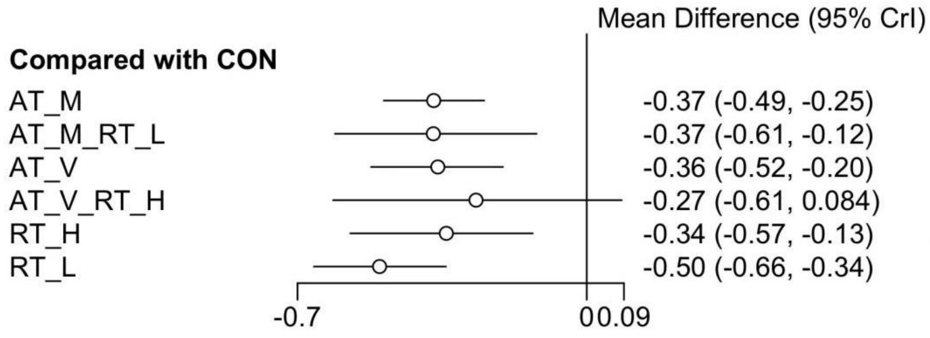

Figure 10.2.6: Forest plot overall change in general symptoms adjusted for exercise period 32(weeks). Exercise type are ranked according to MD compared to CON. Treatments crossing the y-axis are not significantly different from CON. *MD* Mean Difference, *RT-L* Resistance training of low-moderate load, *AT-M* Aerobic training of moderate intensity, *AT-M+RT-L* Combined moderate intensity aerobic exercise with low to moderate load resistance training, *AT-V* Aerobic training of vigorous intensity, *AT-V+RT-H* Combined vigorous intensity aerobic exercise with high load resistance training, *RT-H* Resistance training of high load.

10.2.7 Time of single session

When the model was adjusted for centering value of time of single session 52 minutes, the hierarchy from the unadjusted model retained.

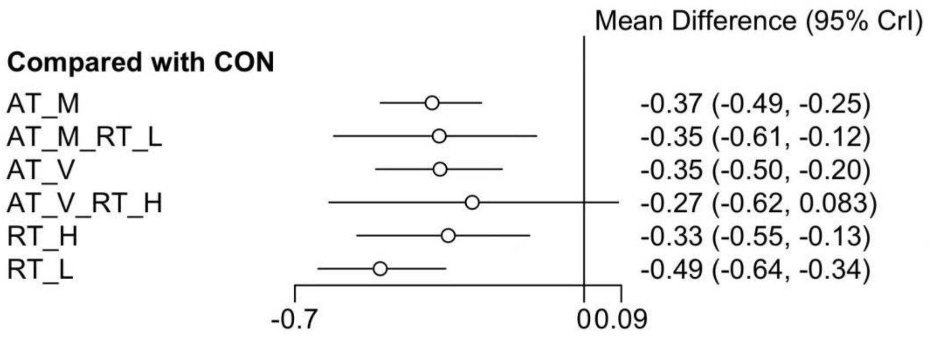

Figure 10.2.7: Forest plot overall change in general symptoms adjusted for time of single session 52 minutes. Exercise type are ranked according to MD compared to CON. Treatments crossing the y-axis are not significantly different from CON. *MD* Mean Difference, *RT-L* Resistance training of low-moderate load, *AT-M* Aerobic training of moderate intensity, *AT-M+RT-L* Combined moderate intensity aerobic exercise with low to moderate load resistance training, *AT-V* Aerobic training of vigorous intensity, *AT-V+RT-H* Combined vigorous intensity aerobic exercise with high load resistance training, *RT-H* Resistance training of high load.

10.3 2hPG

10.3.1 Publication year

When the model was adjusted for centering value of publish year 2015, the hierarchy from the unadjusted model retained.

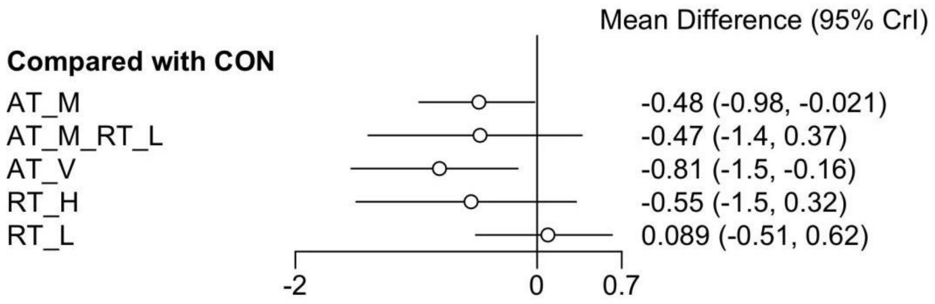

Figure 10.3.1: Forest plot overall change in general symptoms adjusted for publish year 2015. Exercise type are ranked according to MD compared to CON. Treatments crossing the y-axis are not significantly different from CON. *MD* Mean Difference, *RT-L* Resistance training of low-moderate load, *AT-M* Aerobic training of moderate intensity, *AT-M+RT-L* Combined moderate intensity aerobic exercise with low to moderate load resistance training, *AT-V* Aerobic training of vigorous intensity, *RT-H* Resistance training of high load.

10.3.2 Sample size

When the model was adjusted for centering value of sample size 81, hierarchy from the unadjusted model retained.

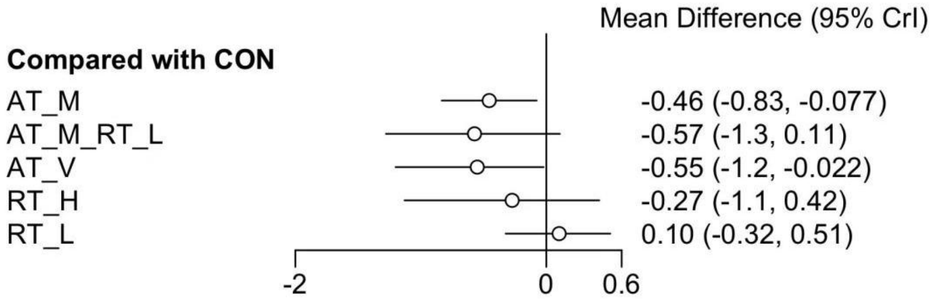

Figure 10.3.2: Forest plot overall change in general symptoms adjusted for sample size 81. Exercise type are ranked according to MD compared to CON. Treatments crossing the y-axis are not significantly different from CON. *MD* Mean Difference, *RT-L* Resistance training of low-moderate load, *AT-M* Aerobic training of moderate intensity, *AT-M+RT-L* Combined moderate intensity aerobic exercise with low to moderate load resistance training, *AT-V* Aerobic training of vigorous intensity, *RT-H* Resistance training of high load.

10.3.3 Percentage of male

When the model was adjusted for centering value of male's percentage 39%, the hierarchy from the unadjusted model retained.

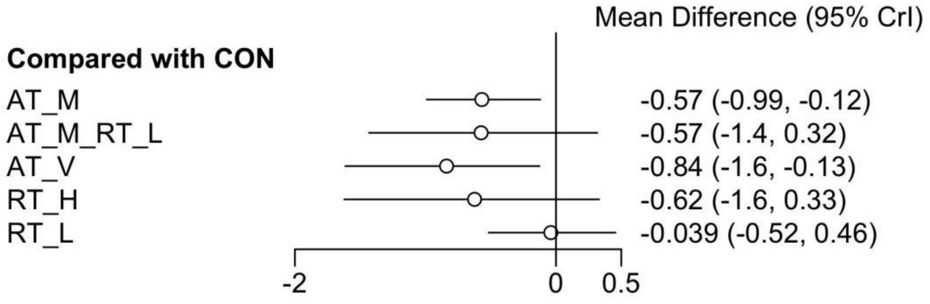

Figure 10.3.3: Forest plot overall change in general symptoms adjusted for male's percentage 39%. Exercise type are ranked according to MD compared to CON. Treatments crossing the y-axis are not significantly different from CON. *MD* Mean Difference, *RT-L* Resistance training of low-moderate load, *AT-M* Aerobic training of moderate intensity, *AT-M+RT-L* Combined moderate intensity aerobic exercise with low to moderate load resistance training, *AT-V* Aerobic training of vigorous intensity, *RT-H* Resistance training of high load.

10.3.4 Mean age

When the model was adjusted for centering value of mean age 60, the hierarchy from the unadjusted model retained.

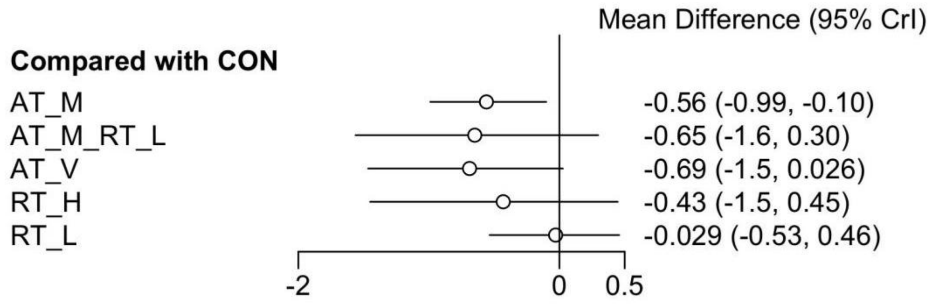

Figure 10.3.4: Forest plot overall change in general symptoms adjusted for mean age 60. Exercise type are ranked according to MD compared to CON. Treatments crossing the y-axis are not significantly different from CON. *MD* Mean Difference, *RT-L* Resistance training of low-moderate load, *AT-M* Aerobic training of moderate intensity, *AT-M+RT-L* Combined moderate intensity aerobic exercise with low to moderate load resistance training, *AT-V* Aerobic training of vigorous intensity, *RT-H* Resistance training of high load.

10.3.5 Exercise frequency

When the model was adjusted for centering value of exercise frequency 3(times/week), the hierarchy from the unadjusted model retained.

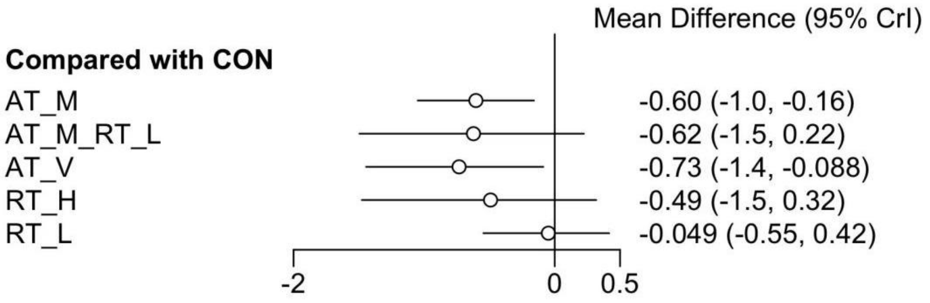

Figure 10.3.5: Forest plot overall change in general symptoms adjusted for exercise frequency 3(times/week). Exercise type are ranked according to MD compared to CON. Treatments crossing the y-axis are not significantly different from CON. *MD* Mean Difference, *RT-L* Resistance training of low-moderate load, *AT-M* Aerobic training of moderate intensity, *AT-M+RT-L* Combined moderate intensity aerobic exercise with low to moderate load resistance training, *AT-V* Aerobic training of vigorous intensity, *RT-H* Resistance training of high load.

10.3.6 Exercise period

When the model was adjusted for exercise period 39(weeks), the hierarchy from the unadjusted model retained.

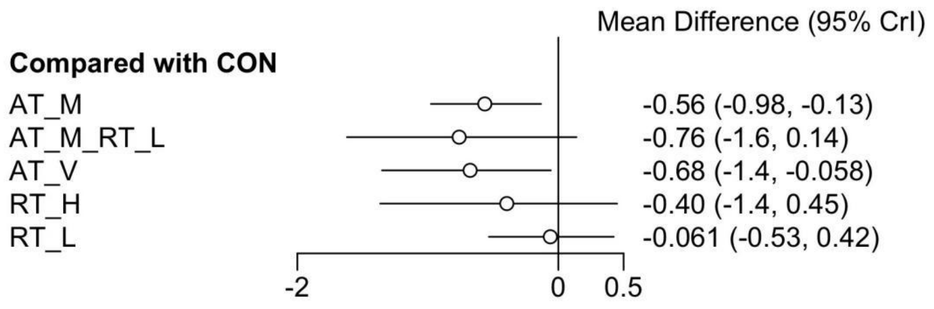

Figure 10.3.6: Forest plot overall change in general symptoms adjusted for exercise period 39(weeks). Exercise type are ranked according to MD compared to CON. Treatments crossing the y-axis are not significantly different from CON. *MD* Mean Difference, *RT-L* Resistance training of low-moderate load, *AT-M* Aerobic training of moderate intensity, *AT-M+RT-L* Combined moderate intensity aerobic exercise with low to moderate load resistance training, *AT-V* Aerobic training of vigorous intensity, *RT-H* Resistance training of high load.

10.3.7 Time of single session

When the model was adjusted for centering value of time of single session 55 minutes, the hierarchy from the unadjusted model retained.

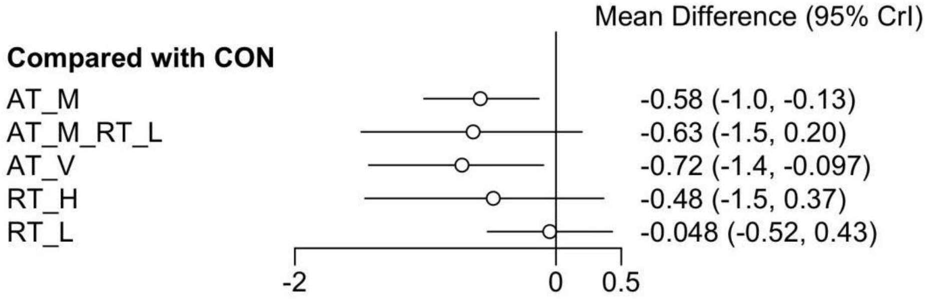

Figure 10.3.7: Forest plot overall change in general symptoms adjusted for time of single session 55 minutes. Exercise type are ranked according to MD compared to CON. Treatments crossing the y-axis are not significantly different from CON. *MD* Mean Difference, *RT-L* Resistance training of low-moderate load, *AT-M* Aerobic training of moderate intensity, *AT-M+RT-L* Combined moderate intensity aerobic exercise with low to moderate load resistance training, *AT-V* Aerobic training of vigorous intensity, *RT-H* Resistance training of high load.

10.4 HbA1c

10.4.1 Publication year

When the model was adjusted for centering value of publish year 2016, the hierarchy from the unadjusted model retained.

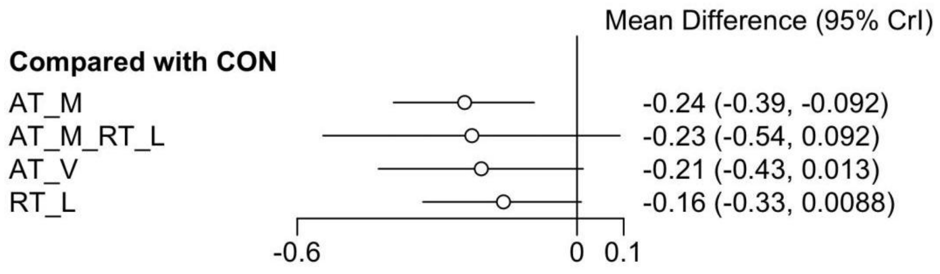

Figure 10.4.1: Forest plot overall change in general symptoms adjusted for publish year 2016. Exercise type are ranked according to MD compared to CON. Treatments crossing the y-axis are not significantly different from CON. *MD* Mean Difference, *RT-L* Resistance training of low-moderate load, *AT-M* Aerobic training of moderate intensity, *AT-M+RT-L* Combined moderate intensity aerobic exercise with low to moderate load resistance training, *AT-V* Aerobic training of vigorous intensity.

10.4.2 Sample size

When the model was adjusted for centering value of sample size 92, hierarchy from the unadjusted model retained.

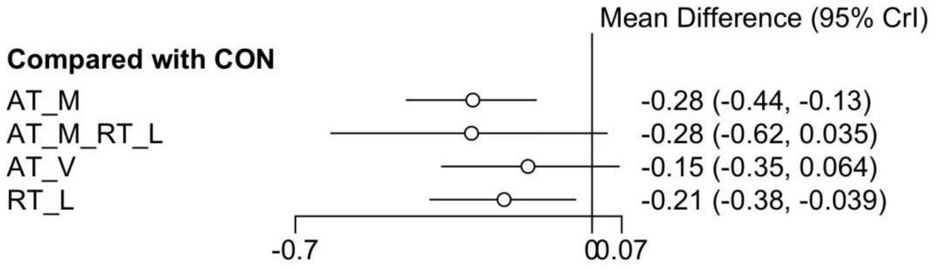

Figure 10.4.2: Forest plot overall change in general symptoms adjusted for sample size 92. Exercise type are ranked according to MD compared to CON. Treatments crossing the y-axis are not significantly different from CON. *MD* Mean Difference, *RT-L* Resistance training of low-moderate load, *AT-M* Aerobic training of moderate intensity, *AT-M+RT-L* Combined moderate intensity aerobic exercise with low to moderate load resistance training, *AT-V* Aerobic training of vigorous intensity.

10.4.3 Percentage of male

When the model was adjusted for centering value of male's percentage 41%, the hierarchy from the unadjusted model retained.

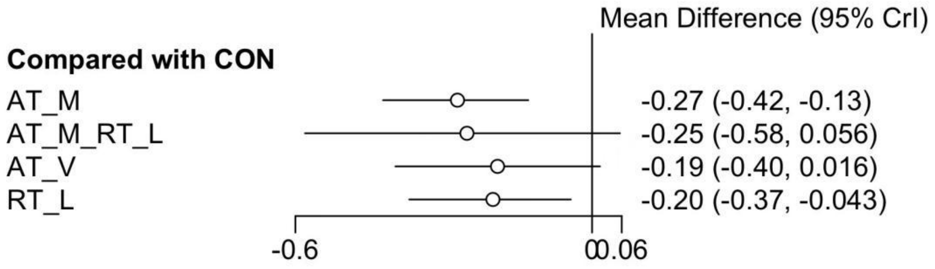

Figure 10.4.3: Forest plot overall change in general symptoms adjusted for male's percentage 41%. Exercise type are ranked according to MD compared to CON. Treatments crossing the y-axis are not significantly different from CON. *MD* Mean Difference, *RT-L* Resistance training of low-moderate load, *AT-M* Aerobic training of moderate intensity, *AT-M+RT-L* Combined moderate intensity aerobic exercise with low to moderate load resistance training, *AT-V* Aerobic training of vigorous intensity.

10.4.4 Mean age

When the model was adjusted for centering value of mean age 59, the hierarchy from the unadjusted model retained.

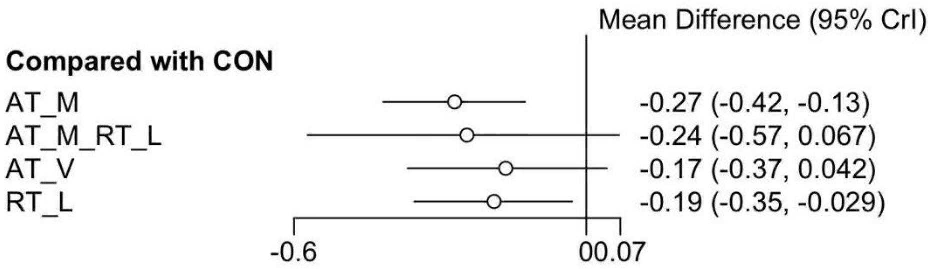

Figure 10.4.4: Forest plot overall change in general symptoms adjusted for mean age 59. Exercise type are ranked according to MD compared to CON. Treatments crossing the y-axis are not significantly different from CON. *MD* Mean Difference, *RT-L* Resistance training of low-moderate load, *AT-M* Aerobic training of moderate intensity, *AT-M+RT-L* Combined moderate intensity aerobic exercise with low to moderate load resistance training, *AT-V* Aerobic training of vigorous intensity.

10.4.5 Exercise frequency

When the model was adjusted for centering value of exercise frequency 3(times/week), the hierarchy from the unadjusted model retained.

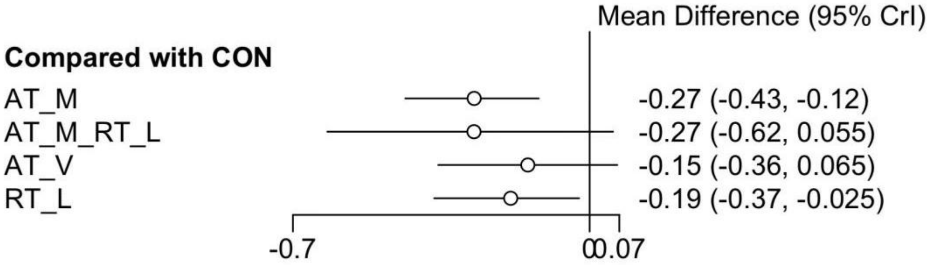

Figure 10.4.5: Forest plot overall change in general symptoms adjusted for exercise frequency 3(times/week). Exercise type are ranked according to MD compared to CON. Treatments crossing the y-axis are not significantly different from CON. *MD* Mean Difference, *RT-L* Resistance training of low-moderate load, *AT-M* Aerobic training of moderate intensity, *AT-M+RT-L* Combined moderate intensity aerobic exercise with low to moderate load resistance training, *AT-V* Aerobic training of vigorous intensity.

10.4.6 Exercise period

When the model was adjusted for exercise period 44(weeks), the hierarchy from the unadjusted model retained.

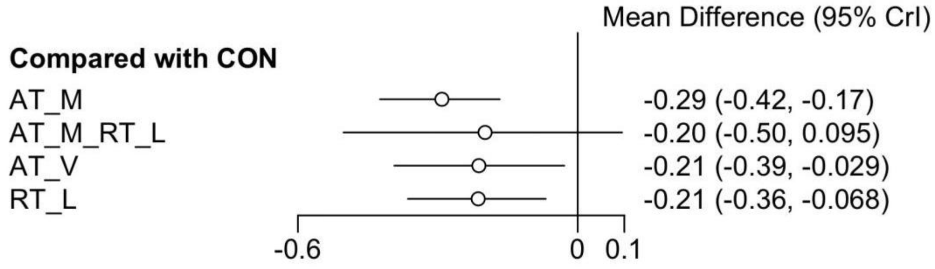

Figure 10.4.6: Forest plot overall change in general symptoms adjusted for exercise period 44(weeks). Exercise type are ranked according to MD compared to CON. Treatments crossing the y-axis are not significantly different from CON. *MD* Mean Difference, *RT-L* Resistance training of low-moderate load, *AT-M* Aerobic training of moderate intensity, *AT-M+RT-L* Combined moderate intensity aerobic exercise with low to moderate load resistance training, *AT-V* Aerobic training of vigorous intensity.

10.4.7 Time of single session

When the model was adjusted for centering value of time of single session 56 minutes, the hierarchy from the unadjusted model retained.

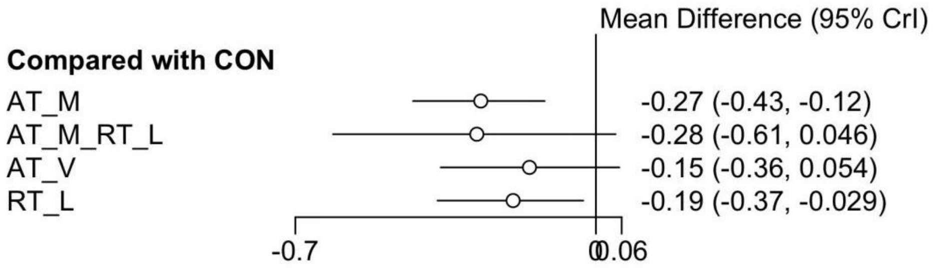

Figure 10.4.7: Forest plot overall change in general symptoms adjusted for time of single session 56 minutes. Exercise type are ranked according to MD compared to CON. Treatments crossing the y-axis are not significantly different from CON. *MD* Mean Difference, *RT-L* Resistance training of low-moderate load, *AT-M* Aerobic training of moderate intensity, *AT-M+RT-L* Combined moderate intensity aerobic exercise with low to moderate load resistance training, *AT-V* Aerobic training of vigorous intensity.

10.5 BMI

10.5.1 Publication year

When the model was adjusted for centering value of publish year 2013, the hierarchy from the unadjusted model retained.

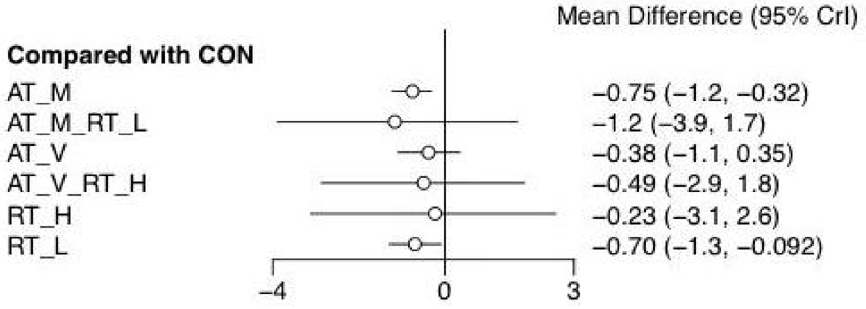

Figure 10.5.1: Forest plot overall change in general symptoms adjusted for time of publish year 2013. Exercise type are ranked according to MD compared to CON. Treatments crossing the y-axis are not significantly different from CON. *MD* Mean Difference, *RT-L* Resistance training of low-moderate load, *AT-M* Aerobic training of moderate intensity, *AT-M+RT-L* Combined moderate intensity aerobic exercise with low to moderate load resistance training, *AT-V* Aerobic training of vigorous intensity, *AT-V+RT-H* Combined vigorous intensity aerobic exercise with high load resistance training, *RT-H* Resistance training of high load.

10.5.2 Sample size

When the model was adjusted for centering value of sample size 78, hierarchy from the unadjusted model retained.

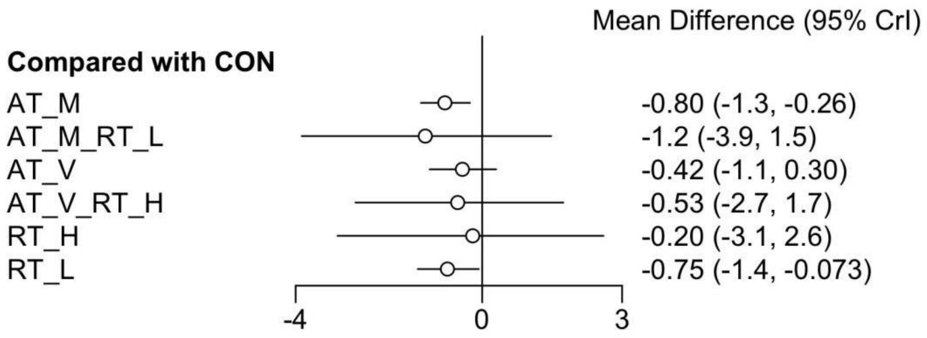

Figure 10.5.2: Forest plot overall change in general symptoms adjusted for time of sample size 78. Exercise type are ranked according to MD compared to CON. Treatments crossing the y-axis are not significantly different from CON. *MD* Mean Difference, *RT-L* Resistance training of low-moderate load, *AT-M* Aerobic training of moderate intensity, *AT-M+RT-L* Combined moderate intensity aerobic exercise with low to moderate load resistance training, *AT-V* Aerobic training of vigorous intensity, *AT-V+RT-H* Combined vigorous intensity aerobic exercise with high load resistance training, *RT-H* Resistance training of high load.

10.5.3 Percentage of male

When the model was adjusted for centering value of male's percentage 35%, hierarchy from the unadjusted model retained.

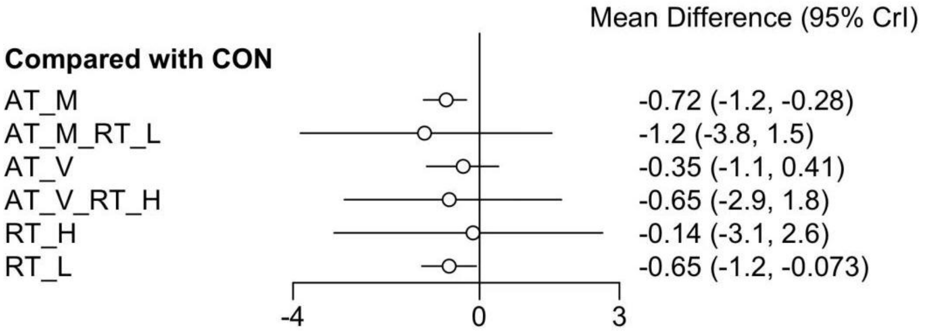

Figure 10.5.3: Forest plot overall change in general symptoms adjusted for time of male's percentage 35%. Exercise type are ranked according to MD compared to CON. Treatments crossing the y-axis are not significantly different from CON. *MD* Mean Difference, *RT-L* Resistance training of low-moderate load, *AT-M* Aerobic training of moderate intensity, *AT-M+RT-L* Combined moderate intensity aerobic exercise with low to moderate load resistance training, *AT-V* Aerobic training of vigorous intensity, *AT-V+RT-H* Combined vigorous intensity aerobic exercise with high load resistance training, *RT-H* Resistance training of high load.

10.5.4 Mean age

When the model was adjusted for centering value of mean age 59, the hierarchy from the unadjusted model retained.

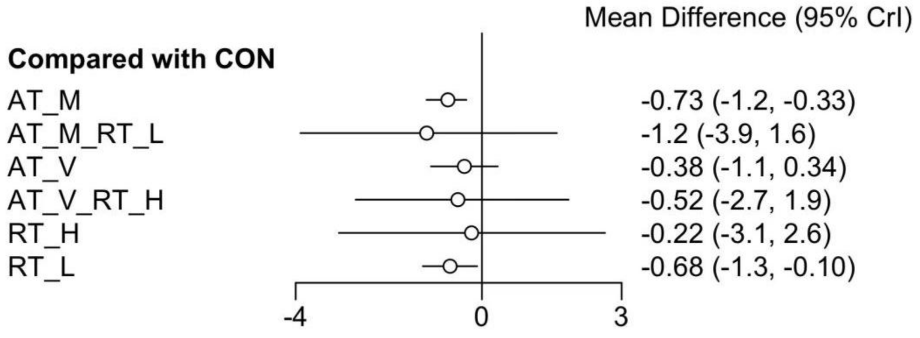

Figure 10.5.4: Forest plot overall change in general symptoms adjusted for time of mean age 59. Exercise type are ranked according to MD compared to CON. Treatments crossing the y-axis are not significantly different from CON. *MD* Mean Difference, *RT-L* Resistance training of low-moderate load, *AT-M* Aerobic training of moderate intensity, *AT-M+RT-L* Combined moderate intensity aerobic exercise with low to moderate load resistance training, *AT-V* Aerobic training of vigorous intensity, *AT-V+RT-H* Combined vigorous intensity aerobic exercise with high load resistance training, *RT-H* Resistance training of high load.

10.5.5 Exercise frequency

When the model was adjusted for centering value of exercise frequency 3(times/week), the hierarchy from the unadjusted model retained.

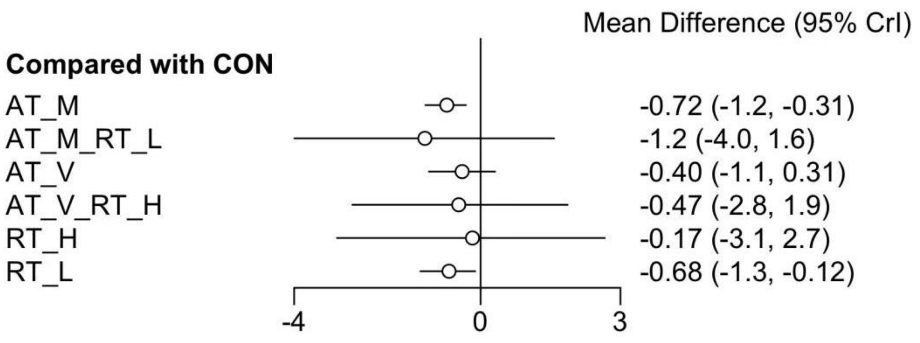

Figure 10.5.5: Forest plot overall change in general symptoms adjusted for time of exercise frequency 3(times/week). Exercise type are ranked according to MD compared to CON. Treatments crossing the y-axis are not significantly different from CON. *MD* Mean Difference, *RT-L* Resistance training of low-moderate load, *AT-M* Aerobic training of moderate intensity, *AT-M+RT-L* Combined moderate intensity aerobic exercise with low to moderate load resistance training, *AT-V* Aerobic training of vigorous intensity, *AT-V+RT-H* Combined vigorous intensity aerobic exercise with high load resistance training, *RT-H* Resistance training of high load.

10.5.6 Exercise period

When the model was adjusted for centering value of exercise period 22(weeks), the hierarchy from the unadjusted model retained.

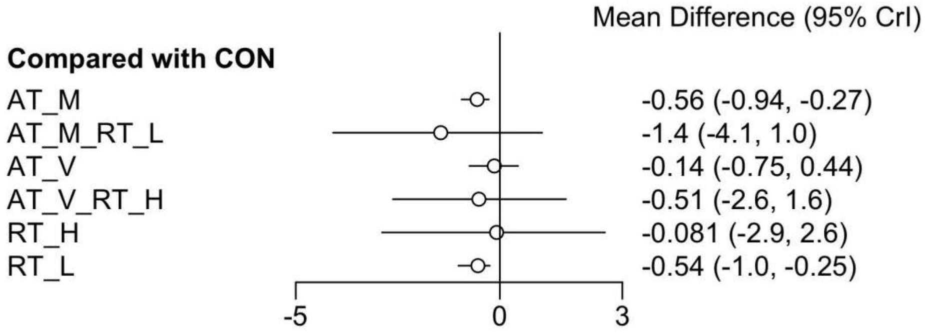

Figure 10.5.6: Forest plot overall change in general symptoms adjusted for time of exercise period 22(weeks). Exercise type are ranked according to MD compared to CON. Treatments crossing the y-axis are not significantly different from CON. *MD* Mean Difference, *RT-L* Resistance training of low-moderate load, *AT-M* Aerobic training of moderate intensity, *AT-M+RT-L* Combined moderate intensity aerobic exercise with low to moderate load resistance training, *AT-V* Aerobic training of vigorous intensity, *AT-V+RT-H* Combined vigorous intensity aerobic exercise with high load resistance training, *RT-H* Resistance training of high load.

10.5.7 Time of single session

When the model was adjusted for centering value of time of single session 51 minutes, the hierarchy from the unadjusted model retained.

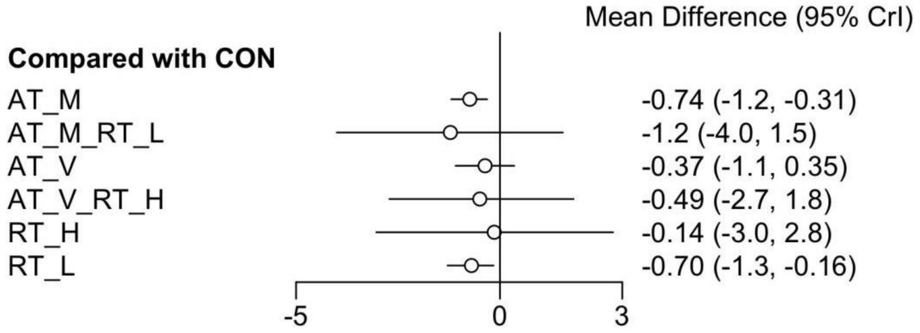

Figure 10.5.7: Forest plot overall change in general symptoms adjusted for time of single session 51 minutes. Exercise type are ranked according to MD compared to CON. Treatments crossing the y-axis are not significantly different from CON. *MD* Mean Difference, *RT-L* Resistance training of low-moderate load, *AT-M* Aerobic training of moderate intensity, *AT-M+RT-L* Combined moderate intensity aerobic exercise with low to moderate load resistance training, *AT-V* Aerobic training of vigorous intensity, *AT-V+RT-H* Combined vigorous intensity aerobic exercise with high load resistance training, *RT-H* Resistance training of high load.

10.6 Weight

10.6.1 Publication year

When the model was adjusted for centering value of publish year 2014, the hierarchy from the unadjusted model retained.

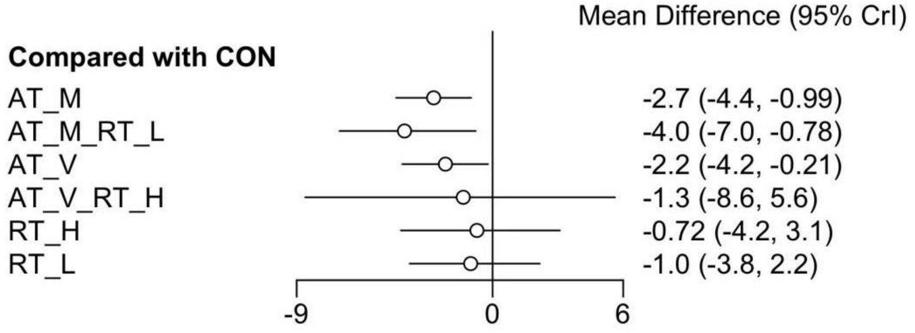

Figure 10.6.1: Forest plot overall change in general symptoms adjusted for time of publish year 2014. Exercise type are ranked according to MD compared to CON. Treatments crossing the y-axis are not significantly different from CON. *MD* Mean Difference, *RT-L* Resistance training of low-moderate load, *AT-M* Aerobic training of moderate intensity, *AT-M+RT-L* Combined moderate intensity aerobic exercise with low to moderate load resistance training, *AT-V* Aerobic training of vigorous intensity, *AT-V+RT-H* Combined vigorous intensity aerobic exercise with high load resistance training, *RT-H* Resistance training of high load.

10.6.2 Sample size

When the model was adjusted for centering value of sample size 78, hierarchy from the unadjusted model retained.

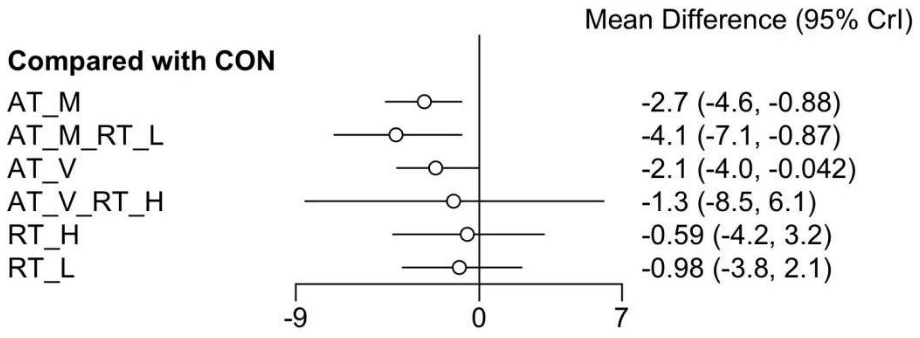

Figure 10.6.2: Forest plot overall change in general symptoms adjusted for time of sample size 78. Exercise type are ranked according to MD compared to CON. Treatments crossing the y-axis are not significantly different from CON. *MD* Mean Difference, *RT-L* Resistance training of low-moderate load, *AT-M* Aerobic training of moderate intensity, *AT-M+RT-L* Combined moderate intensity aerobic exercise with low to moderate load resistance training, *AT-V* Aerobic training of vigorous intensity, *AT-V+RT-H* Combined vigorous intensity aerobic exercise with high load resistance training, *RT-H* Resistance training of high load.

10.6.3 Percentage of male

When the model was adjusted for centering value of male's percentage 32%, hierarchy from the unadjusted model retained.

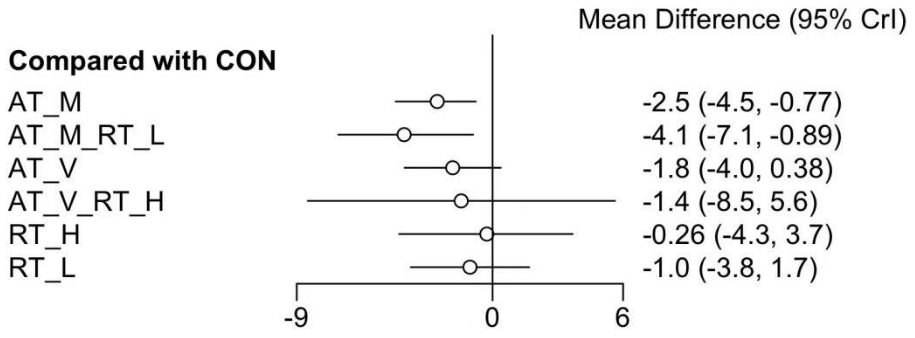

Figure 10.5.3: Forest plot overall change in general symptoms adjusted for time of male's percentage 32%. Exercise type are ranked according to MD compared to CON. Treatments crossing the y-axis are not significantly different from CON. *MD* Mean Difference, *RT-L* Resistance training of low-moderate load, *AT-M* Aerobic training of moderate intensity, *AT-M+RT-L* Combined moderate intensity aerobic exercise with low to moderate load resistance training, *AT-V* Aerobic training of vigorous intensity, *AT-V+RT-H* Combined vigorous intensity aerobic exercise with high load resistance training, *RT-H* Resistance training of high load.

10.6.4 Mean age

When the model was adjusted for centering value of mean age 59, the hierarchy from the unadjusted model retained.

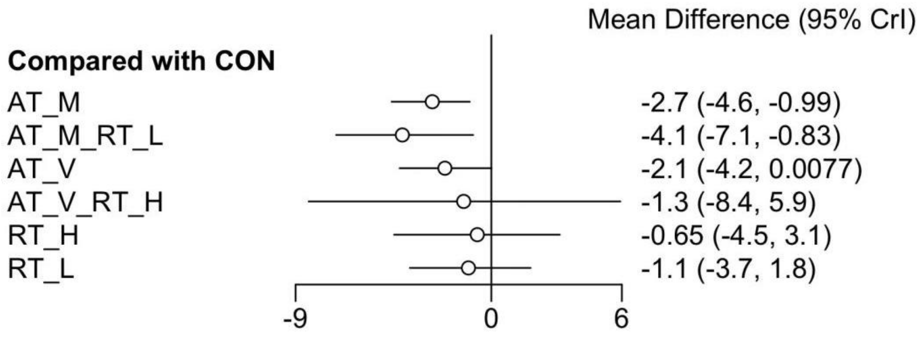

Figure 10.5.4: Forest plot overall change in general symptoms adjusted for time of mean age 59. Exercise type are ranked according to MD compared to CON. Treatments crossing the y-axis are not significantly different from CON. *MD* Mean Difference, *RT-L* Resistance training of low-moderate load, *AT-M* Aerobic training of moderate intensity, *AT-M+RT-L* Combined moderate intensity aerobic exercise with low to moderate load resistance training, *AT-V* Aerobic training of vigorous intensity, *AT-V+RT-H* Combined vigorous intensity aerobic exercise with high load resistance training, *RT-H* Resistance training of high load.

10.6.5 Exercise frequency

When the model was adjusted for centering value of exercise frequency 3(times/week), the hierarchy from the unadjusted model retained.

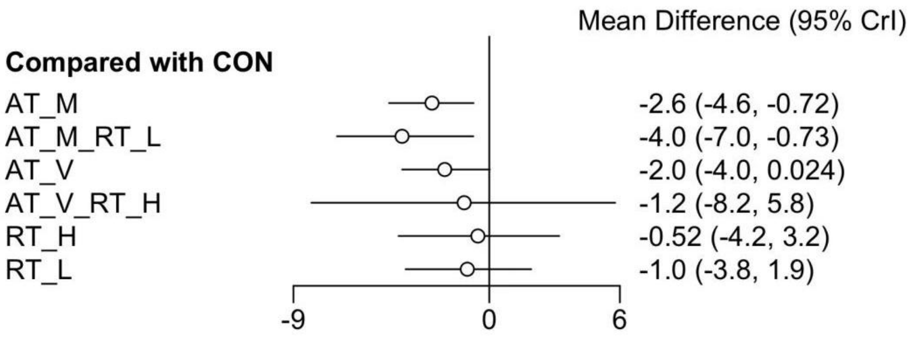

Figure 10.6.5: Forest plot overall change in general symptoms adjusted for time of exercise frequency 3(times/week). Exercise type are ranked according to MD compared to CON. Treatments crossing the y-axis are not significantly different from CON. *MD* Mean Difference, *RT-L* Resistance training of low-moderate load, *AT-M* Aerobic training of moderate intensity, *AT-M+RT-L* Combined moderate intensity aerobic exercise with low to moderate load resistance training, *AT-V* Aerobic training of vigorous intensity, *AT-V+RT-H* Combined vigorous intensity aerobic exercise with high load resistance training, *RT-H* Resistance training of high load.

10.6.6 Exercise period

When the model was adjusted for centering value of exercise period 37(weeks), the hierarchy from the unadjusted model retained.

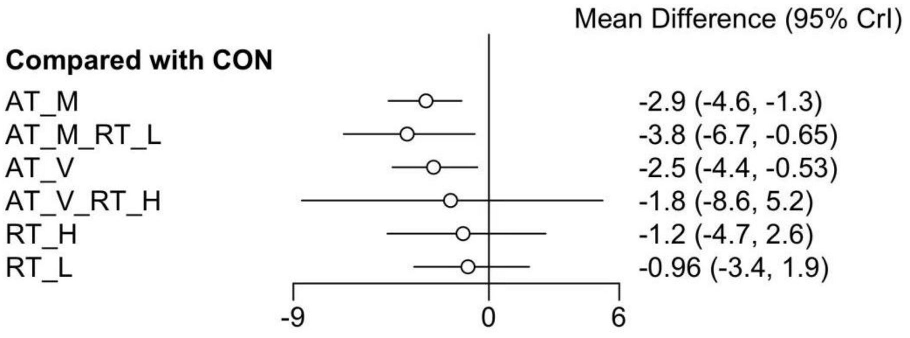

Figure 10.6.6: Forest plot overall change in general symptoms adjusted for time of exercise period 37(weeks). Exercise type are ranked according to MD compared to CON. Treatments crossing the y-axis are not significantly different from CON. *MD* Mean Difference, *RT-L* Resistance training of low-moderate load, *AT-M* Aerobic training of moderate intensity, *AT-M+RT-L* Combined moderate intensity aerobic exercise with low to moderate load resistance training, *AT-V* Aerobic training of vigorous intensity, *AT-V+RT-H* Combined vigorous intensity aerobic exercise with high load resistance training, *RT-H* Resistance training of high load.

10.6.7 Time of single session

When the model was adjusted for centering value of time of single session 51 minutes, the hierarchy from the unadjusted model retained.

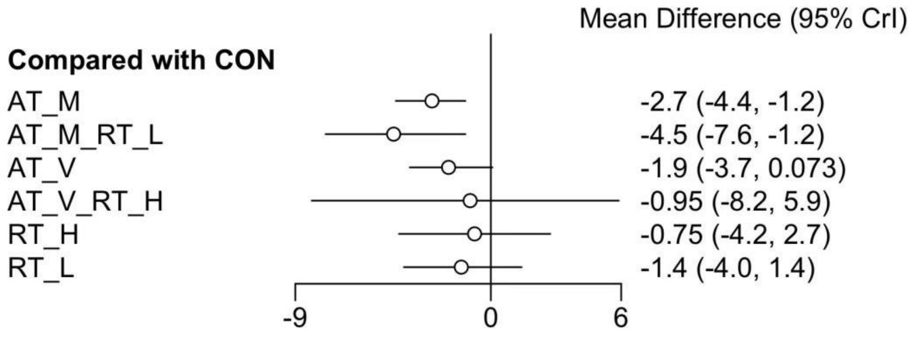

Figure 10.6.7: Forest plot overall change in general symptoms adjusted for time of single session 51 minutes. Exercise type are ranked according to MD compared to CON. Treatments crossing the y-axis are not significantly different from CON. *MD* Mean Difference, *RT-L* Resistance training of low-moderate load, *AT-M* Aerobic training of moderate intensity, *AT-M+RT-L* Combined moderate intensity aerobic exercise with low to moderate load resistance training, *AT-V* Aerobic training of vigorous intensity, *AT-V+RT-H* Combined vigorous intensity aerobic exercise with high load resistance training, *RT-H* Resistance training of high load.

10.7 TC

10.7.1 Publication year

When the model was adjusted for centering value of publish year 2014, the hierarchy from the unadjusted model retained.

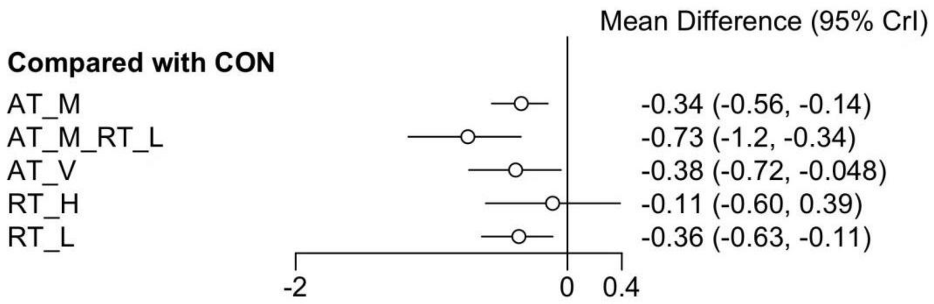

Figure 10.7.1: Forest plot overall change in general symptoms adjusted for time of publish year 2014. Exercise type are ranked according to MD compared to CON. Treatments crossing the y-axis are not significantly different from CON. *MD* Mean Difference, *RT-L* Resistance training of low-moderate load, *AT-M* Aerobic training of moderate intensity, *AT-M+RT-L* Combined moderate intensity aerobic exercise with low to moderate load resistance training, *AT-V* Aerobic training of vigorous intensity, *RT-H* Resistance training of high load.

10.7.2 Sample size

When the model was adjusted for centering value of sample size 92, hierarchy from the unadjusted model retained.

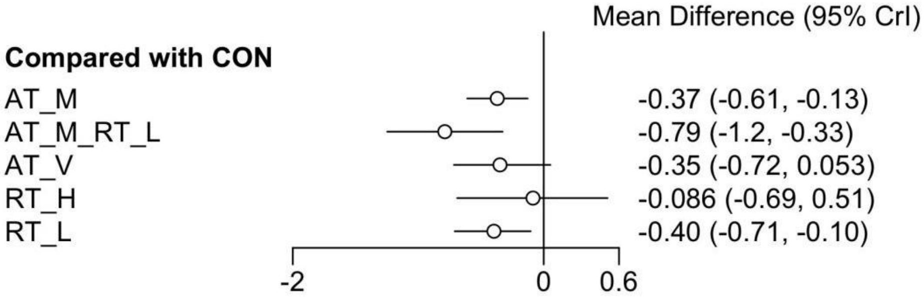

Figure 10.7.2: Forest plot overall change in general symptoms adjusted for time of sample size 92. Exercise type are ranked according to MD compared to CON. Treatments crossing the y-axis are not significantly different from CON. *MD* Mean Difference, *RT-L* Resistance training of low-moderate load, *AT-M* Aerobic training of moderate intensity, *AT-M+RT-L* Combined moderate intensity aerobic exercise with low to moderate load resistance training, *AT-V* Aerobic training of vigorous intensity, *RT-H* Resistance training of high load.

10.7.3 Percentage of male

When the model was adjusted for centering value of male's percentage 39%, hierarchy from the unadjusted model retained.

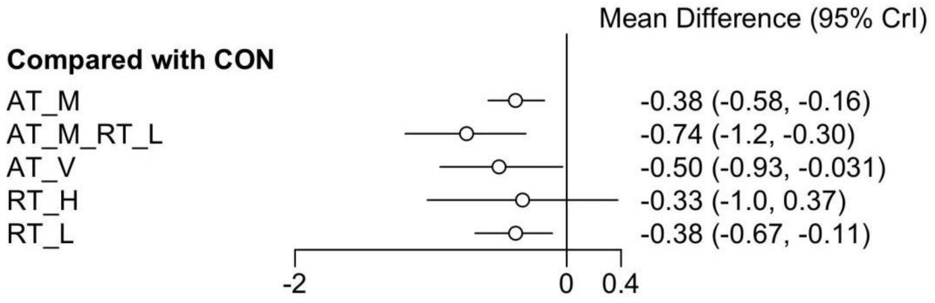

Figure 10.7.3: Forest plot overall change in general symptoms adjusted for time of male's percentage 39%. Exercise type are ranked according to MD compared to CON. Treatments crossing the y-axis are not significantly different from CON. *MD* Mean Difference, *RT-L* Resistance training of low-moderate load, *AT-M* Aerobic training of moderate intensity, *AT-M+RT-L* Combined moderate intensity aerobic exercise with low to moderate load resistance training, *AT-V* Aerobic training of vigorous intensity, *RT-H* Resistance training of high load.

10.7.4 Mean age

When the model was adjusted for centering value of mean age 60, the hierarchy from the unadjusted model retained.

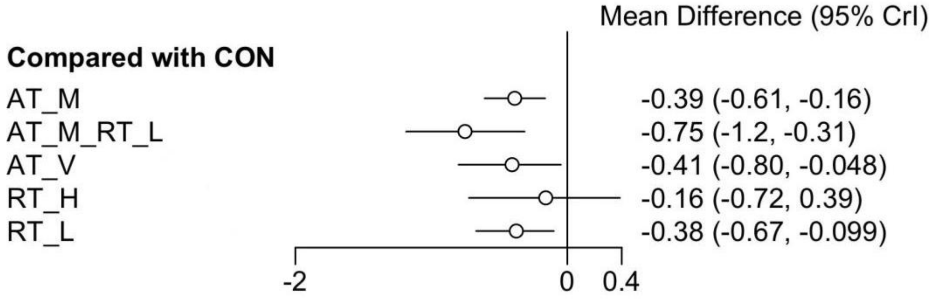

Figure 10.7.4: Forest plot overall change in general symptoms adjusted for time of mean age 60. Exercise type are ranked according to MD compared to CON. Treatments crossing the y-axis are not significantly different from CON. *MD* Mean Difference, *RT-L* Resistance training of low-moderate load, *AT-M* Aerobic training of moderate intensity, *AT-M+RT-L* Combined moderate intensity aerobic exercise with low to moderate load resistance training, *AT-V* Aerobic training of vigorous intensity, *RT-H* Resistance training of high load.

10.7.5 Exercise frequency

When the model was adjusted for centering value of exercise frequency 3(times/week), the hierarchy from the unadjusted model retained.

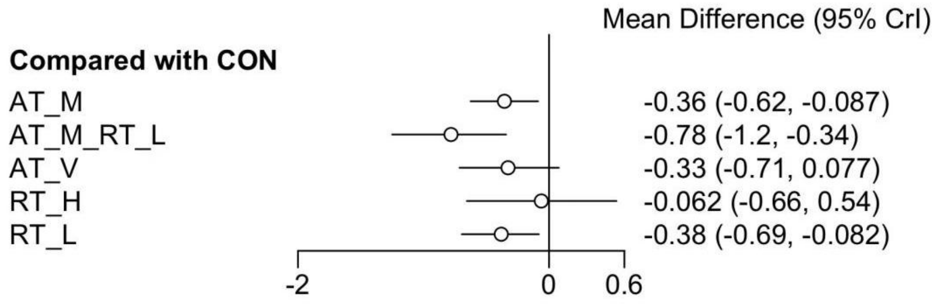

Figure 10.7.5: Forest plot overall change in general symptoms adjusted for time of exercise frequency 3(times/week). Exercise type are ranked according to MD compared to CON. Treatments crossing the y-axis are not significantly different from CON. *MD* Mean Difference, *RT-L* Resistance training of low-moderate load, *AT-M* Aerobic training of moderate intensity, *AT-M+RT-L* Combined moderate intensity aerobic exercise with low to moderate load resistance training, *AT-V* Aerobic training of vigorous intensity, *RT-H* Resistance training of high load.

10.7.6 Exercise period

When the model was adjusted for centering value of exercise period 42(weeks), the hierarchy from the unadjusted model retained.

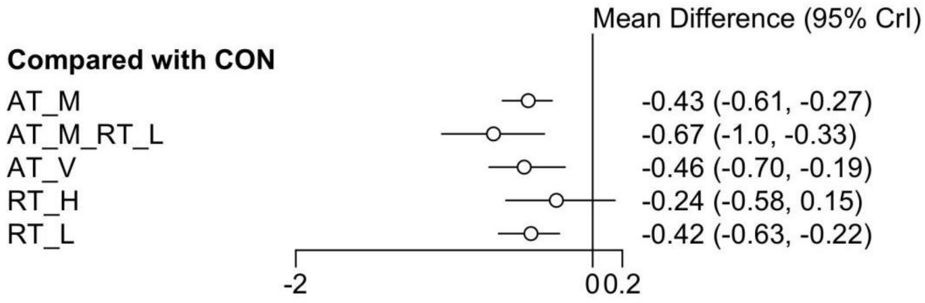

Figure 10.7.6: Forest plot overall change in general symptoms adjusted for time of exercise period 42(weeks). Exercise type are ranked according to MD compared to CON. Treatments crossing the y-axis are not significantly different from CON. *MD* Mean Difference, *RT-L* Resistance training of low-moderate load, *AT-M* Aerobic training of moderate intensity, *AT-M+RT-L* Combined moderate intensity aerobic exercise with low to moderate load resistance training, *AT-V* Aerobic training of vigorous intensity, *RT-H* Resistance training of high load.

10.7.7 Time of single session

When the model was adjusted for centering value of time of single session 52 minutes, the hierarchy from the unadjusted model retained.

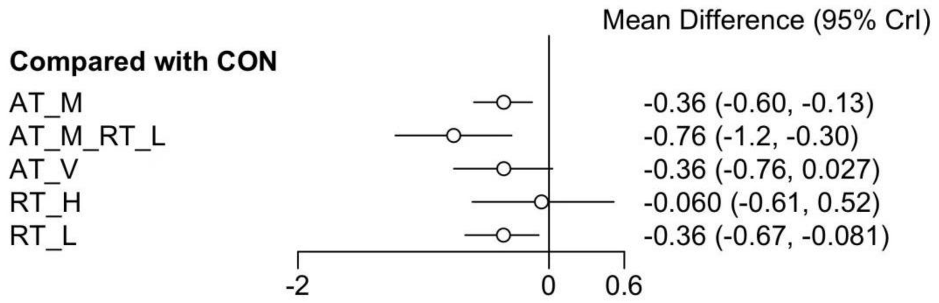

Figure 10.7.7: Forest plot overall change in general symptoms adjusted for time of single session 52 minutes. Exercise type are ranked according to MD compared to CON. Treatments crossing the y-axis are not significantly different from CON. *MD* Mean Difference, *RT-L* Resistance training of low-moderate load, *AT-M* Aerobic training of moderate intensity, *AT-M+RT-L* Combined moderate intensity aerobic exercise with low to moderate load resistance training, *AT-V* Aerobic training of vigorous intensity, *RT-H* Resistance training of high load.

10.8 SBP

10.8.1 Publication year

When the model was adjusted for centering value of publish year 2013, the hierarchy from the unadjusted model retained.

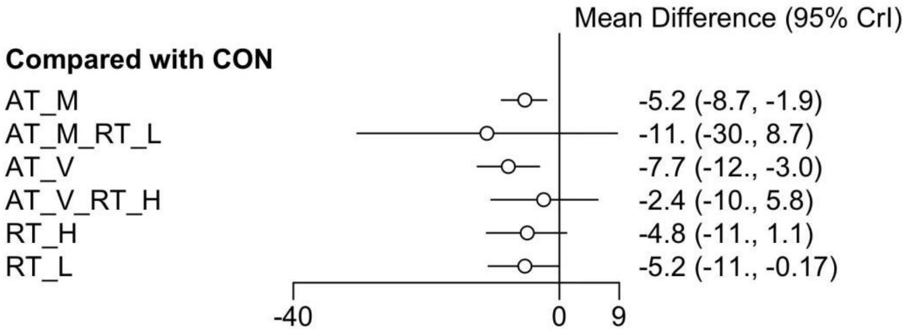

Figure 10.8.1: Forest plot overall change in general symptoms adjusted for time of publish year 2013. Exercise type are ranked according to MD compared to CON. Treatments crossing the y-axis are not significantly different from CON. *MD* Mean Difference, *RT-L* Resistance training of low-moderate load, *AT-M* Aerobic training of moderate intensity, *AT-M+RT-L* Combined moderate intensity aerobic exercise with low to moderate load resistance training, *AT-V* Aerobic training of vigorous intensity, *AT-V+RT-H* Combined vigorous intensity aerobic exercise with high load resistance training, *RT-H* Resistance training of high load.

10.8.2 Sample size

When the model was adjusted for centering value of sample size 85, hierarchy from the unadjusted model retained.

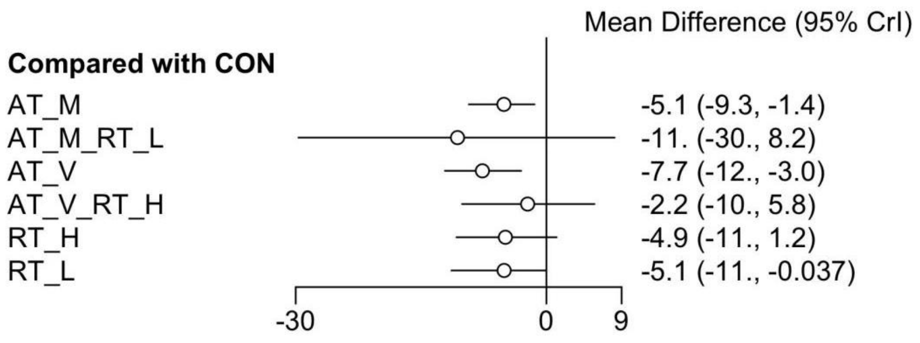

Figure 10.8.2: Forest plot overall change in general symptoms adjusted for time of sample size 85. Exercise type are ranked according to MD compared to CON. Treatments crossing the y-axis are not significantly different from CON. *MD* Mean Difference, *RT-L* Resistance training of low-moderate load, *AT-M* Aerobic training of moderate intensity, *AT-M+RT-L* Combined moderate intensity aerobic exercise with low to moderate load resistance training, *AT-V* Aerobic training of vigorous intensity, *AT-V+RT-H* Combined vigorous intensity aerobic exercise with high load resistance training, *RT-H* Resistance training of high load.

10.8.3 Percentage of male

When the model was adjusted for centering value of male's percentage 36%, hierarchy from the unadjusted model retained.

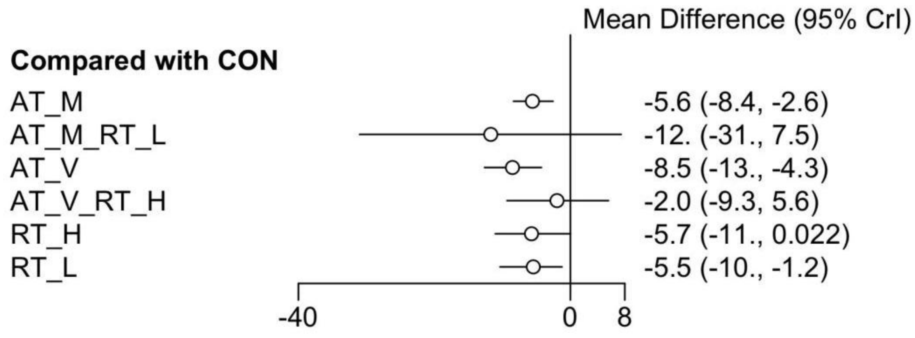

Figure 10.8.3: Forest plot overall change in general symptoms adjusted for time of male's percentage 36%. Exercise type are ranked according to MD compared to CON. Treatments crossing the y-axis are not significantly different from CON. *MD* Mean Difference, *RT-L* Resistance training of low-moderate load, *AT-M* Aerobic training of moderate intensity, *AT-M+RT-L* Combined moderate intensity aerobic exercise with low to moderate load resistance training, *AT-V* Aerobic training of vigorous intensity, *AT-V+RT-H* Combined vigorous intensity aerobic exercise with high load resistance training, *RT-H* Resistance training of high load.

10.8.4 Mean age

When the model was adjusted for centering value of mean age 58, the hierarchy from the unadjusted model retained.

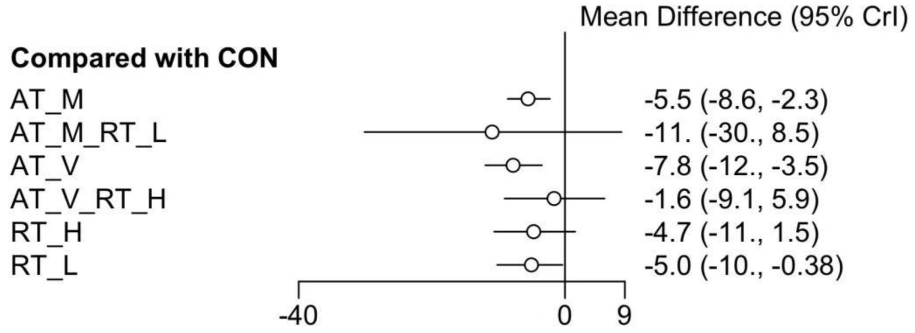

Figure 10.8.4: Forest plot overall change in general symptoms adjusted for time of mean age 58. Exercise type are ranked according to MD compared to CON. Treatments crossing the y-axis are not significantly different from CON. *MD* Mean Difference, *RT-L* Resistance training of low-moderate load, *AT-M* Aerobic training of moderate intensity, *AT-M+RT-L* Combined moderate intensity aerobic exercise with low to moderate load resistance training, *AT-V* Aerobic training of vigorous intensity, *AT-V+RT-H* Combined vigorous intensity aerobic exercise with high load resistance training, *RT-H* Resistance training of high load.

10.8.5 Exercise frequency

When the model was adjusted for centering value of exercise frequency 3(times/week), the hierarchy from the unadjusted model retained.

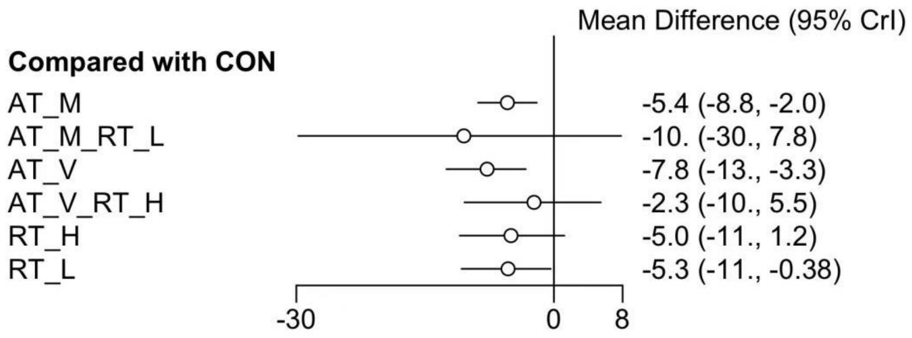

Figure 10.8.5: Forest plot overall change in general symptoms adjusted for time of exercise frequency 3(times/week). Exercise type are ranked according to MD compared to CON. Treatments crossing the y-axis are not significantly different from CON. *MD* Mean Difference, *RT-L* Resistance training of low-moderate load, *AT-M* Aerobic training of moderate intensity, *AT-M+RT-L* Combined moderate intensity aerobic exercise with low to moderate load resistance training, *AT-V* Aerobic training of vigorous intensity, *AT-V+RT-H* Combined vigorous intensity aerobic exercise with high load resistance training, *RT-H* Resistance training of high load.

10.8.6 Exercise period

When the model was adjusted for centering value of exercise period 22(weeks), the hierarchy from the unadjusted model retained.

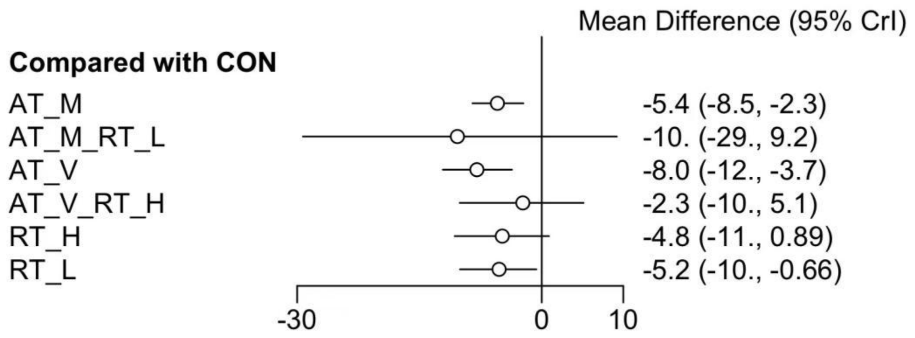

Figure 10.8.6: Forest plot overall change in general symptoms adjusted for time of exercise period 22(weeks). Exercise type are ranked according to MD compared to CON. Treatments crossing the y-axis are not significantly different from CON. *MD* Mean Difference, *RT-L* Resistance training of low-moderate load, *AT-M* Aerobic training of moderate intensity, *AT-M+RT-L* Combined moderate intensity aerobic exercise with low to moderate load resistance training, *AT-V* Aerobic training of vigorous intensity, *AT-V+RT-H* Combined vigorous intensity aerobic exercise with high load resistance training, *RT-H* Resistance training of high load.

10.8.7 Time of single session

When the model was adjusted for centering value of time of single session 52 minutes, the hierarchy from the unadjusted model retained.

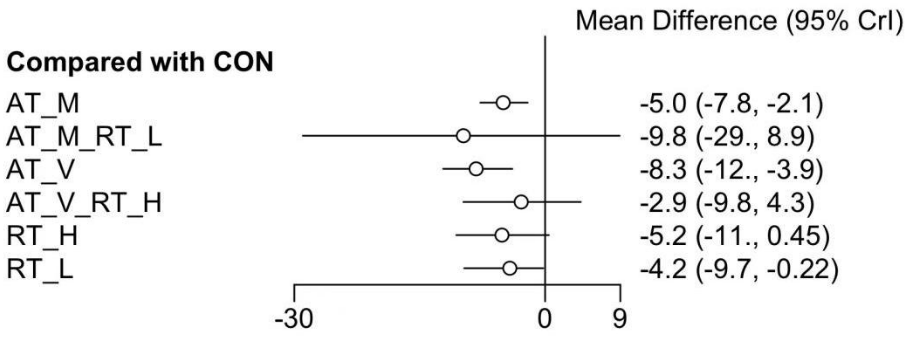

Figure 10.8.7: Forest plot overall change in general symptoms adjusted for time of single session 52 minutes. Exercise type are ranked according to MD compared to CON. Treatments crossing the y-axis are not significantly different from CON. *MD* Mean Difference, *RT-L* Resistance training of low-moderate load, *AT-M* Aerobic training of moderate intensity, *AT-M+RT-L* Combined moderate intensity aerobic exercise with low to moderate load resistance training, *AT-V* Aerobic training of vigorous intensity, *AT-V+RT-H* Combined vigorous intensity aerobic exercise with high load resistance training, *RT-H* Resistance training of high load.

10.9 DBP

10.9.1 Publication year

When the model was adjusted for centering value of publish year 2013, the hierarchy from the unadjusted model retained.

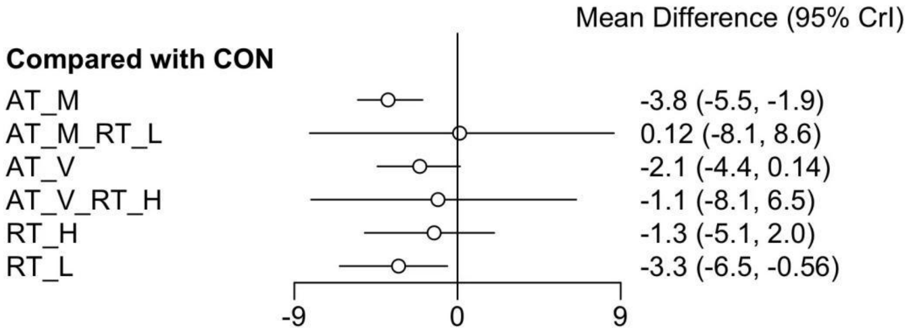

Figure 10.9.1: Forest plot overall change in general symptoms adjusted for time of publish year 2013. Exercise type are ranked according to MD compared to CON. Treatments crossing the y-axis are not significantly different from CON. *MD* Mean Difference, *RT-L* Resistance training of low-moderate load, *AT-M* Aerobic training of moderate intensity, *AT-M+RT-L* Combined moderate intensity aerobic exercise with low to moderate load resistance training, *AT-V* Aerobic training of vigorous intensity, *AT-V+RT-H* Combined vigorous intensity aerobic exercise with high load resistance training, *RT-H* Resistance training of high load.

10.9.2 Sample size

When the model was adjusted for centering value of sample size 85, hierarchy from the unadjusted model retained.

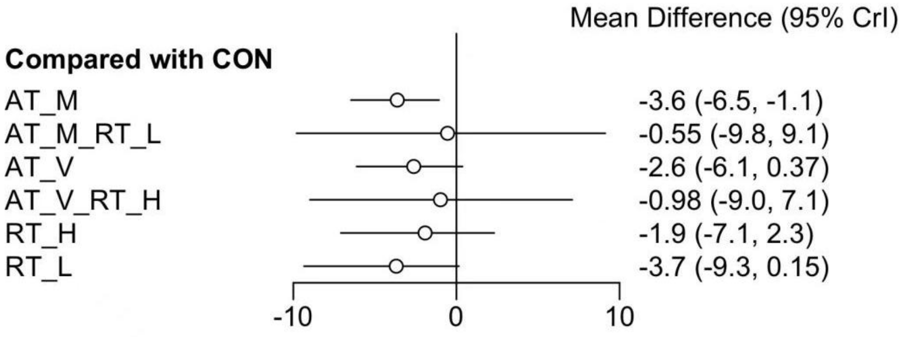

Figure 10.9.2: Forest plot overall change in general symptoms adjusted for time of sample size 85. Exercise type are ranked according to MD compared to CON. Treatments crossing the y-axis are not significantly different from CON. *MD* Mean Difference, *RT-L* Resistance training of low-moderate load, *AT-M* Aerobic training of moderate intensity, *AT-M+RT-L* Combined moderate intensity aerobic exercise with low to moderate load resistance training, *AT-V* Aerobic training of vigorous intensity, *AT-V+RT-H* Combined vigorous intensity aerobic exercise with high load resistance training, *RT-H* Resistance training of high load.

10.9.3 Percentage of male

When the model was adjusted for centering value of male's percentage 36%, hierarchy from the unadjusted model retained.

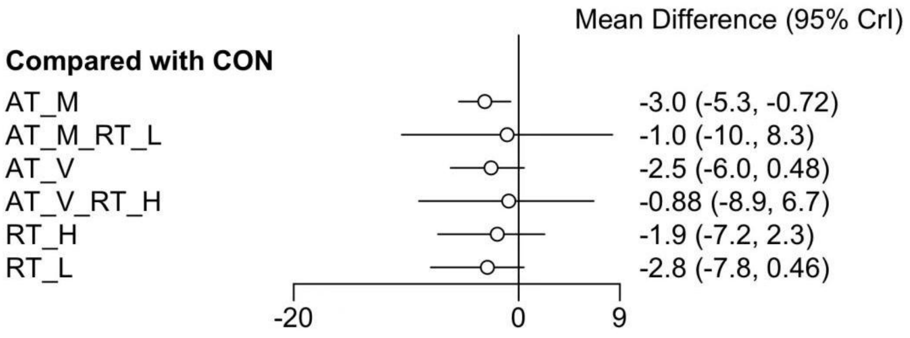

Figure 10.9.3: Forest plot overall change in general symptoms adjusted for time of male's percentage 36%. Exercise type are ranked according to MD compared to CON. Treatments crossing the y-axis are not significantly different from CON. *MD* Mean Difference, *RT-L* Resistance training of low-moderate load, *AT-M* Aerobic training of moderate intensity, *AT-M+RT-L* Combined moderate intensity aerobic exercise with low to moderate load resistance training, *AT-V* Aerobic training of vigorous intensity, *AT-V+RT-H* Combined vigorous intensity aerobic exercise with high load resistance training, *RT-H* Resistance training of high load.

10.9.4 Mean age

When the model was adjusted for centering value of mean age 58, the hierarchy from the unadjusted model retained.

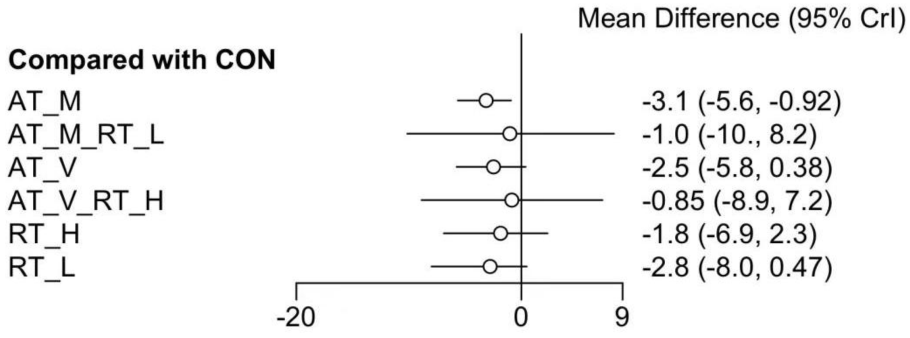

Figure 10.9.4: Forest plot overall change in general symptoms adjusted for time of mean age 58. Exercise type are ranked according to MD compared to CON. Treatments crossing the y-axis are not significantly different from CON. *MD* Mean Difference, *RT-L* Resistance training of low-moderate load, *AT-M* Aerobic training of moderate intensity, *AT-M+RT-L* Combined moderate intensity aerobic exercise with low to moderate load resistance training, *AT-V* Aerobic training of vigorous intensity, *AT-V+RT-H* Combined vigorous intensity aerobic exercise with high load resistance training, *RT-H* Resistance training of high load.

10.9.5 Exercise frequency

When the model was adjusted for centering value of exercise frequency 3(times/week), the hierarchy from the unadjusted model retained.

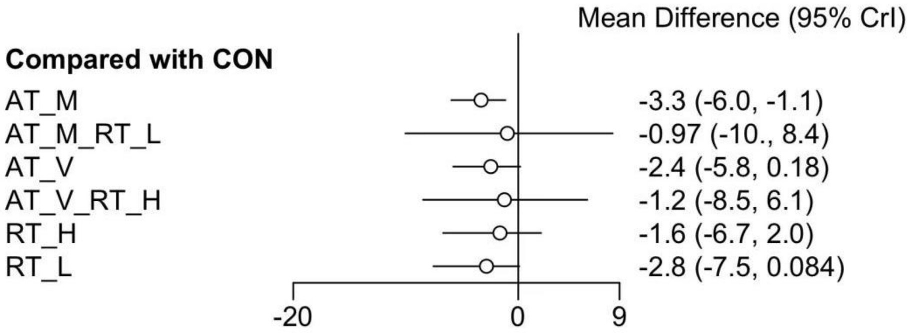

Figure 10.9.5: Forest plot overall change in general symptoms adjusted for time of exercise frequency 3(times/week). Exercise type are ranked according to MD compared to CON. Treatments crossing the y-axis are not significantly different from CON. *MD* Mean Difference, *RT-L* Resistance training of low-moderate load, *AT-M* Aerobic training of moderate intensity, *AT-M+RT-L* Combined moderate intensity aerobic exercise with low to moderate load resistance training, *AT-V* Aerobic training of vigorous intensity, *AT-V+RT-H* Combined vigorous intensity aerobic exercise with high load resistance training, *RT-H* Resistance training of high load.

10.9.6 Exercise period

When the model was adjusted for centering value of exercise period 22(weeks), the hierarchy from the unadjusted model retained.

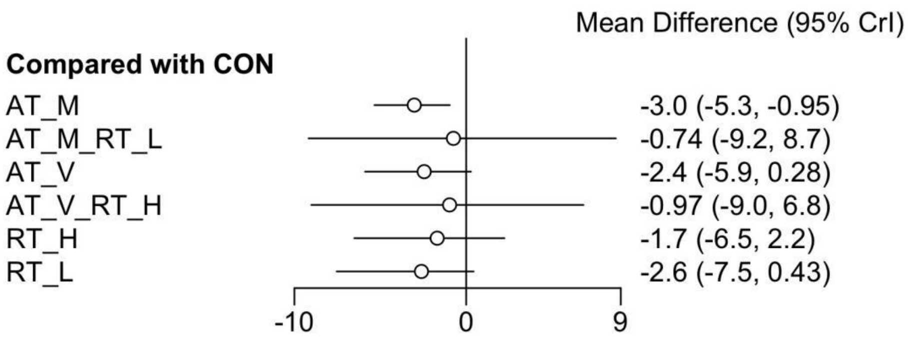

Figure 10.9.6: Forest plot overall change in general symptoms adjusted for time of exercise period 22(weeks). Exercise type are ranked according to MD compared to CON. Treatments crossing the y-axis are not significantly different from CON. *MD* Mean Difference, *RT-L* Resistance training of low-moderate load, *AT-M* Aerobic training of moderate intensity, *AT-M+RT-L* Combined moderate intensity aerobic exercise with low to moderate load resistance training, *AT-V* Aerobic training of vigorous intensity, *AT-V+RT-H* Combined vigorous intensity aerobic exercise with high load resistance training, *RT-H* Resistance training of high load.

10.9.7 Time of single session

When the model was adjusted for centering value of time of single session 50 minutes, the hierarchy from the unadjusted model retained.

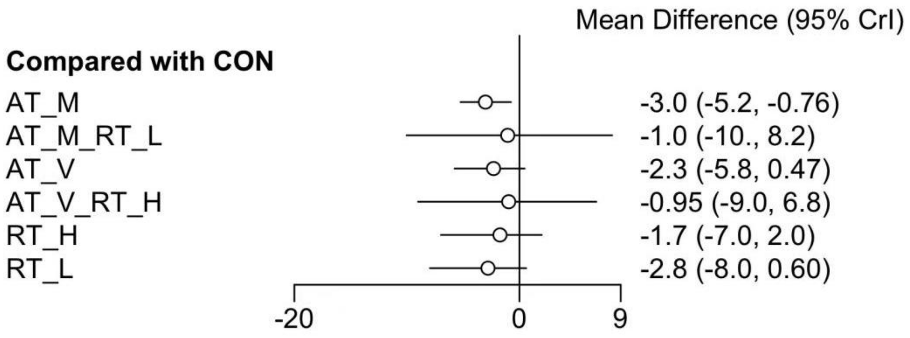

Figure 10.9.7: Forest plot overall change in general symptoms adjusted for time of single session 50 minutes. Exercise type are ranked according to MD compared to CON. Treatments crossing the y-axis are not significantly different from CON. *MD* Mean Difference, *RT-L* Resistance training of low-moderate load, *AT-M* Aerobic training of moderate intensity, *AT-M+RT-L* Combined moderate intensity aerobic exercise with low to moderate load resistance training, *AT-V* Aerobic training of vigorous intensity, *AT-V+RT-H* Combined vigorous intensity aerobic exercise with high load resistance training, *RT-H* Resistance training of high load.

10.10 LDL

10.10.1 Publication year

When the model was adjusted for centering value of publish year 2016, the hierarchy from the unadjusted model retained.

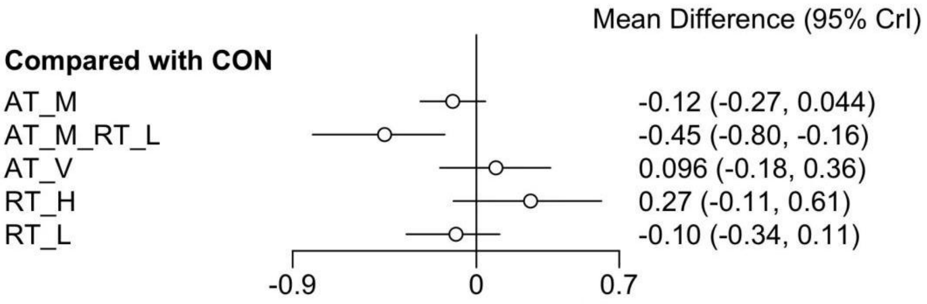

Figure 10.10.1: Forest plot overall change in general symptoms adjusted for time of publish year 2016. Exercise type are ranked according to MD compared to CON. Treatments crossing the y-axis are not significantly different from CON. *MD* Mean Difference, *RT-L* Resistance training of low-moderate load, *AT-M* Aerobic training of moderate intensity, *AT-M+RT-L* Combined moderate intensity aerobic exercise with low to moderate load resistance training, *AT-V* Aerobic training of vigorous intensity, *RT-H* Resistance training of high load.

10.10.2 Sample size

When the model was adjusted for centering value of sample size 101, hierarchy from the unadjusted model retained.

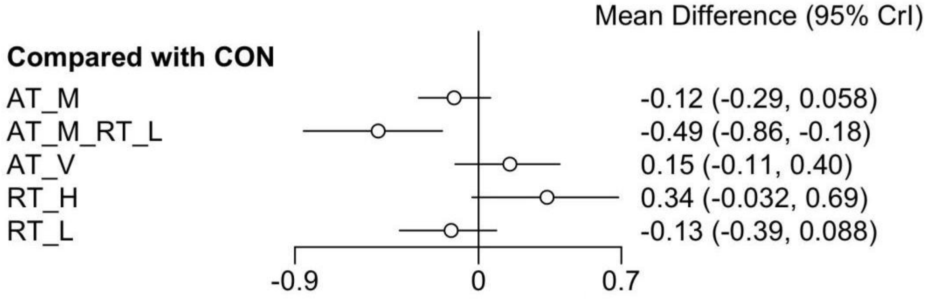

Figure 10.10.2: Forest plot overall change in general symptoms adjusted for time of sample size 101. Exercise type are ranked according to MD compared to CON. Treatments crossing the y-axis are not significantly different from CON. *MD* Mean Difference, *RT-L* Resistance training of low-moderate load, *AT-M* Aerobic training of moderate intensity, *AT-M+RT-L* Combined moderate intensity aerobic exercise with low to moderate load resistance training, *AT-V* Aerobic training of vigorous intensity, *RT-H* Resistance training of high load.

10.10.3 Percentage of male

When the model was adjusted for centering value of male's percentage 37%, hierarchy from the unadjusted model retained.

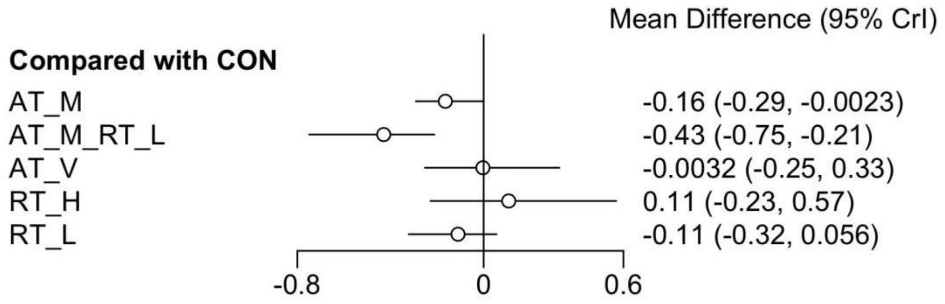

Figure 10.10.3: Forest plot overall change in general symptoms adjusted for time of male's percentage 37%. Exercise type are ranked according to MD compared to CON. Treatments crossing the y-axis are not significantly different from CON. *MD* Mean Difference, *RT-L* Resistance training of low-moderate load, *AT-M* Aerobic training of moderate intensity, *AT-M+RT-L* Combined moderate intensity aerobic exercise with low to moderate load resistance training, *AT-V* Aerobic training of vigorous intensity, *RT-H* Resistance training of high load.

10.10.4 Mean age

When the model was adjusted for centering value of mean age 60, the hierarchy from the unadjusted model retained.

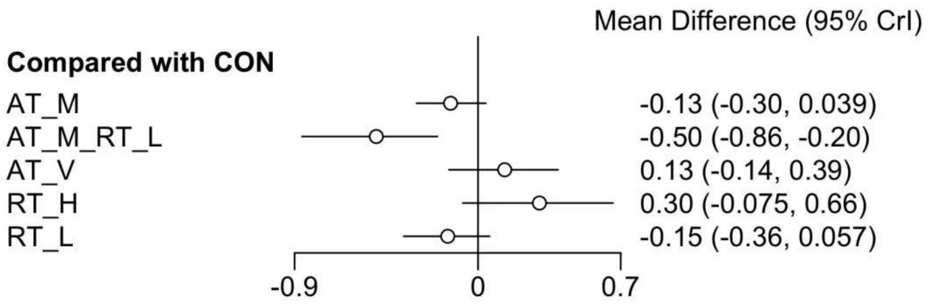

Figure 10.10.4: Forest plot overall change in general symptoms adjusted for time of mean age 60. Exercise type are ranked according to MD compared to CON. Treatments crossing the y-axis are not significantly different from CON. *MD* Mean Difference, *RT-L* Resistance training of low-moderate load, *AT-M* Aerobic training of moderate intensity, *AT-M+RT-L* Combined moderate intensity aerobic exercise with low to moderate load resistance training, *AT-V* Aerobic training of vigorous intensity, *RT-H* Resistance training of high load.

10.10.5 Exercise frequency

When the model was adjusted for centering value of exercise frequency 3(times/week), the hierarchy from the unadjusted model retained.

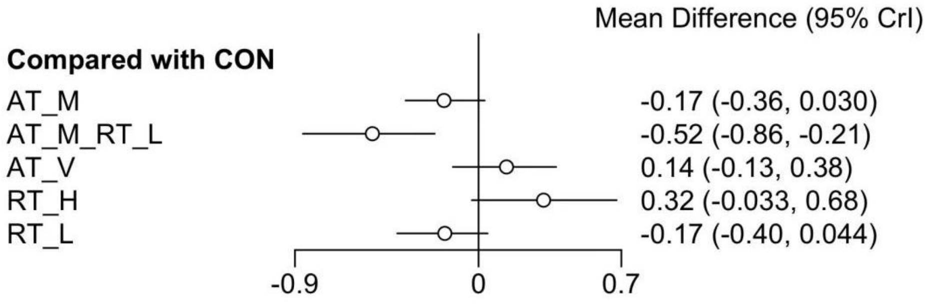

Figure 10.10.5: Forest plot overall change in general symptoms adjusted for time of exercise frequency 3(times/week). Exercise type are ranked according to MD compared to CON. Treatments crossing the y-axis are not significantly different from CON. *MD* Mean Difference, *RT-L* Resistance training of low-moderate load, *AT-M* Aerobic training of moderate intensity, *AT-M+RT-L* Combined moderate intensity aerobic exercise with low to moderate load resistance training, *AT-V* Aerobic training of vigorous intensity, *RT-H* Resistance training of high load.

10.10.6 Exercise period

When the model was adjusted for centering value of exercise period 47(weeks), the hierarchy from the unadjusted model retained.

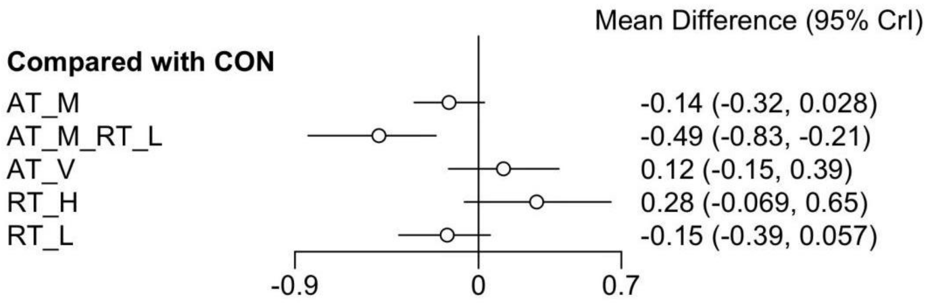

Figure 10.10.6: Forest plot overall change in general symptoms adjusted for time of exercise period 47(weeks). Exercise type are ranked according to MD compared to CON. Treatments crossing the y-axis are not significantly different from CON. *MD* Mean Difference, *RT-L* Resistance training of low-moderate load, *AT-M* Aerobic training of moderate intensity, *AT-M+RT-L* Combined moderate intensity aerobic exercise with low to moderate load resistance training, *AT-V* Aerobic training of vigorous intensity, *RT-H* Resistance training of high load.

10.10.7 Time of single session

When the model was adjusted for centering value of time of single session 51 minutes, the hierarchy from the unadjusted model retained.

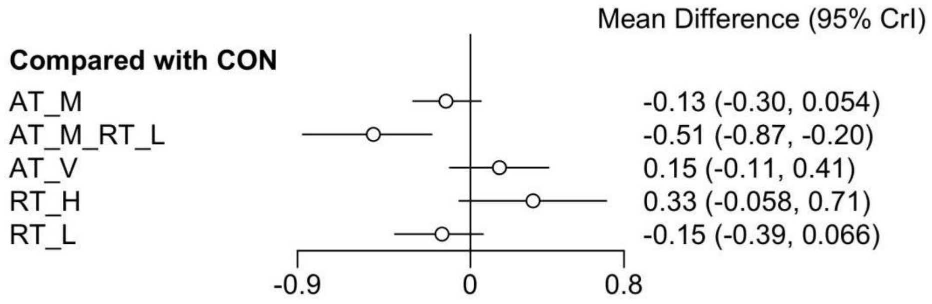

Figure 10.10`.7: Forest plot overall change in general symptoms adjusted for time of single session 51 minutes. Exercise type are ranked according to MD compared to CON. Treatments crossing the y-axis are not significantly different from CON. *MD* Mean Difference, *RT-L* Resistance training of low-moderate load, *AT-M* Aerobic training of moderate intensity, *AT-M+RT-L* Combined moderate intensity aerobic exercise with low to moderate load resistance training, *AT-V* Aerobic training of vigorous intensity, *RT-H* Resistance training of high load.

Appendix 11: subgroups analysis

11.1 FBG

11.1.1 Mean Age < 60

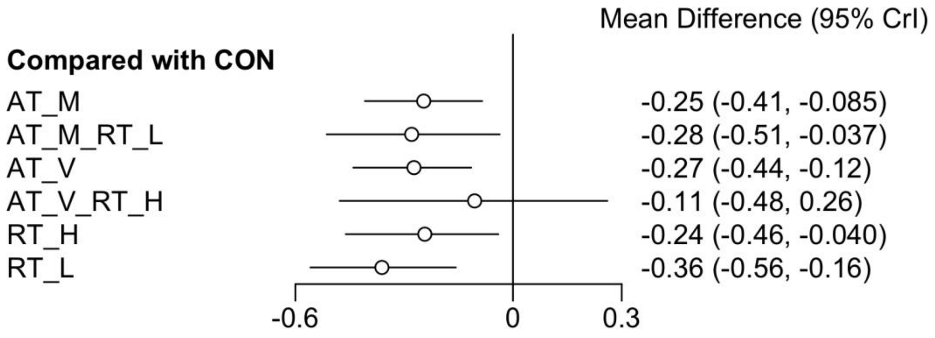

Figure 11.1.1: *MD* Mean Difference, *CON* control, *RT-L* Resistance training of low-moderate load, *AT-M* Aerobic training of moderate intensity, *AT-M+RT-L* Combined moderate intensity aerobic exercise with low to moderate load resistance training, *AT-V* Aerobic training of vigorous intensity, *AT-V+RT-H* Combined vigorous intensity aerobic exercise with high load resistance training, *RT-H* Resistance training of high load.

11.1.2 Mean Age ≥ 60

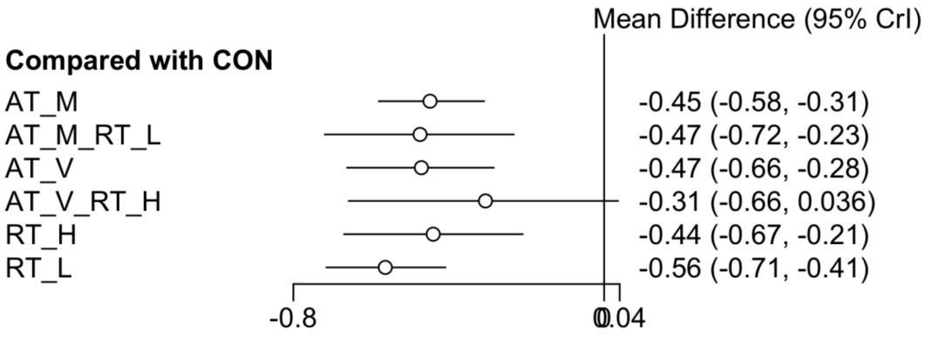

Figure 11.2: *MD* Mean Difference, *CON* control, *RT-L* Resistance training of low-moderate load, *AT-M* Aerobic training of moderate intensity, *AT-M+RT-L* Combined moderate intensity aerobic exercise with low to moderate load resistance training, *AT-V* Aerobic training of vigorous intensity, *AT-V+RT-H* Combined vigorous intensity aerobic exercise with high load resistance training, *RT-H* Resistance training of high load.

Table 11.1 Comparison of subgroup analysis

Original results			Mean Age < 60			Mean Age ≥ 60		
Contrast to CON	MD	95%Crl	Contrast to CON	MD	95%Crl	Contrast to CON	MD	95%Crl
RT-L	-0.55	[-0.68,-0.42]	RT-L	-0.36	[-0.56,-0.16]	RT-L	-0.56	[-0.71,-0.41]
AT-M	-0.39	[-0.48,-0.30]	AT-M+RT-L	-0.28	[-0.51,-0.037]	AT-V	-0.47	[-0.66,-0.28]
AT-M+RT-L	-0.39	[-0.58,-0.19]	AT-V	-0.27	[-0.44,-0.12]	AT-M+RT-L	-0.47	[-0.72,-0.23]
AT-V	-0.32	[-0.45,-0.19]	AT-M	-0.25	[-0.41,-0.12]	AT-M	-0.45	[-0.58,-0.31]
AT-V+RT-H	-0.22	[-0.51,0.07]	RT-H	-0.24	[-0.46,-0.040]	RT-H	-0.44	[-0.67,-0.21]
RT-H	-0.22	[-0.40,-0.04]	AT-V+RT-H	-0.11	[-0.48,0.26]	AT-V+RT-H	-0.31	[-0.66,0.036]

11.2 2hPG

11.2.1 Sample Size < 80

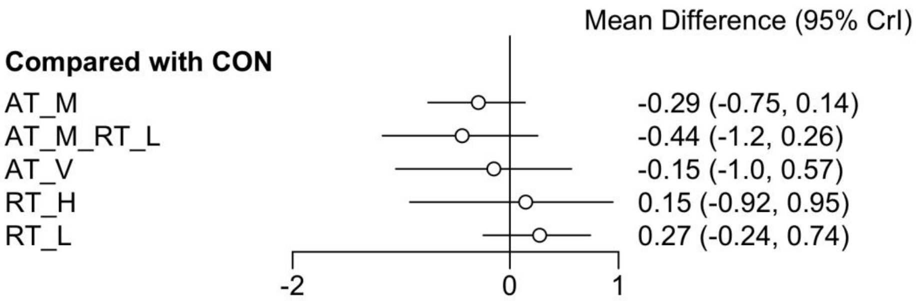

Figure 11.2.1: *MD* Mean Difference, *CON* control, *RT-L* Resistance training of low-moderate load, *AT-M* Aerobic training of moderate intensity, *AT-M+RT-L* Combined moderate intensity aerobic exercise with low to moderate load resistance training, *AT-V* Aerobic training of vigorous intensity, *RT-H* Resistance training of high load.

11.2.2 Sample Size ≥ 80

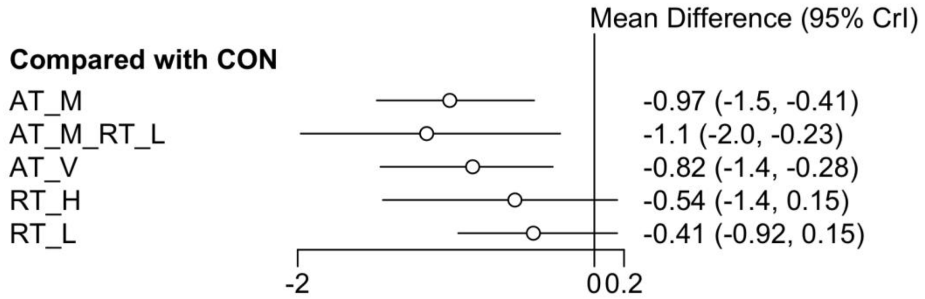

Figure 11.2.2: *MD* Mean Difference, *CON* control, *RT-L* Resistance training of low-moderate load, *AT-M* Aerobic training of moderate intensity, *AT-M+RT-L* Combined moderate intensity aerobic exercise with low to moderate load resistance training, *AT-V* Aerobic training of vigorous intensity, *RT-H* Resistance training of high load.

Table 11.2 Comparison of subgroup analysis

Original results			Sample Size < 80			Sample Size ≥ 80		
Contrast to CON	MD	95%Crl	Contrast to CON	MD	95%Crl	Contrast to CON	MD	95%Crl
AT-V	-0.69	[-1.28,-0.10]	AT-M+RT-L	-0.44	[-1.20,0.26]	AT-M+RT-L	-1.1	[-2.0,-0.23]
AT-M	-0.57	[-0.94,-0.19]	AT-M	-0.29	[-0.75,0.14]	AT-M	-0.97	[-1.5,-0.41]
AT-M+RT-L	-0.54	[-1.31,0.23]	AT-V	-0.15	[-1.00,0.57]	AT-V	-0.82	[-1.4,-0.23]
RT-H	-0.33	[-1.13,0.46]	RT-H	0.15	[-0.92,0.95]	RT-H	-0.54	[-1.4,-0.15]
RT-L	-0.28	[-0.69,0.14]	RT-L	0.27	[-0.24,0.74]	RT-L	-0.41	[-0.92,0.15]

11.3 HbA1c

11.3.1 Exercise Period < 44

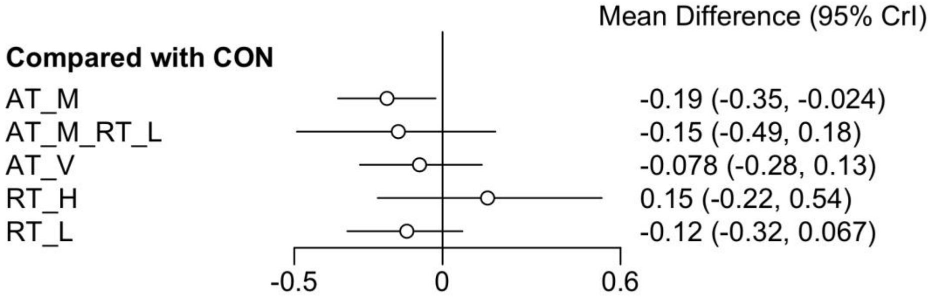

Figure 11.3.1: *MD* Mean Difference, *CON* control, *RT-L* Resistance training of low-moderate load, *RT-H* Resistance training of high load, *AT-M* Aerobic training of moderate intensity, *AT-M+RT-L* Combined moderate intensity aerobic exercise with low to moderate load resistance training, *AT-V* Aerobic training of vigorous intensity.

11.3.2 Exercise Period ≥ 44

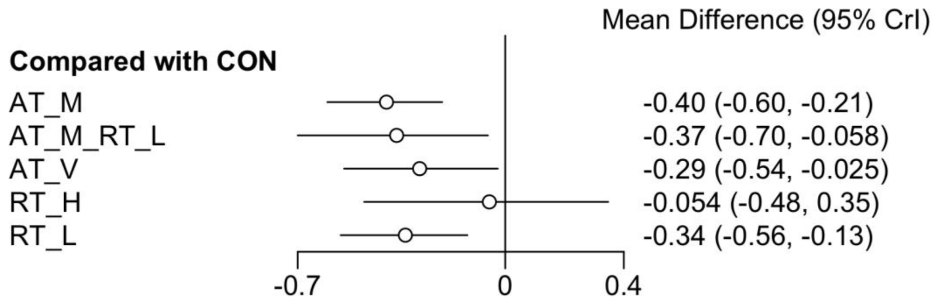

Figure 11.3.2: *MD* Mean Difference, *CON* control, *RT-L* Resistance training of low-moderate load, *RT-H* Resistance training of high load, *AT-M* Aerobic training of moderate intensity, *AT-M+RT-L* Combined moderate intensity aerobic exercise with low to moderate load resistance training, *AT-V* Aerobic training of vigorous intensity.

Table 11.3 Comparison of subgroup analysis

Original results			Sample Size < 80			Sample Size ≥ 80		
Contrast to CON	MD	95%Crl	Contrast to CON	MD	95%Crl	Contrast to CON	MD	95%Crl
AT-M+RT-L	-0.31	[-0.51,-0.10]	AT-M	-0.19	[-0.35,-0.0024]	AT-M	-0.40	[-0.60,-0.21]
RT-L	-0.28	[-0.38,-0.18]	AT-M+RT-L	-0.15	[-0.49,0.18]	AT-M+RT-L	-0.37	[-0.70,-0.058]
AT-M	-0.24	[-0.32,-0.16]	RT-L	-0.15	[-0.22,0.54]	RT-L	-0.34	[-0.56,-0.13]
AT-V	-0.18	[-0.31,-0.05]	RT-H	-0.12	[-0.32,0.067]	AT-V	-0.29	[-0.54,-0.0025]
RT-H	-0.09	[-0.29,0.11]	AT-V	-0.078	[-0.28,0.13]	RT-H	-0.054	[-0.48,0.35]

11.4 BMI

11.4.1 Exercise Period < 22

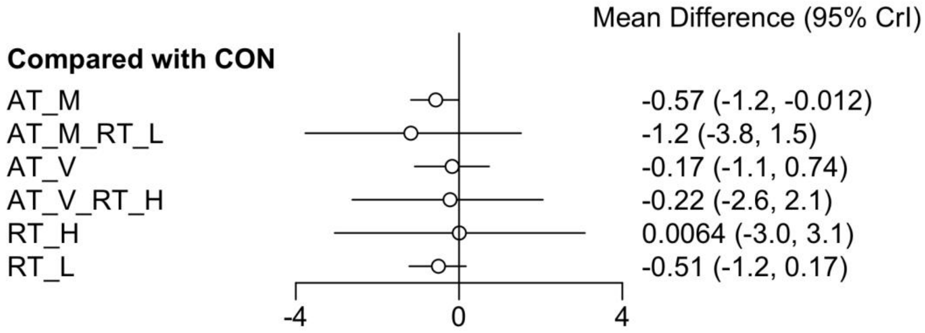

Figure 11.4.1: *MD* Mean Difference, *CON* control, *RT-L* Resistance training of low-moderate load, *AT-M* Aerobic training of moderate intensity, *AT-M+RT-L* Combined moderate intensity aerobic exercise with low to moderate load resistance training, *AT-V* Aerobic training of vigorous intensity, *AT-V+RT-H* Combined vigorous intensity aerobic exercise with high load resistance training, *RT-H* Resistance training of high load.

11.4.2 Exercise Period ≥ 22

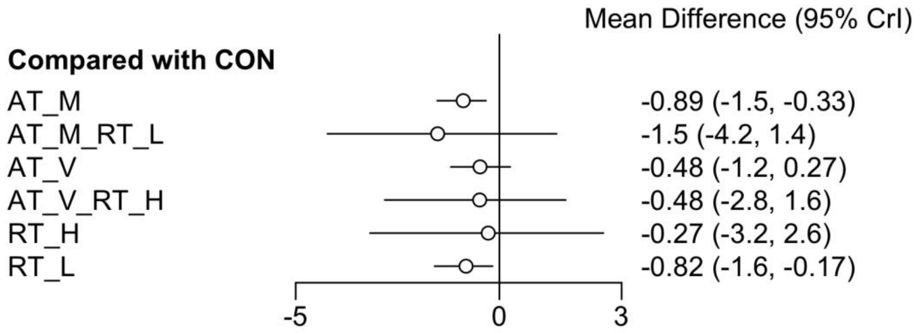

Figure 11.4.2: *MD* Mean Difference, *CON* control, *RT-L* Resistance training of low-moderate load, *AT-M* Aerobic training of moderate intensity, *AT-M+RT-L* Combined moderate intensity aerobic exercise with low to moderate load resistance training, *AT-V* Aerobic training of vigorous intensity, *AT-V+RT-H* Combined vigorous intensity aerobic exercise with high load resistance training, *RT-H* Resistance training of high load.

Table 11.4 Comparison of subgroup analysis

Original results			Exercise Period < 22			Exercise Period ≥ 22		
Contrast to CON	MD	95%Crl	Contrast to CON	MD	95%Crl	Contrast to CON	MD	95%Crl
AT-M+RT-L	-1.20	[-3.76,1.36]	AT-M+RT-L	-1.20	[-3.80,1.50]	AT-M+RT-L	-1.5	[-4.20,1.40]
AT-M	-0.71	[-1.00,-0.42]	AT-M	-0.57	[-1.20,-0.01]	AT-M	-0.89	[-1.50,-0.33]
RT-L	-0.61	[-0.97,-0.25]	RT-L	-0.51	[-1.20,0.17]	RT-L	-0.82	[-1.60,-0.17]
AT-V+RT-H	-0.50	[-2.35,1.35]	AT-V+RT-H	-0.22	[-2.60,2.10]	AT-V	-0.48	[-1.20,0.27]
AT-V	-0.41	[-0.94,0.13]	AT-V	-0.17	[-1.10,0.74]	AT-V+RT-H	-0.48	[-2.80,1.60]
RT-H	-0.20	[-3.10,2.70]	RT-H	0.01	[-3.00,3.10]	RT-H	-0.27	[-3.20,2.60]

11.5 TC

11.5.1 Publish Year < 2014

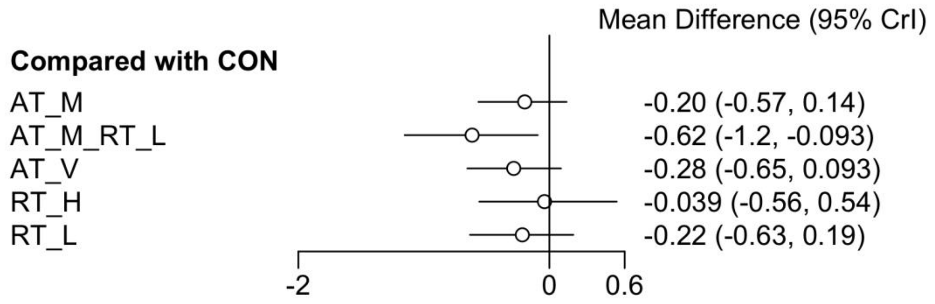

Figure 11.5.1: *MD* Mean Difference, *CON* control, *RT-L* Resistance training of low-moderate load, *AT-M* Aerobic training of moderate intensity, *AT-M+RT-L* Combined moderate intensity aerobic exercise with low to moderate load resistance training, *AT-V* Aerobic training of vigorous intensity, *RT-H* Resistance training of high load.

11.5.2 Publish Year ≥ 2014

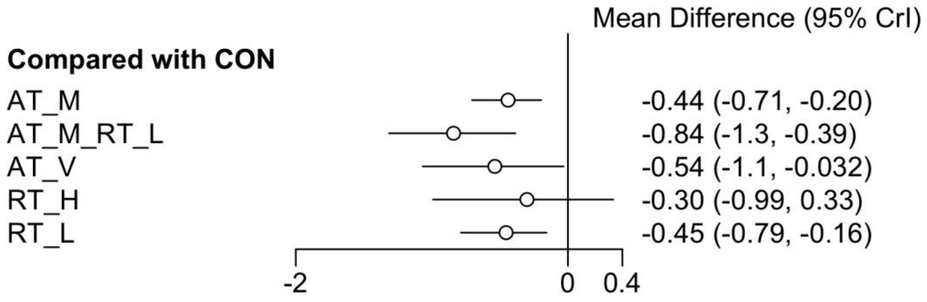

Figure 11.5.2: *MD* Mean Difference, *CON* control, *RT-L* Resistance training of low-moderate load, *AT-M* Aerobic training of moderate intensity, *AT-M+RT-L* Combined moderate intensity aerobic exercise with low to moderate load resistance training, *AT-V* Aerobic training of vigorous intensity, *RT-H* Resistance training of high load.

11.5.3 Exercise Period < 42

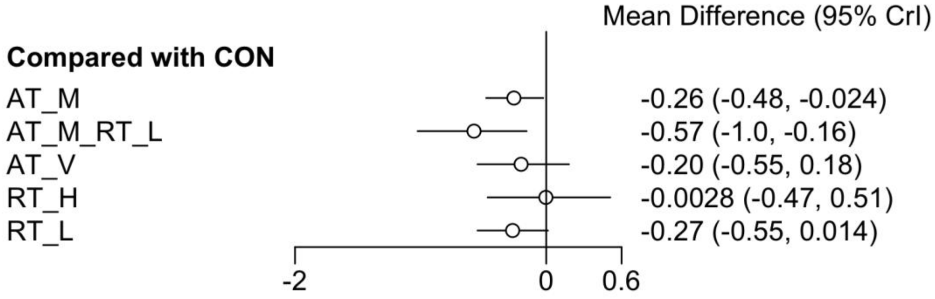

Figure 11.5.3: *MD* Mean Difference, *CON* control, *RT-L* Resistance training of low-moderate load, *AT-M* Aerobic training of moderate intensity, *AT-M+RT-L* Combined moderate intensity aerobic exercise with low to moderate load resistance training, *AT-V* Aerobic training of vigorous intensity, *RT-H* Resistance training of high load.

11.5.4 Exercise Period ≥ 42

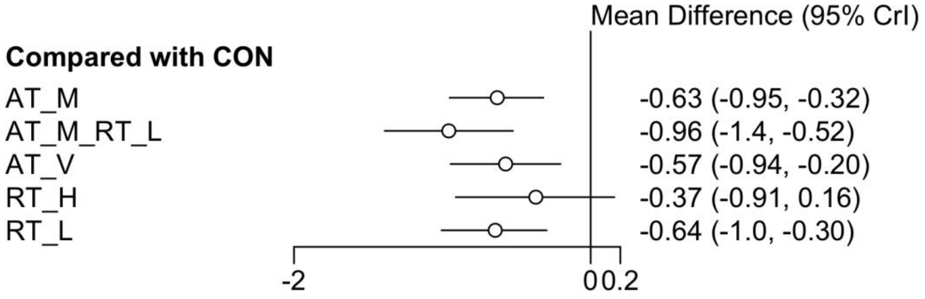

Figure 11.5.4: *MD* Mean Difference, *CON* control, *RT-L* Resistance training of low-moderate load, *AT-M* Aerobic training of moderate intensity, *AT-M+RT-L* Combined moderate intensity aerobic exercise with low to moderate load resistance training, *AT-V* Aerobic training of vigorous intensity, *RT-H* Resistance training of high load.

Table 11.5 Comparison of subgroup analysis

Original results			Publish Year < 2014			Publish Year ≥ 2014		
Contrast to CON	MD	95%Crl	Contrast to CON	MD	95%Crl	Contrast to CON	MD	95%Crl
AT-M+RT-L	-0.80	[-1.13,-0.46]	AT-M+RT-L	-0.62	[-3.80,1.50]	AT-M+RT-L	-0.84	[-1.30,-0.39]
AT-M	-0.34	[-0.49,-0.19]	AT-V	-0.28	[-1.20,-0.01]	AT-V	-0.54	[-1.10,-0.032]
RT-L	-0.34	[-0.54,-0.15]	RT-L	-0.22	[-1.20,0.17]	RT-L	-0.45	[-0.79,-0.16]
AT-V	-0.33	[-0.56,,-0.11]	AT-M	-0.20	[-2.60,2.10]	AT-M	-0.44	[-0.71,-0.20]
RT-H	-0.07	[-0.38,0.25]	RT-H	-0.039	[-1.10,0.74]	RT-H	-0.30	[-0.99,0.33]
			Exercise Period < 42			Exercise Period ≥ 42		
AT-M+RT-L	-0.80	[-1.13,-0.46]	AT-M+RT-L	-0.57	[-1.00,-0.16]	AT-M+RT-L	-0.96	[-1.40,-0.52]
AT-M	-0.34	[-0.49,-0.19]	RT-L	-0.27	[-0.55,0.014]	RT-L	-0.64	[-1.00,-0.30]
RT-L	-0.34	[-0.54,-0.15]	AT-M	-0.26	[-0.48,-0.024]	AT-M	-0.63	[-0.95,-0.32]
AT-V	-0.33	[-0.56,,-0.11]	AT-V	-0.20	[-0.55,0.18]	AT-V	-0.57	[-0.94,-0.20]
RT-H	-0.07	[-0.38,0.25]	RT-H	-0.0028	[-0.47,0.51]	RT-H	-0.37	[-0.91,0.16]

11.6 DBP

11.6.1 Publish Year < 2014

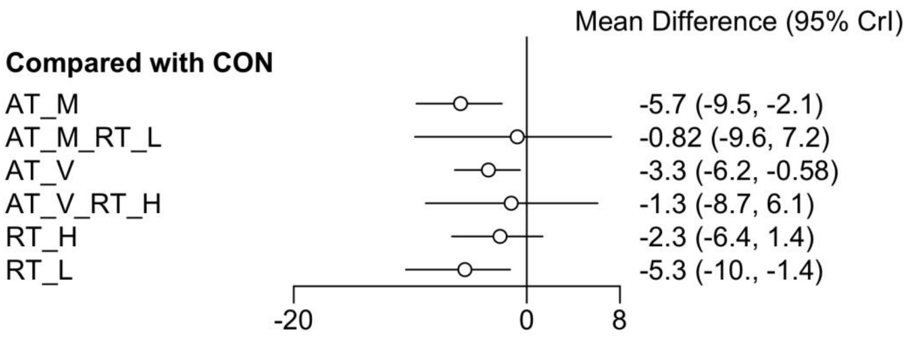

Figure 11.6.1: *MD* Mean Difference, *CON* control, *RT-L* Resistance training of low-moderate load, *AT-M* Aerobic training of moderate intensity, *AT-M+RT-L* Combined moderate intensity aerobic exercise with low to moderate load resistance training, *AT-V* Aerobic training of vigorous intensity, *AT-V+RT-H* Combined vigorous intensity aerobic exercise with high load resistance training, *RT-H* Resistance training of high load.

11.6.2 Publish Year ≥ 2014

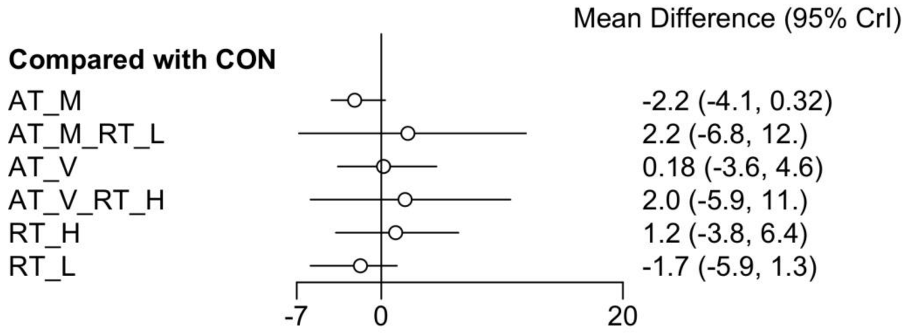

Figure 11.5.2: *MD* Mean Difference, *CON* control, *RT-L* Resistance training of low-moderate load, *AT-M* Aerobic training of moderate intensity, *AT-M+RT-L* Combined moderate intensity aerobic exercise with low to moderate load resistance training, *AT-V* Aerobic training of vigorous intensity, *AT-V+RT-H* Combined vigorous intensity aerobic exercise with high load resistance training, *RT-H* Resistance training of high load.

Table 11.6 Comparison of subgroup analysis

Original results			Publish Year < 2014			Publish Year ≥ 2014		
Contrast to CON	MD	95%Crl	Contrast to CON	MD	95%Crl	Contrast to CON	MD	95%Crl
AT-M	-3.43	[-5.39,-1.46]	AT-M	-5.70	[-9.50,-2.10]	AT-M	-2.20	[-4.10,0.32]
RT-L	-3.16	[-6.83,0.510]	RT-L	-5.30	[-10.0,-1.40]	RT-L	-1.70	[-5.90,1.30]
AT-V	-2.62	[-5.40,0.160]	AT-V	-3.30	[-6.20,-0.58]	AT-V	0.18	[-3.60,4.60]
RT-H	-1.88	[-6.21,2.44]	RT-H	-2.30	[-6.40,1.40]	RT-H	1.20	[-3.80,6.40]
AT-M+RT-L	-1.00	[-9.89;7.89]	AT-V+RT-H	-1.30	[-8.70,6.10]	AT-V+RT-H	2.00	[-5.90,1.30]
AT-V+RT-H	-1.01	[-8.11,6.09]	AT-M+RT-L	-0.82	[-9.60,7.20]	AT-M+RT-L	2.20	[-6.80,12.0]

Appendix 12: Publication bias

12.1 FBG

As shown in the figure below, the funnel plot had good symmetry, and the linear fitting line is not perpendicular to the 0 quadrant. Therefore, no small study effect was found for the primary outcome.

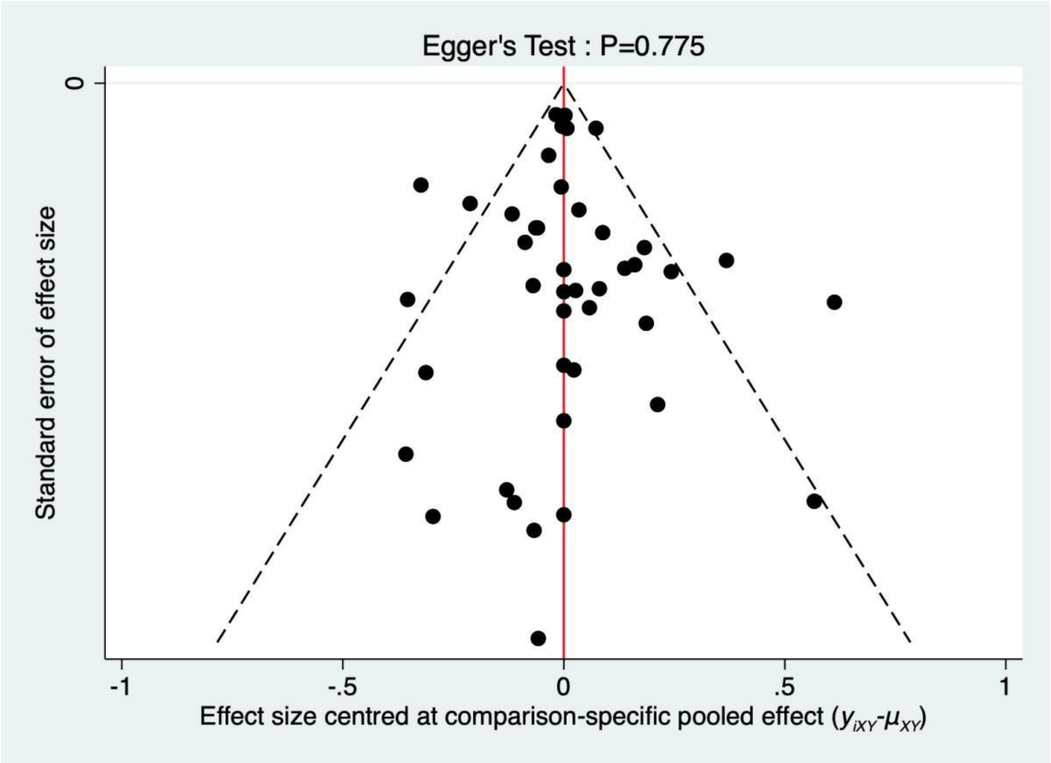

Figure 12.1: The funnel plot of change of general symptoms of all physical activity types compared to control group.

12.2 2hPG

As shown in the figure below, the funnel plot had good symmetry, and the linear fitting line is not perpendicular to the 0 quadrant. Therefore, no small study effect was found for the primary outcome.

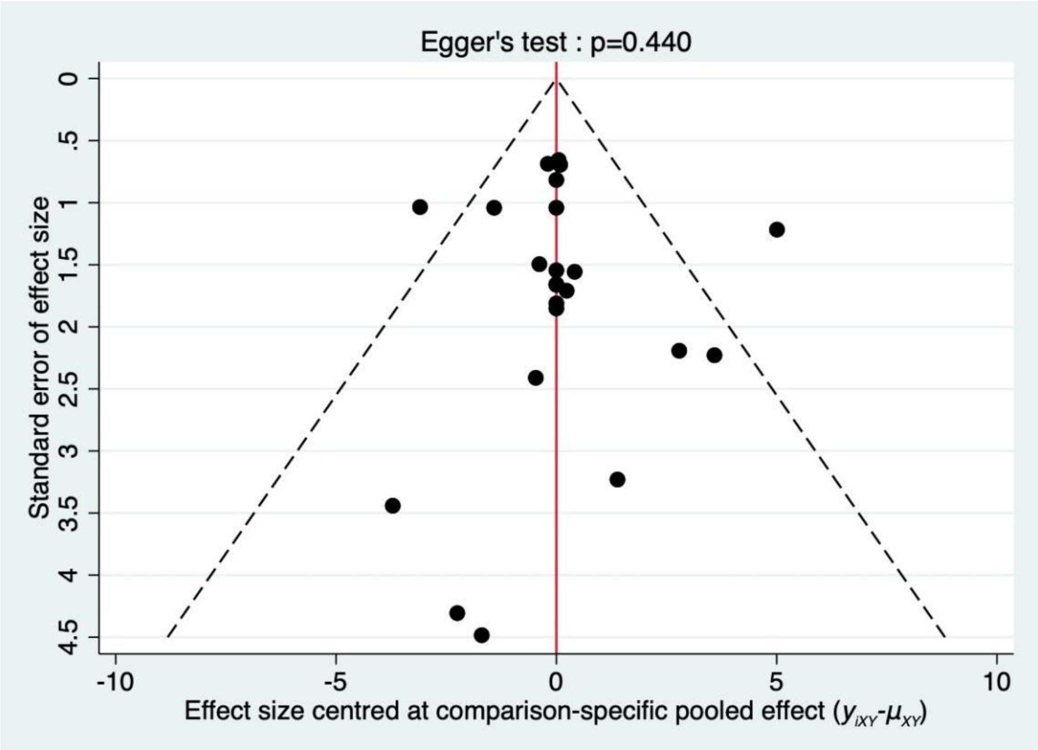

Figure 12.2: The funnel plot of change of general symptoms of all physical activity types compared to control group.

12.3 HbA1c

As shown in the figure below, the funnel plot had good symmetry, and the linear fitting line is not perpendicular to the 0 quadrant. Therefore, no small study effect was found for the primary outcome.

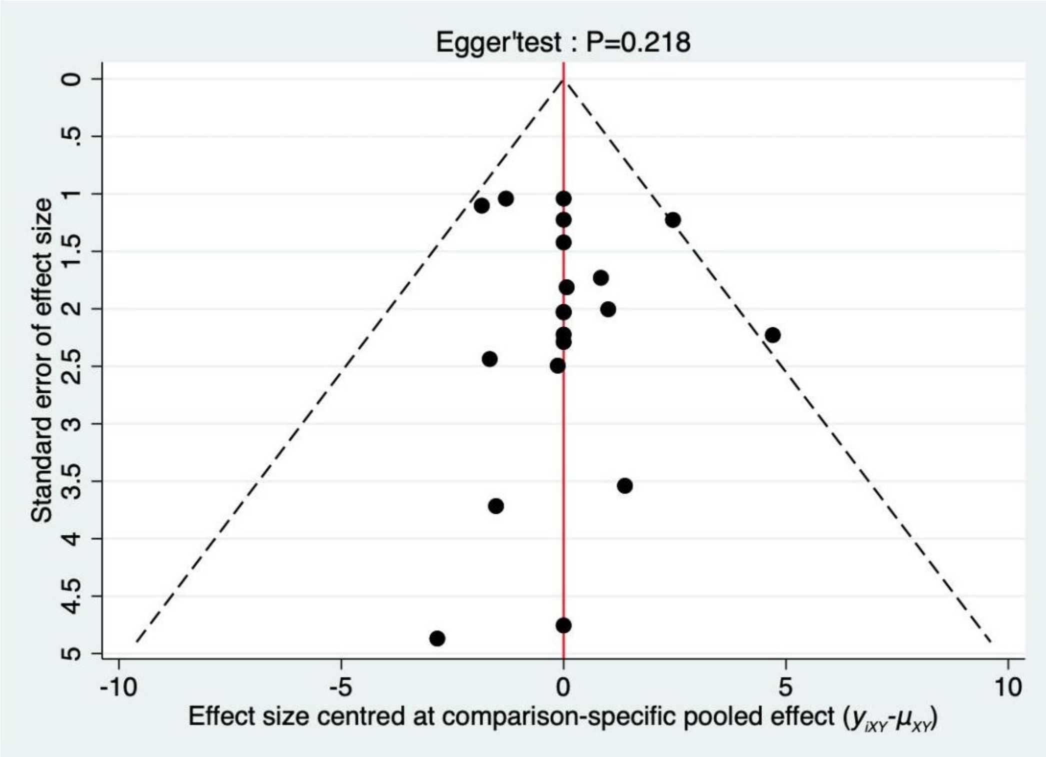

Figure 12.3: The funnel plot of change of general symptoms of all physical activity types compared to control group.

12.4 BMI

As shown in the figure below, the funnel plot had good symmetry, and the linear fitting line is not perpendicular to the 0 quadrant. Therefore, no small study effect was found for the primary outcome.

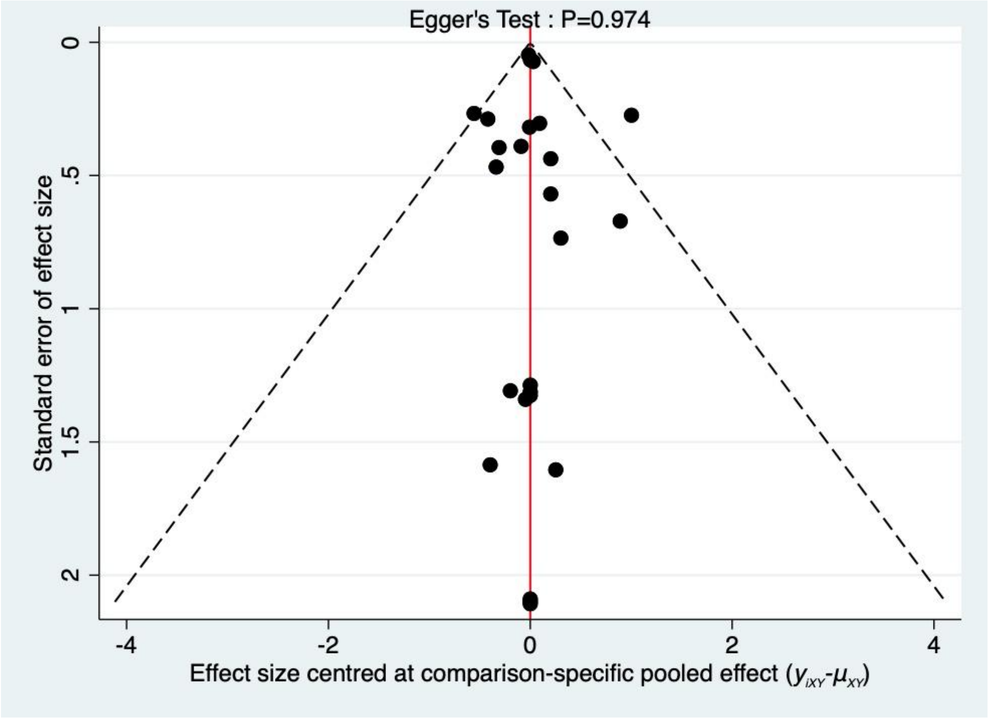

Figure 12.4: The funnel plot of change of general symptoms of all physical activity types compared to control group.

12.5 Weight

As shown in the figure below, the funnel plot had good symmetry, and the linear fitting line is not perpendicular to the 0 quadrant. Therefore, no small study effect was found for the primary outcome.

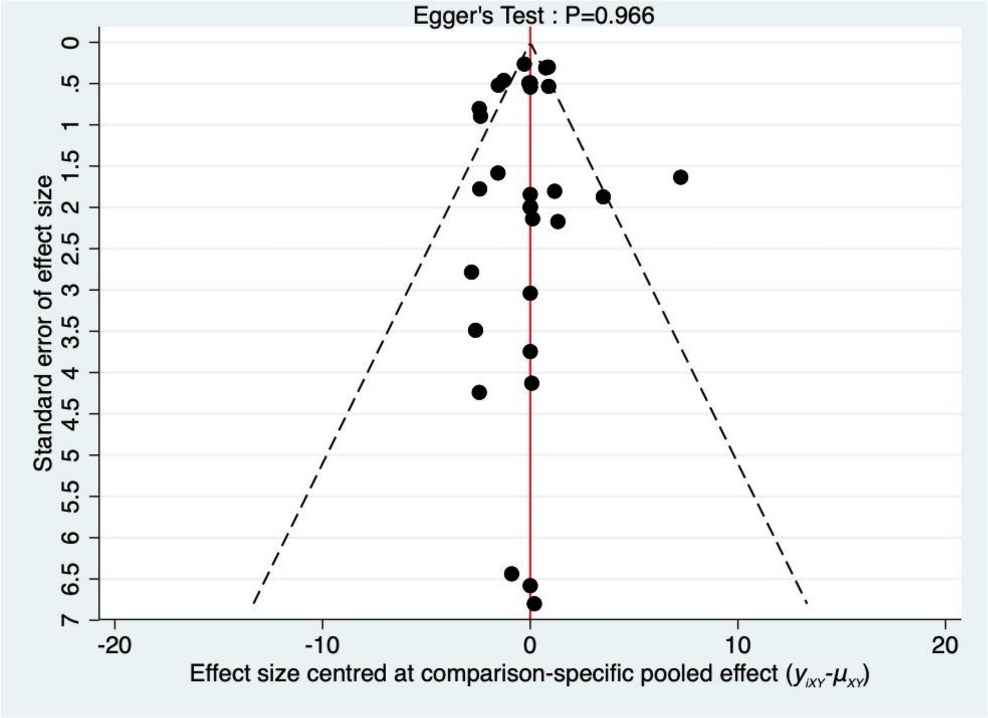

Figure 12.5: The funnel plot of change of general symptoms of all physical activity types compared to control group.

12.6 TC

As shown in the figure below, the funnel plot had good symmetry, and the linear fitting line is not perpendicular to the 0 quadrant. Therefore, no small study effect was found for the primary outcome.

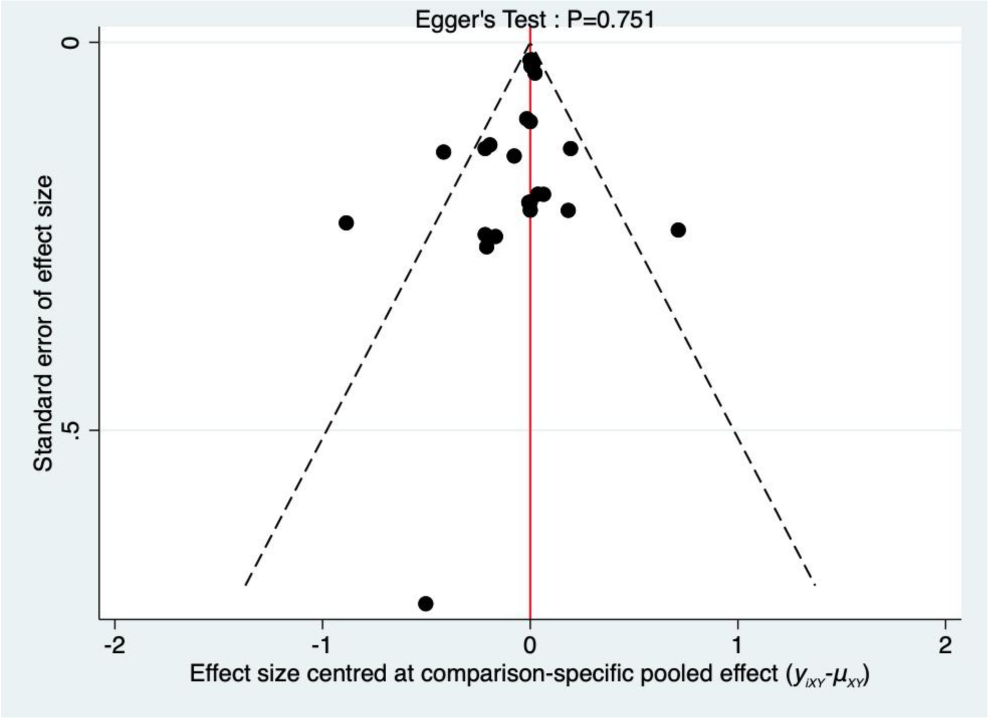

Figure 12.6: The funnel plot of change of general symptoms of all physical activity types compared to control group.

12.7 SBP

As shown in the figure below, the funnel plot had good symmetry, and the linear fitting line is not perpendicular to the 0 quadrant. Therefore, no small study effect was found for the primary outcome.

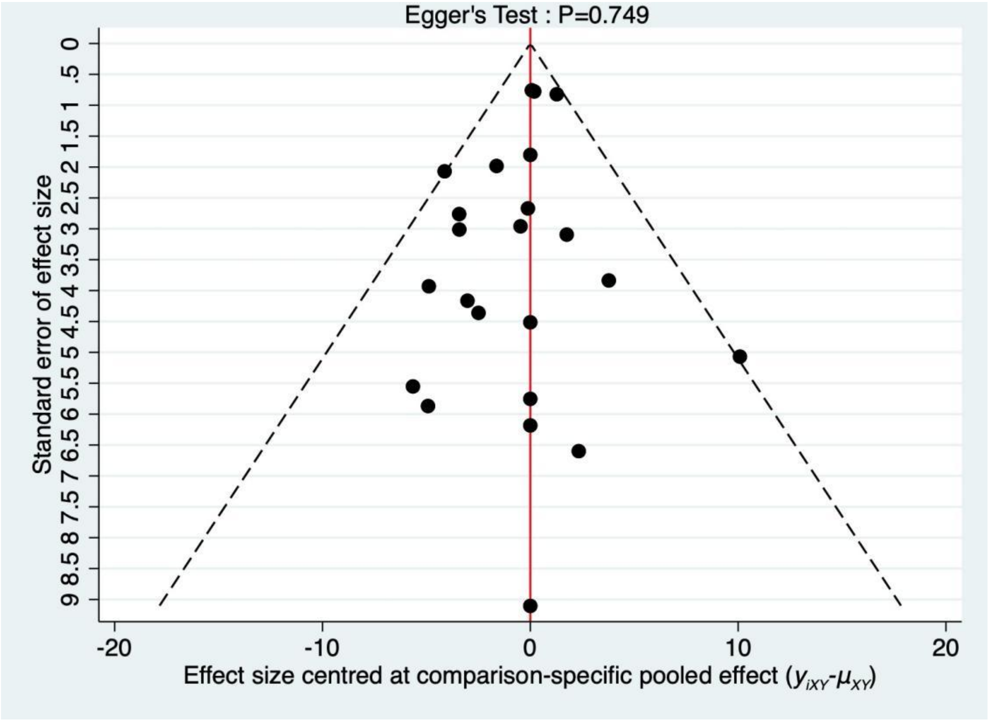

Figure 12.7: The funnel plot of change of general symptoms of all physical activity types compared to control group.

12.8 DBP

As shown in the figure below, the funnel plot had good symmetry, and the linear fitting line is not perpendicular to the 0 quadrant. Therefore, no small study effect was found for the primary outcome.

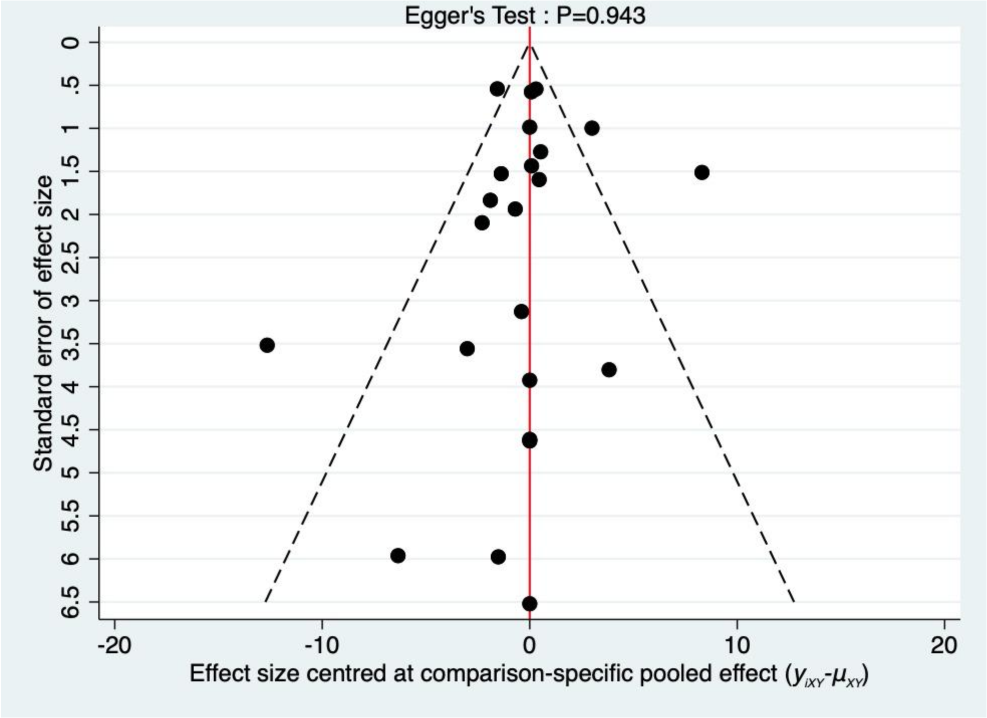

Figure 12.8: The funnel plot of change of general symptoms of all physical activity types compared to control group.

12.9 HDL

As shown in the figure below, the funnel plot had good symmetry, and the linear fitting line is not perpendicular to the 0 quadrant. Therefore, no small study effect was found for the primary outcome.

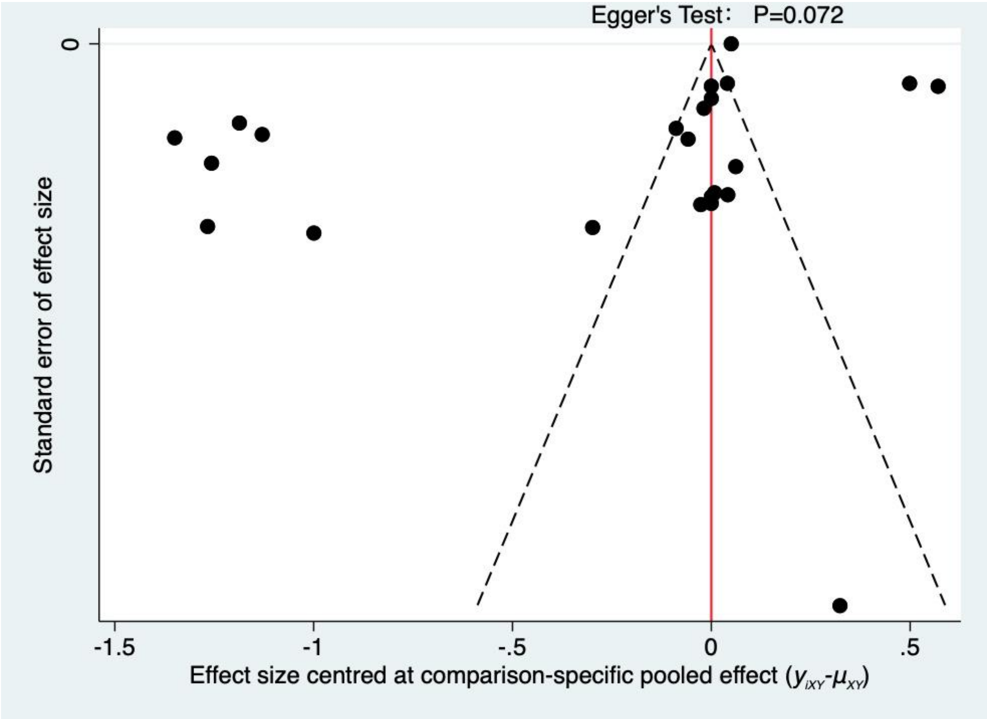

Figure 12.9: The funnel plot of change of general symptoms of all physical activity types compared to control group.

12.10 LDL

As shown in the figure below, the funnel plot had good symmetry, and the linear fitting line is not perpendicular to the 0 quadrant. Therefore, no small study effect was found for the primary outcome.

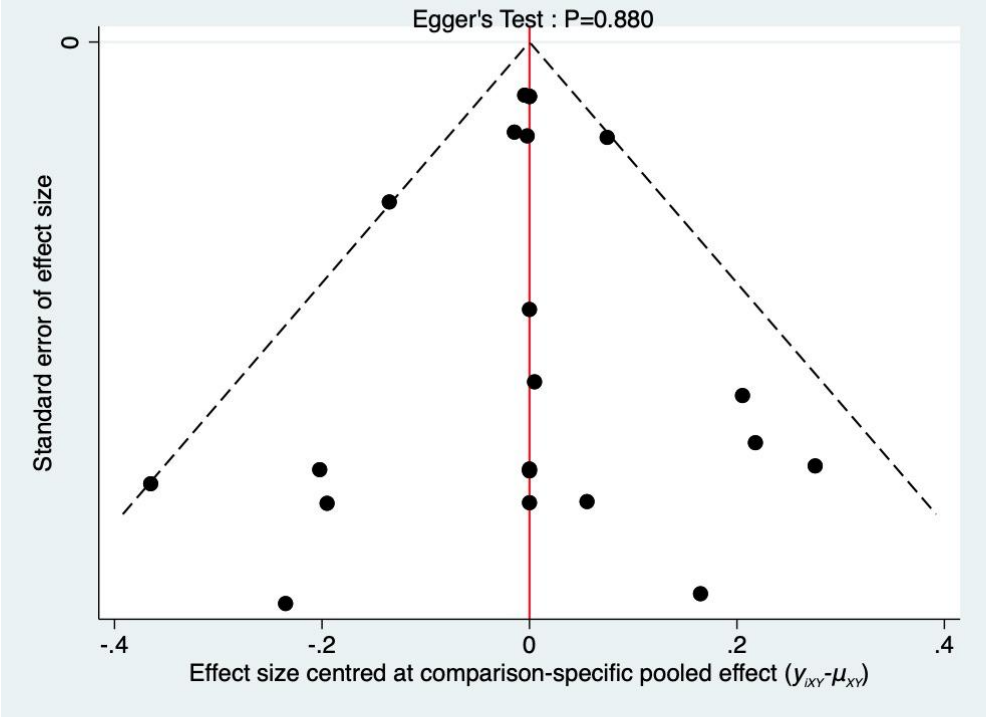

Figure 12.9: The funnel plot of change of general symptoms of all physical activity types compared to control group.
